# A counterion study of a series of [Cu(P^P)(N^N)][A] compounds with bis(phosphane) and 6-methyl and 6,6′-dimethyl-substituted 2,2′-bipyridine ligands for light-emitting electrochemical cells†

**DOI:** 10.1039/d1dt03239a

**Published:** 2021-11-02

**Authors:** Marco Meyer, Lorenzo Mardegan, Daniel Tordera, Alessandro Prescimone, Michele Sessolo, Henk J. Bolink, Edwin C. Constable, Catherine E. Housecroft

**Affiliations:** Department of Chemistry, University of Basel Mattenstrasse 24a BPR 1096 4058 Basel Switzerland catherine.housecroft@unibas.ch; Instituto de Ciencia Molecular (ICMol), Universidad de Valencia Catedrático José Beltrán 2 46980 Paterna Spain daniel.tordera@uv.es

## Abstract

The syntheses and characterisations of a series of heteroleptic copper(i) compounds [Cu(POP)(Mebpy)][A], [Cu(POP)(Me_2_bpy)][A], [Cu(xantphos)(Mebpy)][A] and [Cu(xantphos)(Me_2_bpy)][A] in which [A]^−^ is [BF_4_]^−^, [PF_6_]^−^, [BPh_4_]^−^ and [BAr^F^_4_]^−^ (Mebpy = 6-methyl-2,2′-bipyridine, Me_2_bpy = 6,6′-dimethyl-2,2′-bipyridine, POP = oxydi(2,1-phenylene)bis(diphenylphosphane), xantphos = (9,9-dimethyl-9*H*-xanthene-4,5-diyl)bis(diphenylphosphane), [BAr^F^_4_]^−^ = tetrakis(3,5-bis(trifluoromethyl)phenyl)borate) are reported. Nine of the compounds have been characterised by single crystal X-ray crystallography, and the consequences of the different anions on the packing interactions in the solid state are discussed. The effects of the counterion on the photophysical properties of [Cu(POP)(N^N)][A] and [Cu(xantphos)(N^N)][A] (N^N = Mebpy and Me_2_bpy) have been investigated. In the solid-state emission spectra, the highest energy emission maxima are for [Cu(xantphos)(Mebpy)][BPh_4_] and [Cu(xantphos)(Me_2_bpy)][BPh_4_] (*λ*^em^_max_ = 520 nm) whereas the lowest energy *λ*^em^_max_ values occur for [Cu(POP)(Mebpy)][PF_6_] and [Cu(POP)(Mebpy)][BPh_4_] (565 nm and 563 nm, respectively). Photoluminescence quantum yields (PLQYs) are noticeably affected by the counterion; in the [Cu(xantphos)(Me_2_bpy)][A] series, solid-state PLQY values decrease from 62% for [PF_6_]^−^, to 44%, 35% and 27% for [BF_4_]^−^, [BPh_4_]^−^ and [BAr^F^_4_]^−^, respectively. This latter series of compounds was used as active electroluminescent materials on light-emitting electrochemical cells (LECs). The luminophores were mixed with ionic liquids (ILs) [EMIM][A] ([EMIM]^+^ = [1-ethyl-3-methylimidazolium]^+^) containing the same or different counterions than the copper(i) complex. LECs containing [Cu(xantphos)(Me_2_bpy)][BPh_4_] and [Cu(xantphos)(Me_2_bpy)][BAr^F^_4_] failed to turn on under the LEC operating conditions, whereas those with the smaller [PF_6_]^−^ or [BF_4_]^−^ counterions had rapid turn-on times and exhibited maximum luminances of 173 and 137 cd m^−2^ and current efficiencies of 3.5 and 2.6 cd A^−1^, respectively, when the IL contained the same counterion as the luminophore. Mixing the counterions ([PF_6_]^−^ and [BF_4_]^−^) of the active complex and the IL led to a reduction in all the figures of merit of the LECs.

## Abstract 2

The choice of counterion in heteroleptic copper(i) luminophores for LECs has a critical effect on the photoluminescence quantum yields of the solid materials, and on the device figures-of-merit.

## Graphical abstract

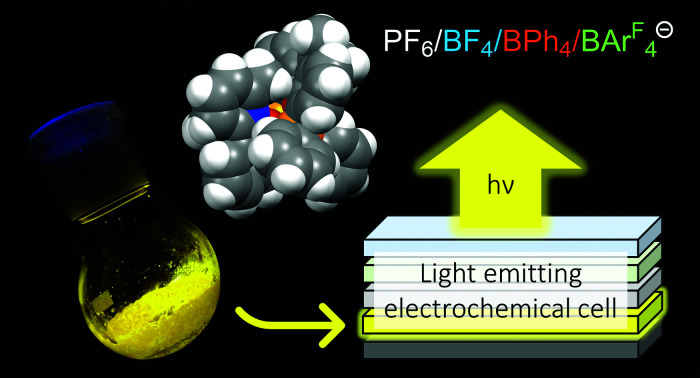

## Introduction

Lighting is a principal user of the world's energy, accounting for around 15% of global energy consumption.^1^ The move away from conventional light sources including incandescent lamps and fluorescent tubes is essential in terms of sustainability and conserving energy. Efficient solid-state lighting devices include both light-emitting diodes (LEDs) and organic light-emitting diodes (OLEDs) and, in Europe, these have largely superseded earlier technologies. Light-emitting electrochemical cells (LECs) are an alternative type of lighting device,^2^ which can achieve high luminance and high power efficiency while being operated at low voltage.^3–7^ Moreover, the architecture of LECs is less complex^8^ and is simpler to fabricate in comparison to state-of-the-art organic light-emitting diodes (OLEDs).^4^

The design of LECs allows the production of large-area lighting devices on substrates such as glass, metals and flexible materials including polymers, paper^9^ and textile fibres.^3,4,10–12^ LECs utilise mobile ionic species either as the luminophore or blended with the semiconductor within the same active layer.^2,3,13–15^ This layer functions as an ionic conductor.^5,16^ In its simplest implementation, the single-layer active composite is sandwiched between an air-stable cathode and anode.^2^ LECs are relatively insensitive in terms of the active layer thickness.^6,17,18^ Compared to OLEDs, less restriction is placed on the electrode materials because the use of low work-function metals is not required.^19^ During device fabrication, coating of the active layer onto the electrode substrate can be carried out under ambient conditions by solution-based techniques such as spin coating,^18,20,21^ spray sintering,^3^ inkjet printing^22,23^ and reel-to-reel^17,18^ depositions.^3^ Various types of compounds can be used as the emissive species, such as conjugated polymers,^3,11,24–28^ small molecules,^29–31^ quantum dots,^32–34^ perovskites^35–37^ and ionic transition metal complexes (iTMCs).^38–44^

iTMC-LECs have the advantage that the properties of the emissive complexes can be tuned in terms of emission wavelength, excited-state lifetime and quantum yield.^44,45^ LECs with iridium(iii)-based emitters^40,46–48^ have been shown to perform with high efficiencies, luminance values and lifetimes. More recently, Cu-iTMCs have proven to be promising emitting species.^49,50^ In contrast to iridium which is among the rarest elements on Earth, copper is abundant and inexpensive which translates to lower production costs and lower consumption of less sustainable elements.^51^ Among the most investigated types of copper(i)-based luminophores are [Cu(P^P)(N^N)]^+^ complexes where P^P is a chelating bisphosphane, usually a derivative of POP (POP = oxydi(2,1-phenylene)bis(diphenylphosphane)) or xantphos (xantphos = 9,9-dimethyl-9*H*-xanthene-4,5-diyl)(bis(diphenylphosphane)) and N^N is typically a derivative of bpy (bpy = 2,2′-bipyridine) or phen (phen = 1,10-phenanthroline).^13,21,50,52–54^

[Cu(P^P)(N^N)]^+^ complexes have the advantage of being suitable scaffolds for systematic investigations and tuning of energy levels of the frontier molecular orbitals.^55–57^ The spatial localization of the highest occupied molecular orbital (HOMO) has been calculated to mostly reside on the copper centre and partially on the bidentate bis(phosphane) ligand. The lowest unoccupied molecular orbital (LUMO), however, is localized on the diimine ligand.^55,58^ Thus, the two energy levels can be tailored independently depending on which of the two ligands is chemically modified. Ideally the two levels are attuned to facilitate charge injection as well as to enable recombination in the active layer.^59^ In photoexcitation, where the singlet–singlet transition is spin-allowed, singlet excited states are mostly accessed. During electroluminescence, electrons and holes recombine, each having its own spin. Due to spin statistics, the recombination yields 25% singlet and 75% triplet excitons.^60^ [Cu(P^P)(N^N)]^+^ complexes often exhibit thermally activated delayed fluorescence (TADF).^61^ With TADF, harvesting of all spin states of excitons after recombination is, in theory, possible. This leads to theoretical internal quantum efficiency (IQE) values of up to 100%. In contrast, with purely fluorescent emitters only 25% of the excitons can afford photon emission.^59,62^ We have previously demonstrated that [Cu(POP)(N^N)][PF_6_] and [Cu(xantphos)(N^N)][PF_6_] compounds, in which the N^N ligand is bpy or a methyl-substituted derivative, exhibit TADF.^20,63^

Currently the operational model of a LEC is described both by the electrochemical doping model (ECDM) and the electrodynamic model (EDM). Both theoretical mechanisms rely on mobile ions in the active layer. The two models are considered to coexist after comprehensive mathematical modelling of experimental data.^14,18,64,65^ The ECDM describes the growth of doped regions towards the centre of the emissive layer as an electric potential is applied across the two electrodes. Between the p- and n-doped region, an undoped intrinsic region constitutes the p-i-n junction where recombination takes place.^18,66^ The EDM depicts ions being attracted to the electrodes under the applied potential. The ions then form a double layer on the electrodes which shields the centre of the emissive layer from the electric field and facilitates charge injection. Injected charge carriers migrate into the centre where they recombine to form excitons and light is emitted.^45,66^ The preferential electrochemical doping model (PECDM) describes the behaviour if only one type of doping occurs. In reality, a combination of the models is thought to be operative as electric double layers are formed before doping takes place.^8,67,68^

**Scheme 1:**
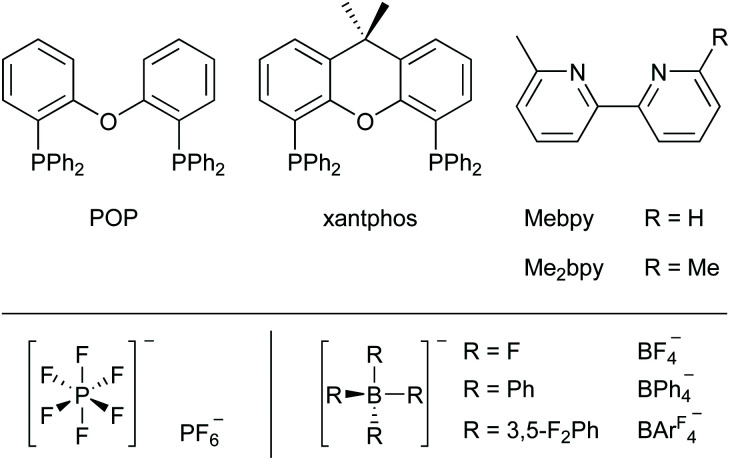
Top: Structures of the POP and xantphos P^P ligands and the Mebpy and Me_2_bpy N^N ligands. Bottom: Structures of the [PF_6_]^−^, [BF_4_]^−^, [BPh_4_]^−^ and [BAr^F^_4_]^−^ anions.

**Scheme 2:**
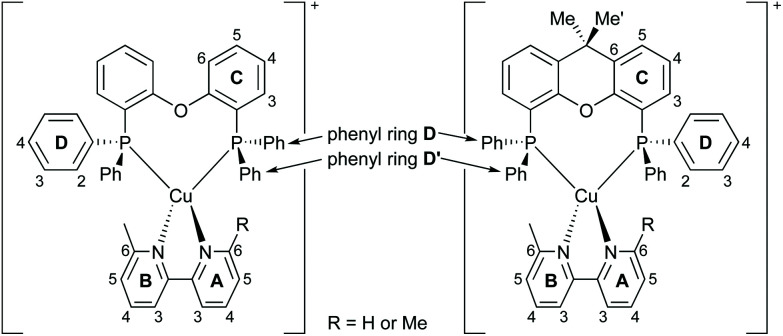
Structures of the [Cu(POP)(N^N)]^+^ and [Cu(xantphos)(N^N)]^+^ cations with ring and atom labelling for NMR spectroscopic data. When R = H, the rings are labelled **A** and **B** as shown. When R = Me, the pyridine rings are equivalent and are labelled **B**. Non-backbone phenyl rings in the P^P ligands are labelled **D**. The aromatic rings in the [BPh_4_]^−^ or [BAr^F^_4_]^−^ anions are labelled **E**.

It follows from the combination of the two models that the operation of a LEC strongly relies on the mobility of the ions distributed in the active layer. In turn, the mobility depends on the chemical and physical properties of the iTMC as well as the ionic liquid (IL) which is added to the active layer to promote this phenomenon. In the present work, we report a series of [Cu(P^P)(N^N)][A] complexes in which [A]^−^ is either [PF_6_]^−^, [BF_4_]^−^, [BPh_4_]^−^ or [BAr^F^_4_]^−^ ([BAr^F^_4_]^−^ = tetrakis(3,5-bis(trifluoromethyl)phenyl)borate) combined with different ionic liquids which are used in LECs. It has previously been reported that the choice of anion has a strong influence on the photophysical and structural characteristics of the Cu(i)-iTMCs.^69–71^ In the solid-state, molecular packing has a remarkable effect on the emissive properties of a luminophore, and it has been reported that pairing the complex cations with different counter ions can substantially alter the photoluminescence quantum yield (PLQY) of the salt.^71^ We were motivated to investigate the different characteristics and performances of the Cu(i)-complexes in different ILs within the LEC environment. The ILs were selected to have a consistent organic cation combined with anions that mimicked those in the Cu-iTMC. We report a family of 16 heteroleptic [Cu(P^P)(N^N)][A] complexes, subdivided into salts of four anions where P^P is either POP/xantphos and N^N represents either 6-methyl-2,2′-bipyridine (Mebpy) or 6,6′-dimethyl-2,2′-bipyridine (Me_2_bpy) (Schemes 1 and 2).

## Experimental

### General

Reactions under microwave conditions were carried out in a Biotage Initiator + microwave reactor. ^1^H, ^11^B{^1^H}, ^13^C{^1^H}, ^19^F{^1^H} and ^31^P{^1^H} NMR spectra were recorded at *ca*. 295 K in acetone-*d*_6_ using a Bruker Avance III-500 NMR spectrometer. ^1^H and ^13^C chemical shifts were referenced to residual solvent peaks (^1^H *δ*(acetone-d_5_) = 2.50 ppm, ^13^C *δ*(acetone-d_6_) = 29.84 ppm). Absorption and emission spectra in solution were measured using a Shimadzu UV-2600 spectrophotometer and a Shimadzu RF-5301-PC spectrofluorometer, respectively. A Shimadzu LCMS-2020 instrument was used to record electrospray (ESI) mass spectra. Quantum yields (dichloromethane (CH_2_Cl_2_) solution and powder) were measured using a Hamamatsu absolute photoluminescence (PL) quantum yield spectrometer C11347 Quantaurus-QY. Powder emission spectra and excited state lifetimes were measured with a Hamamatsu Compact Fluorescence lifetime Spectrometer C11367 Quantaurus-Tau using an LED light source (*λ*_exc_ = 365 nm). Lifetimes were obtained by fitting the measured data to an exponential decay using MATLAB®; a biexponential fit was used when a single exponential fit gave a poor fit. Where stated, the sample was degassed using argon bubbling for 20 min. PL spectra and PLQY measurements of the pure thin films were carried out using a Xe lamp and a monochromator as excitation source at 365 nm and an integrated sphere coupled to a spectrometer (Hamamatsu C9920-02 with a Hamamatsu PMA-11 optical detector). Electrochemical measurements used an Ametek VersaSTAT 3F potentiostat with [^*n*^Bu_4_N][PF_6_] (0.1 M) as supporting electrolyte and a scan rate of 0.1 V s^−1^; the solvent was dry propylene carbonate and solution concentrations were *ca*. 2 × 10^3^ mol dm^−3^. The solutions were constantly degassed with argon bubbling. The working electrode was glassy carbon, the reference electrode was a leakless Ag^+^/AgCl (eDAQ ET069-1, filling electrolyte aqueous KCl, conc. 3.4 mol L^−1^) and the counter-electrode was a platinum wire. Final potentials were internally referenced with respect to the Fc/Fc^+^ couple.

[Cu(MeCN)_4_][PF_6_] was prepared according to the literature procedure.^72^ Me_2_bpy was purchased from Fluorochem. 2-Pyridylzinc bromide, POP and xantphos were purchased from Acros Organics. 2-Chloro-6-methylpyridine and Na[BAr^F^_4_] were bought from Apollo Scientific, Na[BPh_4_] from Fluka, and [Pd(PPh_3_)_4_], [EMIM][PF_6_] and [EMIM][BF_4_] from Sigma Aldrich. Mebpy was prepared by a Negishi coupling reaction following a microwave reactor adaption^56^ of a literature method.^73^ The NMR spectroscopic data were consistent with those reported.^73^

### Syntheses and characterization of all copper(i) compounds

Details of syntheses, ^1^H, ^13^C{^1^H}, ^11^B{^1^H}, ^19^F{^1^H}, and ^31^P{^1^H} NMR characterization and assignments, electrospray mass spectrometric data, and elemental analyses are given in the ESI.†

### General procedures for [PF_6_]^−^ and [BF_4_]^−^ salts of the copper(i) complexes

POP-containing compounds were synthesized by the following procedure: POP (1.1 eq.) and [Cu(MeCN)_4_][PF_6_] or [Cu(MeCN)_4_][BF_4_] (1.0 eq.) were dissolved in CH_2_Cl_2_ (20 mL) and the reaction mixture was stirred for 1.5 h. The desired N^N ligand (1.0 eq.) was added, followed by stirring of the mixture for 2 h. The solvent was then removed under reduced pressure. The residue was purified by precipitation from CH_2_Cl_2_ with diethyl ether (Et_2_O), followed by centrifugation and decantation of the supernatant. This step was repeated four times. Then the product was washed with cyclohexane (100 mL).

Compounds containing xantphos were prepared according to the following procedure: a solution of the appropriate N^N-ligand (1.0 eq.) and xantphos (1.1 eq.) in CH_2_Cl_2_ (10 mL) was added dropwise to a CH_2_Cl_2_ solution (10 mL) of [Cu(MeCN)_4_][PF_6_] or [Cu(MeCN)_4_][BF_4_] (1.0 eq.). The reaction mixture was then stirred for 2 h before the solvent was removed under reduced pressure. The residue was purified by precipitation from CH_2_Cl_2_ with Et_2_O, followed by centrifugation and decantation of the supernatant. This step was repeated four times. Then the product was washed with cyclohexane (100 mL) and dried under high vacuum. Detailed experimental conditions are given in the ESI.†

### General procedures for [BPh_4_]^−^ and [BAr^F^_4_]^−^ salts of the copper(i) complexes

The following procedure was adapted from a literature method.^74^ To synthesise the [BPh_4_]^−^ and [BAr^F^_4_]^−^ salts, an ion exchange was carried out starting with the appropriate [PF_6_]^−^ salt.

The [PF_6_]^−^ salt (1.0 eq.) of the desired complex was dissolved in a minimal amount of MeOH at 45 °C while sonicating. Then NaBPh_4_ (1.3 eq.) or NaBAr^F^_4_ (1.3 eq.), respectively, was added to the warm solution. The mixture was sonicated and H_2_O (60 mL) was added to precipitate the product. The product was washed with H_2_O and dried under vacuum.

The products were purified as follows. The crude product was dissolved in CH_2_Cl_2_ (20 mL) and water was added (15 mL). The mixture was vigorously shaken, centrifuged and the aqueous phase was removed. The organic phase was dried with MgSO_4_. The product was precipitated from CH_2_Cl_2_ with Et_2_O, followed by centrifugation and decantation of the supernatant. This step was repeated three times. Then the product was washed with cyclohexane (100 mL) and dried under vacuum. Details of the conditions for each complex are given in the ESI.†

### Crystallography

Crystallographic data for all the compounds are presented in Table S1.† Single crystal data were collected on a Bruker APEX-II diffractometer (CuKα radiation, see Table S1†) with data reduction, solution and refinement using the programs APEX,^75^ ShelXT,^76^ Olex2,^77^ and ShelXL v. 2014/7,^78^ or using a STOE StadiVari diffractometer equipped with a Pilatus300K detector and with a Metaljet D2 source (GaKα radiation, see Table S1†) and solving the structure using Superflip,^79,80^ and Olex2.^77^ The structural model was refined with ShelXL v. 2014/7.^78^ Structure analysis used Mercury CSD v. 2021.1.0.^81^

In [Cu(xantphos)(Mebpy)][BF_4_]·CH_2_Cl_2_·Et_2_O, a solvent mask was used to treat the solvent region, and the removed electron density equated to one CH_2_Cl_2_ and one Et_2_O molecule per Cu, which have been added to all the formulae and metrics. In [Cu(xantphos)(Me_2_bpy)][BF_4_]·0.5C_6_H_12_·0.8Me_2_CO, part of the solvent region was treated with a solvent mask and 0.8 molecules of acetone were added to the formula and relevant data.

In [Cu(POP)(Mebpy)][PF_6_]·0.5CH_2_Cl_2_·0.3Et_2_O, the region of the solvent contained disordered CH_2_Cl_2_ and Et_2_O molecules which were modelled over two sites with partial occupancies 0.5 and 0.3, respectively. The dichloromethane molecules in [Cu(xantphos)(Mebpy)][PF_6_]·0.5CH_2_Cl_2_·Et_2_O were modelled with half occupancy sites. The anion in [Cu(xantphos)(Mebpy)][BF_4_]·CH_2_Cl_2_·Et_2_O was disordered and was modelled over two sites with a common B position, and F atoms in half-occupancy sites. In [Cu(POP)(Me_2_bpy)][BAr^F^_4_], three CF_3_ groups in the [BAr^F^_4_]^−^ anion were rotationally disordered; the CF_3_ groups with F24 and F26, and with F19 and F29 were each modelled over two sites of equal occupancies, and the CF_3_ group with F1, F20 and F31 was modelled over three sites of equal occupancies. In [Cu(POP)(Mebpy)][BAr^F^_4_]·C_6_H_12_, the disordered Mebpy ligand was modelled over two, equal occupancy sites. In addition, three of the CF_3_ groups in the [BAr^F^_4_]^−^ anion were rotationally disordered; the group containing F7 and F33 was modelled over sites with fractional occupancies of 0.65 and 0.35, respectively, and that with F14 and F34 was modelled over two equal occupancy sites, and the CF_3_ group with F19, F25 and F28 was modelled over three sites with fractional occupancies of 0.4, 0.4 and 0.2, respectively.

### Device preparation and characterization

Solutions of the copper(i) complexes were mixed in a molar ratio of 4 : 1 with an ionic liquid (IL) containing the same anion as the copper(i) compound. The ILs comprised [1-ethyl-3-methylimidazolium]^+^ ([EMIM]^+^) with [PF_6_]^−^, [BF_4_]^−^, [BPh_4_]^−^ and [BAr^F^_4_]^−^ counterions. The solutions of ILs were prepared in CH_2_Cl_2_ at a concentration of 10 mg mL^−1^. Solutions of the [Cu(xantphos)(Me_2_bpy)]^+^ series containing [Cu(xantphos)(Me_2_bpy)][PF_6_], [Cu(xantphos)(Me_2_bpy)][BF_4_], [Cu(xantphos)(Me_2_bpy)][BPh_4_] or [Cu(xantphos)(Me_2_bpy)][BAr^F^_4_] were prepared in CH_2_Cl_2_ to a final concentration of 15 mg mL^−1^. Dissolution in CH_2_Cl_2_ of both the iTMC and ionic liquids as well as the final mixed solution was instantaneous and no further heating and stirring was needed. Pre-patterned indium tin oxide (ITO)-coated glass plates were used as transparent conductive substrates. They were subsequently cleaned ultrasonically in soapy-water, water, and propan-2-ol baths. After drying, the substrates were placed in an UV-ozone cleaner (Jelight 42-220) for 20 min. The ITO substrates were first coated with PEDOT:PSS (PEDOT = poly(3,4-ethylenedioxythiophene), PSS = polystyrenesulfonate) and iTMC-IL solutions. Thicknesses of 100 nm and 80 nm were obtained respectively. Before depositing the light-emitting layer, the PEDOT:PSS layers were annealed on a hotplate at 150 °C for 10 minutes. The copper(i) complex thin films were then annealed at 70 °C for 30 minutes. Devices were also prepared by changing the counter ion of the IL added to the copper complex to study the effect of the anion size on the device performance. This was done by mixing [Cu(xantphos)(Me_2_bpy)][PF_6_] with [EMIM][BF_4_] and [Cu(xantphos)(Me_2_bpy)][BF_4_], [Cu(xantphos)(Me_2_bpy)] [BPh_4_] or [Cu(xantphos)(Me_2_bpy)][BAr^F^_4_] with [EMIM][PF_6_]. Finally, an Al electrode (100 nm) was thermally evaporated on top of the active layer using a shadow mask under inert atmosphere. The final active area of the cells was 6 mm^2^. The thickness of the PEDOT:PSS and the active layer were determined with an Ambios XP-1 profilometer. The devices were measured by applying a pulsed current density of 50 A m^−2^ while monitoring the voltage and luminance *versus* time by using a True Color Sensor MAZeT (MTCSiCT sensor) with a Botest OLT OLED Lifetime-Test system. The applied pulsed current consisted of block waves at a frequency of 1000 Hz with a duty cycle of 50%. Hence, the average current density and voltage were obtained by multiplying the values by the time-on (0.5 s) and dividing by the total cycle time (1 s). Electroluminescence (EL) spectra were recorded by driving the cells with the Botest OLT system and an optical fibre connected to the Avant spectrometer AvaSpec – 2048L.

## Results and discussion

### Synthesis of [Cu(P^P)(N^N)][PF_6_] and [Cu(P^P)(N^N)][BF_4_]

The different strategies for preparing heteroleptic [Cu(P^P)(N^N)][PF_6_] and [Cu(xantphos)(N^N)][BF_4_] complexes have been detailed previously.^21,53,55,58^ The formation of heteroleptic complexes over the kinetically more favoured homoleptic [Cu(N^N)_2_][A] complexes was ensured by the following optimizations. To obtain the heteroleptic [Cu(POP)(N^N)][A] complexes, 1.1 equivalents of POP were added to [Cu(MeCN)_4_][A], followed by the addition of the N^N ligand after 2 hours. Residual excess POP was removed in the purification steps (see Experimental section). To obtain the heteroleptic [Cu(xantphos)(N^N)][A] complexes, a solution containing both the N^N ligand and xantphos were added to a solution of [Cu(MeCN)_4_][A]. These procedures were followed to afford the [Cu(POP)(N^N)][A] and [Cu(xantphos)(N^N)][A] complexes (P^P = POP or xantphos; N^N = Mebpy or Me_2_bpy; [A]^−^ = [PF_6_]^−^ or [BF_4_]^−^) as yellow solids in yields of 72–94%.

### Synthesis of [Cu(P^P)(N^N)][BPh_4_] and [Cu(P^P)(N^N)][BAr^F^_4_]

The complexes containing [BPh_4_]^−^ and [BAr^F^_4_]^−^ anions were prepared by anion exchange starting from the corresponding [PF_6_]^−^ salt and adding an excess of NaBPh_4_ or NaBAr^F^_4_, respectively. For the [BPh_4_]^−^ salts, the anion exchange was conducted in two steps to ensure complete replacement of [PF_6_]^−^ ions. During the washing process, H_2_O was used to remove NaPF_6_ or NaBF_4_ generated in the ion exchange. In further purification steps, any remaining impurities were removed using Et_2_O and cyclohexane. [Cu(POP)(N^N)][A] and [Cu(xantphos)(N^N)][A] ([A]^−^ = [BPh_4_]^−^ or [BAr^F^_4_]^−^) were obtained as yellow solids in yields of 72–84%.

### Characterisation of the copper(i) complexes

The positive mode electrospray mass spectrum of each compound exhibited peak envelopes arising from the [Cu(POP)(N^N)]^+^ or [Cu(xantphos)(N^N)]^+^ cations as well as from the [Cu(POP)]^+^ or [Cu(xantphos)]^+^ cation (see Experimental section in ESI†).

**Fig. 1:**
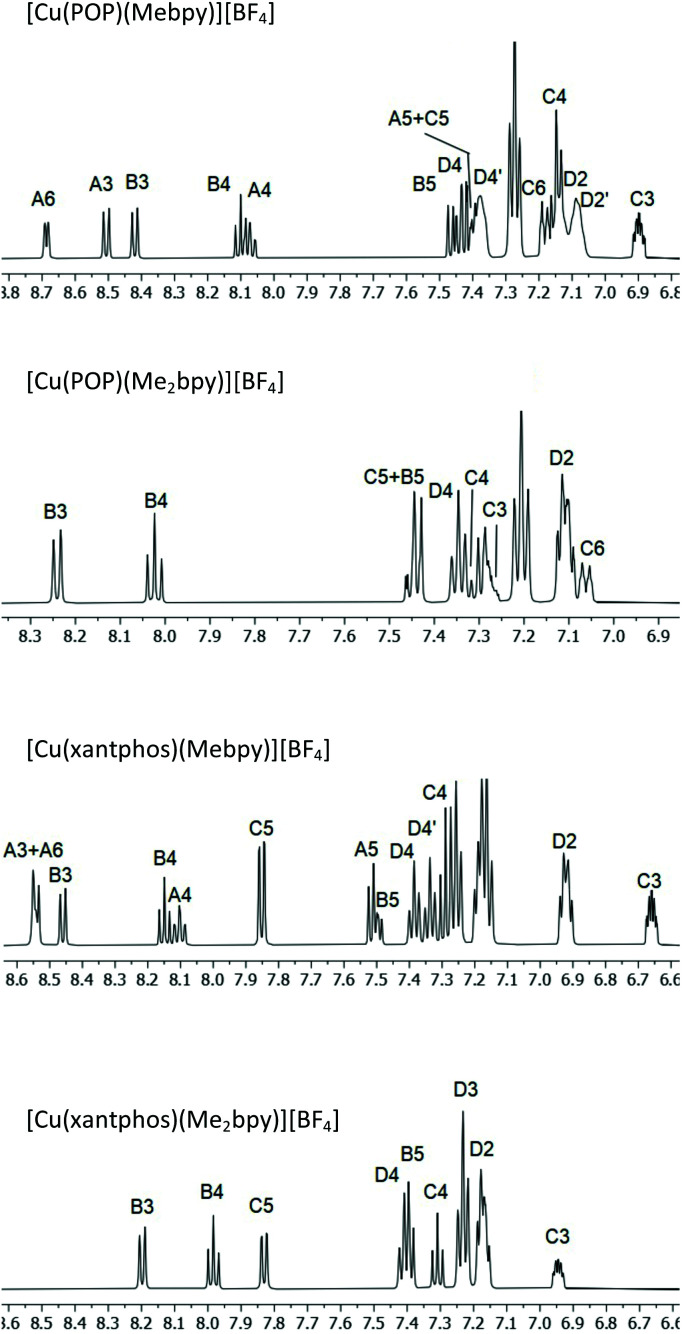
Part of the 500 MHz ^1^H NMR spectra of [Cu(POP)(Mebpy)][BF_4_], [Cu(POP)(Me_2_bpy)][BF_4_], [Cu(xantphos)(Mebpy)][BF_4_] and [Cu(xantphos)(Me_2_bpy)][BF_4_] in acetone-d_6_. Chemical shifts in *δ*/ppm. See Fig. S18–32† for the complete spectra. Atom labels are defined in Scheme 2.

^1^H, ^13^C{^1^H}, and, where appropriate, ^11^B{^1^H}, ^19^F{^1^H} and ^31^P{^1^H} NMR spectra were recorded at room temperature in acetone-*d*_6_ solutions. The ^1^H and ^13^C{^1^H} spectra were assigned using COSY, NOESY, HMQC and HMBC techniques; atom labelling used for NMR assignments are given in Scheme 2. Fig. 1 shows the aromatic regions of the ^1^H NMR spectra of [Cu(POP)(Mebpy)][BF_4_], [Cu(POP)(Me_2_bpy)][BF_4_], [Cu(xantphos)(Mebpy)][BF_4_] and [Cu(xantphos)(Me_2_bpy)][BF_4_] as representative examples (see Fig. S1–S64† for ^1^H, HMQC and HMBC NMR and ESI-MS spectra of all the complexes).

### Structural characterizations

X-ray quality single crystals of: [Cu(POP)(Mebpy)][PF_6_]·0.5CH_2_Cl_2_·0.3Et_2_O, [Cu(xantphos)(Mebpy)][PF_6_]·0.5CH_2_Cl_2_·Et_2_O, [Cu(xantphos)(Mebpy)][BF_4_]·CH_2_Cl_2_·Et_2_O and [Cu(xantphos)(Me_2_bpy)][PF_6_] were grown by slow diffusion of Et_2_O into CH_2_Cl_2_ solutions of the compounds.

Those of: [Cu(POP)(Mebpy)][BAr^F^_4_]·C_6_H_12_, [Cu(POP)(Me_2_bpy)] [BAr^F^_4_]·0.5C_6_H_12_·0.8(CH_3_)_2_CO, [Cu(xantphos)(Me_2_bpy)][BF_4_]·C_6_H_12_, [Cu(xantphos)(Me_2_bpy)][BPh_4_]·0.7C_3_H_6_O and [Cu(xantphos)(Me_2_bpy)][BAr^F^_4_] were grown by slow diffusion of cyclohexane into acetone solutions of the complexes.

**Fig. 2:**
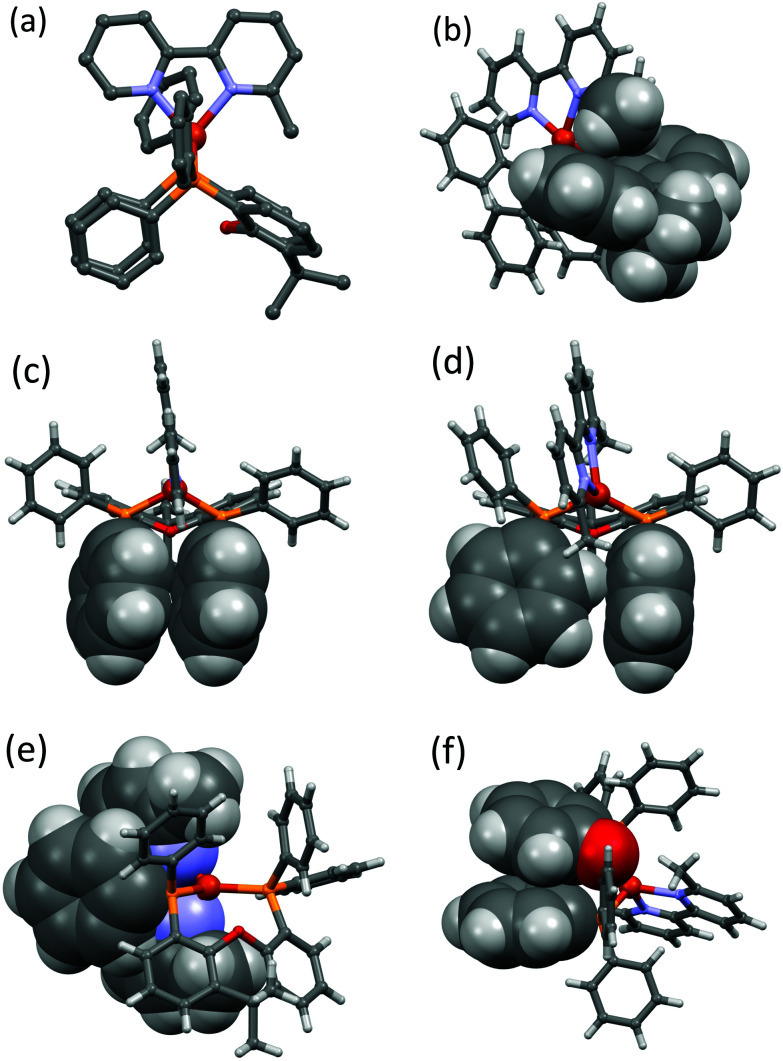
Selected structural features of the [Cu(P^P)(N^N)]^+^ cations (H-atoms omitted for clarity): (a) perspective along the P–P vector perpendicular to the Mebpy-plane in [Cu(xantphos)(Mebpy)][PF_6_]; (b) accommodation of the 6-Me group of Mebpy within the xanthene ‘cavity’ in [Cu(xantphos)(Mebpy)][PF_6_]; (c) face-to-face π-stacking of two phenyl rings connected to the two different PPh_2_ units in [Cu(xantphos)(Mebpy)][PF_6_]; (d) interaction of two phenyl rings connected to the two different PPh_2_ units in [Cu(xantphos)(Me_2_bpy)][PF_6_]; (e) offset π-stacking of one POP-phenyl ring with the Me_2_bpy ligand in [Cu(xantphos)(Mebpy)][PF_6_]; (f) face-to-face π-stacking of one POP-phenyl ring with a POP backbone ring in [Cu(POP)(Mebpy)][PF_6_].

Crystallographic data are summarized in Table S1,† and important angles and bond distances defining the copper(i) coordination sphere are summarized in Table 1 together with published data for the benchmark compounds [Cu(POP)(bpy)][PF_6_]·CHCl_3_ ^13^ and [Cu(xantphos)(bpy)][PF_6_]^55^ for comparison. The molecular structures of the complex cations are shown in Fig. S65–S74.† Most of the complexes crystallized in the triclinic space group *P*1̄ with exceptions being [Cu(POP)(Mebpy)][PF_6_]·0.5CH_2_Cl_2_·0.3Et_2_O (monoclinic *P*2_1_/*n*), [Cu(POP)(Mebpy)][BAr^F^_4_]·C_6_H_12_ (monoclinic *P*2_1_/*n*), [Cu(xantphos)(Me_2_bpy)][PF_6_] (orthorhombic *P*2_1_2_1_2_1_ with two crystallographically independent ion-pairs) and [Cu(xantphos)(Me_2_bpy)][BPh_4_]·0.7Me_2_CO (orthorhombic *Pna*2_1_ with two crystallographically independent ion-pairs). In [Cu(POP)(Mebpy)][BAr^F^_4_], the Mebpy ligand is disordered over two sites, each with 50% occupancy. The chiral space group of [Cu(xantphos)(Me_2_bpy)][PF_6_] (*P*2_1_2_1_2_1_) with a Flack parameter of 0.370(6) indicates a non-racemic mixture of the two enantiomers in the crystal lattice resulting from twinning by inversion. We have previously reported the structure of this compound (CSD Refcode GABVAJ),^58^ but in this case, it crystallized in the triclinic space group *P*1̄. The structure determinations confirm the expected bidentate chelating mode of both the bisphosphane and diimine ligands (Fig. 2a). The copper(i) centres exhibit a tetrahedral coordination geometry with varying degrees of distortion. The angles between the N–Cu–N plane and the P–Cu–P plane range from almost orthogonal (89.49°) to moderate distortion (86.18°) (Table 1).

**Table 1:** Important structural parameters in the cations in [Cu(P^P)(N^N)][A]. Benchmark [Cu(P^P)(bpy)][PF_6_] complexes are included for comparison

Complex	P–Cu–P chelating angle /°	N–Cu–N chelating angle /°	P⋯P distance/Å	Angle between PCuP and NCuN planes/°	N–C–C–N torsion angle/°
[Cu(POP)(bpy)][PF_6_]^a^	115.00(3)	79.66(7)	3.790(1)	88.5	−2.8(3)
[Cu(POP)(Mebpy)][PF_6_]^b^	112.93(3)	80.11(9)	3.773(1)	87.41	−8.0(4)
[Cu(POP)(Mebpy)][BAr^F^_4_] 1 (50%)^c^	115.43(4)	79.1(2)	3.826(1)	88.66	8.8(9)
[Cu(POP)(Mebpy)][BAr^F^_4_] 2 (50%)^c^	115.43(4)	81.4(3)	3.826(1)	89.49	11.0(9)
[Cu(POP)(Me_2_bpy)][BAr^F^_4_]	115.92(3)	80.5(1)	3.8556(8)	88.64	−2.6(4)
[Cu(xantphos)(bpy)][PF_6_]^d^	113.816(14)	79.32(5)	3.8010(5)	79.6	20.5(2)
[Cu(xantphos)(Mebpy)][PF_6_]^b^	113.44(3)	80.8(1)	3.777(1)	87.89	−1.9(5)
[Cu(xantphos)(Mebpy)][BF_4_]	113.34(3)	81.1(1)	3.778(1)	88.79	−1.0(5)
[Cu(xantphos)(Me_2_bpy)][PF_6_] 1^e^	121.53(8)	79.1(2)	4.016(3)	86.18	−10(1)
[Cu(xantphos)(Me_2_bpy)][PF_6_] 2^e^	117.77(8)	79.0(2)	3.926(3)	86.28	7(1)
[Cu(xantphos)(Me_2_bpy)][BF_4_]	111.54(3)	79.60(9)	3.777(1)	89.16	2.6(4)
[Cu(xantphos)(Me_2_bpy)][BPh_4_] 1^e^	117.99(7)	79.3(2)	3.913(2)	88.21	0.9(8)
[Cu(xantphos)(Me_2_bpy)][BPh_4_] 2^e^	113.48(7)	78.9(2)	3.841(2)	86.92	−1.0(8)
[Cu(xantphos)(Me_2_bpy)][BAr^F^_4_]	113.12(3)	79.02(9)	3.821(1)	88.88	−17.7(4)

a Data for [Cu(POP)(bpy)][PF_6_]·CHCl_3_.^13^

b Two different solvent molecules.

c Mebpy ligand is disordered over two orientations with 50% occupancy each.

d Data for [Cu(xantphos)(bpy)][PF_6_].^55^

e Two crystallographically independent ion-pairs.

In both [Cu(xantphos)(Mebpy)][PF_6_] and [Cu(xantphos)(Mebpy)][BF_4_], the 6-methyl substituent points towards the ‘bowl-shaped’ xanthene unit of the P^P ligand (Fig. 2b). The geometry of the bpy ligand is also characterized by the dihedral N–C–C–N torsional angle which ranges from a significant inter-ring torsion value of −17.7(4)° in [Cu(xantphos)(Me_2_bpy)][BAr^F^_4_] and 11.0(9)° in [Cu(POP)(Mebpy)][BAr^F^_4_] to almost coplanar pyridine rings (torsion angle = 0.9(8)° in [Cu(xantphos)(Me_2_bpy)][BPh_4_]). The P–Cu–P chelating angles vary considerably from 111.54(3)° ([Cu(xantphos)(Me_2_bpy)][BF_4_]) to 121.53(8)° ([Cu(xantphos)(Me_2_bpy)][PF_6_]). As expected, the N–Cu–N chelating angles vary little, being in a range from 79.0(2)° ([Cu(xantphos)(Me_2_bpy)][PF_6_]) to 81.4(3)° ([Cu(POP)(Mebpy)][BAr^F^_4_]). The Cu–N and Cu–P distances all lie within a typical range of 1.983(6) to 2.163(6) Å and 2.2296(8) to 2.306(2) Å, respectively.

With the exception of [Cu(xantphos)(Me_2_bpy)][PF_6_], all the xantphos-containing structures exhibit offset face-to-face π-stacking interactions between phenyl rings of two different PPh_2_ units (Fig. 2c). In [Cu(xantphos)(Mebpy)][PF_6_], the angle between the planes containing the π-stacked phenyl rings is 5.8°, the average of the two centroid⋯plane distances is 3.73 Å and the centroid⋯centroid distance is 3.84 Å. These parameters are 9.9°, 3.60 Å and 3.86 Å for [Cu(xantphos)(Mebpy)][BF_4_], 13.3°, 3.66 Å and 3.87 Å for [Cu(xantphos)(Me_2_bpy)][BF_4_], 17.3°, 4.05 Å and 4.17 Å for one of the crystallographically independent cations in [Cu(xantphos)(Me_2_bpy)][BPh_4_] and 14.36°, 3.79 Å and 3.84 Å for [Cu(xantphos)(Me_2_bpy)][BAr^F^_4_]. These all comply with the definitions delineated by Janiak.^82^ The first independent cation in [Cu(xantphos)(Me_2_bpy)][BPh_4_] exhibits two C–H⋯π contacts between one phenyl ring of each PPh_2_ group and the bpy domain (Fig. S74†) which are in agreement with Nishio.^83^ The remaining two phenyl rings engage in a π-stacking interaction with each other. The second independent cation in [Cu(xantphos)(Me_2_bpy)][BPh_4_] features a phenyl ring from one PPh_2_-unit π-stacked over the bpy domain (Fig. 2e). The angle between the least-squares planes containing the phenyl ring and the bpy is 8.6°, the average of the two centroid⋯plane distances is 3.37 Å and the centroid⋯centroid distance is 3.40 Å. The phenyl rings mentioned above, exhibiting π-stacking interactions in the first independent cation, show instead C–H⋯π – contacts in the second cation (Fig. 2d). This interaction is agreement with Nishio.^83^ The structural feature of a phenyl ring from one PPh_2_-unit π-stacked over the bpy domain (Fig. 2e) is also seen in the two independent cations in [Cu(xantphos)(Me_2_bpy)][PF_6_]. For these two cations, the angle between the least-squares planes containing the phenyl ring and the bpy-ligand is 17.56°, 18.92°, the average of the two centroid⋯plane distances is 3.52 Å, 3.51 Å and the centroid⋯centroid distance is 3.66 Å, 3.61 Å. In the initially reported structure of [Cu(xantphos)(Me_2_bpy)][PF_6_], intramolecular π-stacking between phenyl rings was observed.^58^

Two of the POP-containing structures feature a π-stacking interaction between one phenyl ring of a PPh_2_ unit and one arene ring of the POP backbone (Fig. 2f). The angle between the planes containing the π-stacked phenyl and arene rings is 18.1°, the average of the two centroid⋯plane distances is 3.73 Å and the centroid⋯centroid distance is 3.58 Å for [Cu(POP)(Mebpy)][PF_6_]. The corresponding parameters are 14.1°, 3.58 Å and 3.78 Å for [Cu(POP)(Me_2_bpy)][BAr^F^_4_].

**Fig. 3:**
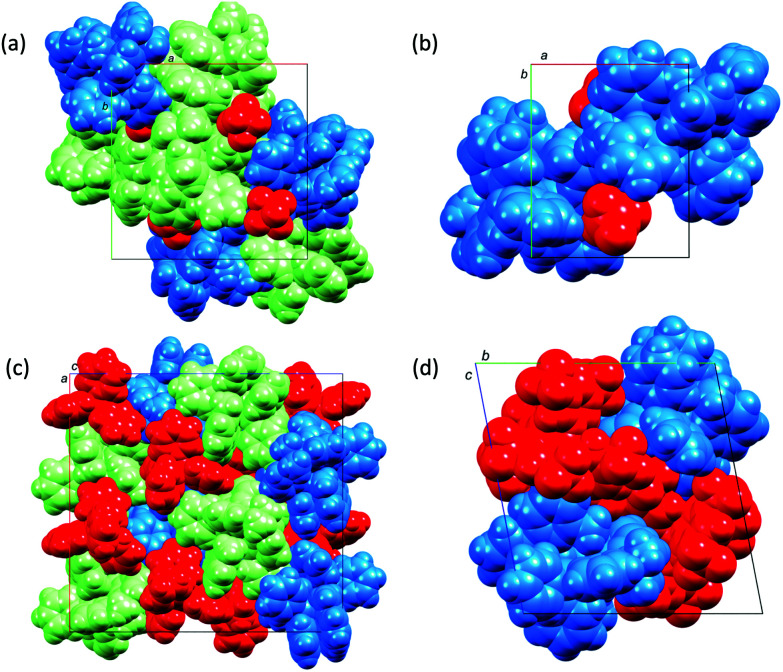
Packing of cations (blue and green) and anions (red) in (a) [Cu(xantphos)(Me_2_bpy][PF_6_] (two crystallographically independent ion-pairs), (b) [Cu(xantphos)(Me_2_bpy][BF_4_], (c) [Cu(xantphos)(Me_2_bpy][BPh_4_] (two independent ion-pairs), and (d) [Cu(xantphos)(Me_2_bpy][BAr^F^_4_]. Solvent molecules have been omitted.

**Fig. 4:**
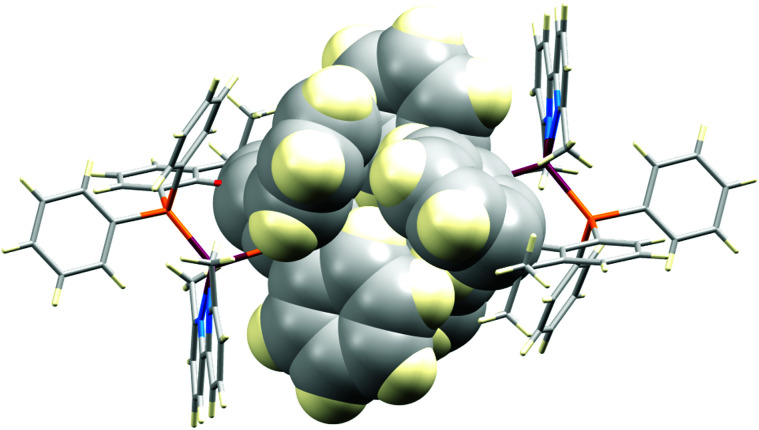
Centrosymmetric embrace of two [Cu(xantphos)(Me_2_bpy]^+^ cations in [Cu(xantphos)(Me_2_bpy][BF_4_].

The effect of altering the spatial requirements of the anion, and of introducing anions with the potential for π-stacking interactions can be assessed by considering one series in which the copper(i) cation remains constant. Fig. 3 compares the packing in the unit cells of [Cu(xantphos)(Me_2_bpy][A] where A^−^ is [PF_6_]^−^ (Fig. 3a), [BF_4_]^−^ (Fig. 3b), [BPh_4_]^−^ (Fig. 3c), and [BAr^F^_4_]^−^ (Fig. 3d). It is clear from the figure that cation⋯cation interactions are essentially switched off in [Cu(xantphos)(Me_2_bpy][BAr^F^_4_] as a consequence of the steric demands of the anions. In contrast, in [Cu(xantphos)(Me_2_bpy][PF_6_] and [Cu(xantphos)(Me_2_bpy][BF_4_], cation⋯anion interactions comprise C–H⋯F contacts, but accommodation of the [BF_4_]^−^ and [PF_6_]^−^ anions in the lattices still permits cation⋯cation interactions. In the [BF_4_]^−^ salt, pairs of cations embrace across an inversion centre with multiple edge-to-face interactions (Fig. 4). In addition, one CH unit in the phenyl ring containing C19 engages in a CH⋯π interaction with the phenyl ring containing C13^i^ (symmetry code i = −*x*, 1 − *y*, 1 − *z*; C–H⋯centroid = 2.72 Å, angle C–H⋯centroid = 147°). Finally, the Me_2_bpy C4–H4 unit forms a CH⋯π contact with the phenyl ring containing C19^ii^ (symmetry code ii = 1 + *x*, *y*, *z*; C–H⋯centroid = 2.77 Å, angle C–H⋯centroid = 132°). [Cu(xantphos)(Me_2_bpy][PF_6_] contains two crystallographically independent cations which engage in an offset face-to-face π-stacking interaction between the phenyl ring containing C40 and the pyridine ring with N3 (see Fig. S70† for atom numbers). The distance between the ring centrods is 3.79 Å and angle between the ring planes is 14.6°. There are additional inter-cation CH⋯π contacts. Adjacent CH units in the xantphos ligand containing C86 and C87 (see Fig. S70†) are directed towards the π-system of the phenyl ring with C64^i^ (symmetry code i = 1 − *x*, 1/2 + *y*, 3/2 − *z*); an analogous interaction involves xantphos units C34H34 and C35H35 and the phenyl ring containing C13^ii^ (symmetry code ii = −*x*, 1/2 + *y*, 3/2 − *z*).

### Electrochemistry

The redox properties of the complexes were investigated by cyclic voltammetry in dry propylene carbonate solution containing 0.1 mol dm^−3^ [^*n*^Bu_4_N][PF_6_] as supporting electrolyte. The cyclic voltammograms of the complexes are presented in Fig. S75–S78.† Potentials were referenced internally to ferrocene. In each cyclic voltammogram (CV), the Cu^+^/Cu^2+^ oxidation appears between *E*_pa_ = +0.81 and +0.93 V and is typically an irreversible process (see Table 2). For the two families, the Cu^+^/Cu^2+^ oxidation moves to a higher potential when going from the Mebpy to the Me_2_bpy complexes. As the copper centre is formally oxidized from Cu(i) to Cu(ii), the coordination geometry changes from tetrahedral to square planar. This can be rationalized by the two methyl substituents in Me_2_bpy preventing flattening of the Cu coordination sphere, resulting in a higher oxidation potential for the Me_2_bpy-containing compounds. For compounds containing the [Cu(xantphos)(Mebpy)]^+^ and [Cu(xantphos)(Me_2_bpy)]^+^ cations, the Cu^+^/Cu^2+^ process was partially reversible and *E*^ox^_1/2_ values are given in Table 2. In all the complexes, one partially reversible ligand-centred reduction process was observed with *E*_pc_ between −2.1 and −2.7 V. The CVs of the [BPh_4_]^−^ salts show an additional irreversible oxidation peak with *E*_pa_ between +0.48 and +0.60 V consistent with the [BPh_4_]^−^ anion undergoing an oxidation process at low potentials.

**Table 2:** Cyclic voltammetric data for [Cu(P^P)(N^N)][A] in propylene carbonate (10^−4^ to 10^−5^ mol dm^−3^, *vs.* Fc/Fc^+^, [^*n*^Bu_4_N][PF_6_] as supporting electrolyte, scan rate = 0.1 V s^−1^). When the oxidative process is reversible, both *E*^ox^_1/2_and *E*_pa_ − *E*_pc_ are given. In case of an irreversible oxidative process, *E*_pa_ is given

Complex	Oxidative process				Reductive process
	*E* ^ox^_1/2_/V	*E*_pa_ − *E*_pc_/mV	*E*_pa_/V	BPh_4_ oxidation *E*_pa_/V (irrev.)	*E* ^red^_1/2_/V
[Cu(POP)(Mebpy)][PF_6_]	—	—	+0.81	—	−2.08
[Cu(POP)(Mebpy)][BF_4_]	—	—	+0.81	—	−2.10
[Cu(POP)(Mebpy)][BPh_4_]	—	—	+0.82	+0.48	−2.13
[Cu(POP)(Mebpy)][BAr^F^_4_]	—	—	+0.82	—	−2.10
[Cu(POP)(Me_2_bpy)][PF_6_]	—	—	+0.93	—	−2.06
[Cu(POP)(Me_2_bpy)][BF_4_]	—	—	+0.93	—	−2.07
[Cu(POP)(Me_2_bpy)][BPh_4_]	—	—	+0.92	+0.60	−2.05
[Cu(POP)(Me_2_bpy)][BAr^F^_4_]	—	—	+0.92	—	−2.07
[Cu(xantphos)(Mebpy)][PF_6_]	—	—	+0.92	—	−2.11
[Cu(xantphos)(Mebpy)][BF_4_]	—	—	+0.90	—	−2.05
[Cu(xantphos)(Mebpy)][BPh_4_]	+0.85	13	+0.91	+0.46	−2.05
[Cu(xantphos)(Mebpy)][BAr^F^_4_]	—	—	+0.90	—	−2.07
[Cu(xantphos)(Me_2_bpy)][PF_6_]	—	—	+0.91	—	−2.06
[Cu(xantphos)(Me_2_bpy)][BF_4_]	+0.84	14	+0.91	—	−2.08
[Cu(xantphos)(Me_2_bpy)][BPh_4_]	+0.86	15	+0.93	+0.49	−2.07
[Cu(xantphos)(Me_2_bpy)][BAr^F^_4_]	+0.84	17	+0.92	—	−2.09

### Photophysical properties

**Fig. 5:**
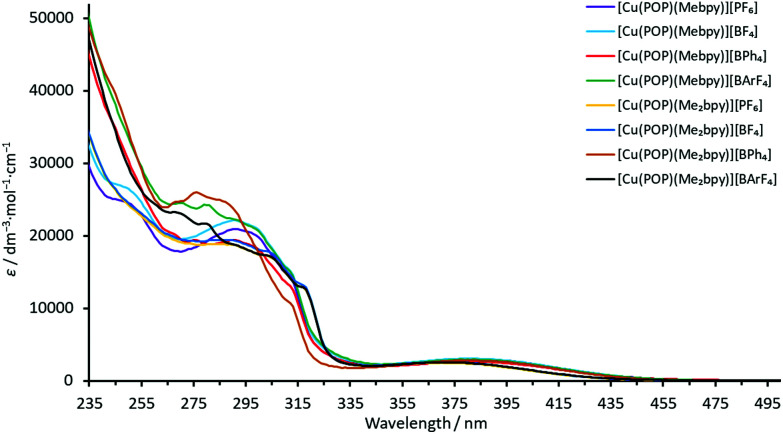
Solution absorption spectra (CH_2_Cl_2_, 2.5 × 10^−5^ mol dm^−3^) of the POP-containing heteroleptic copper(i) complexes.

**Fig. 6:**
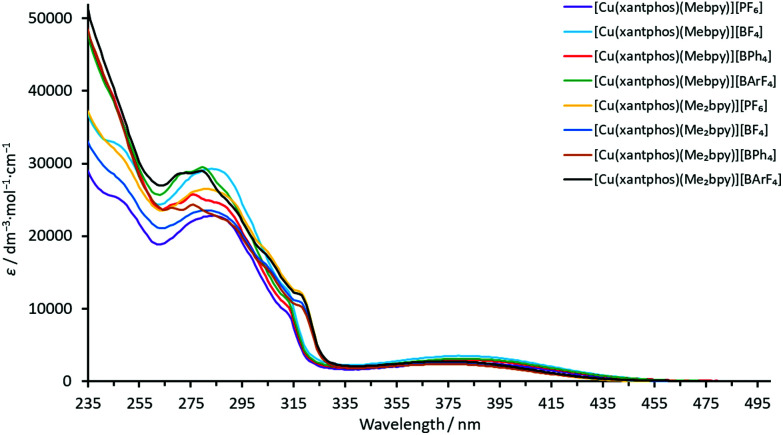
Solution absorption spectra (CH_2_Cl_2_, 2.5 × 10^−5^ mol dm^−3^) of the xantphos-containing heteroleptic copper(i) complexes.

The absorption spectra of solutions of the complexes in CH_2_Cl_2_ exhibit intense high-energy absorption bands below *ca*. 330 nm arising from ligand-centred and, in the case of the [BPh_4_]^−^ and [BAr^F^_4_]^−^ salts, counterion-centred π* ← π transitions. Additionally, each spectrum comprises a broad, lower intensity metal-to-ligand charge transfer (MLCT) band with *λ*_max_ in the range 373–385 nm for the POP-containing complexes and in the range 373–381 nm for the xantphos-containing complexes. The spectra are displayed in Fig. 5 and 6 and the absorption data are given in Table 3.

**Table 3:** Absorption maxima for CH_2_Cl_2_ solutions of [Cu(P^P)(N^N)][A]

Complex	*λ*_max_/nm (*ε*_max_/dm^3^ mol^−1^ cm^−1^)	
	π* ← π	MLCT
[Cu(POP)(Mebpy)][PF_6_]	252 sh (24 700), 292 (21 800), 301 sh (20 400), 313 sh (14 100)	385 (3300)
[Cu(POP)(Mebpy)][BF_4_]	251 sh (26 200), 292 (22 200), 302 sh (19 900), 313 sh (14 500)	383 (3100)
[Cu(POP)(Mebpy)][BPh_4_]	269 (19 700), 278 (19 000), 291 (19 300), 300 sh (18 000), 313 sh (12 500)	383 (2600)
[Cu(POP)(Mebpy)][BAr^F^_4_]	271 (24 000), 282 (23 500), 291 (21 800), 301 sh (19 900), 313 sh (14 200)	383 (3000)
[Cu(POP)(Me_2_bpy)][PF_6_]	290 (18 200), 305 (16 400), 318 sh (12 000)	374 (2410)
[Cu(POP)(Me_2_bpy)][BF_4_]	287 (18 000), 305 (16 000), 317 sh (11 500)	373 (2430)
[Cu(POP)(Me_2_bpy)][BPh_4_]	269 (26 800), 276 (25 100), 287 (23 800), 290 (23 200), 312 sh (10 300)	378 (2410)
[Cu(POP)(Me_2_bpy)][BAr^F^_4_]	269 (22 800), 280 (21 000), 292 (18 300), 305 (16 600), 317 sh (12 800)	373 (2500)
[Cu(xantphos)(Mebpy)][PF_6_]	247 sh (24 600), 275 (21 400), 285 (22 300), 289 (21 800), 313 sh (9400)	379 (2620)
[Cu(xantphos)(Mebpy)][BF_4_]	247 sh (31 800), 275 (27 800), 284 (28 600), 292 (26 300), 313 sh (11 700)	380 (3260)
[Cu(xantphos)(Mebpy)][BPh_4_]	269 (24 300), 276 (25 600), 287 (24 200), 312 sh (10 500)	380 (2820)
[Cu(xantphos)(Mebpy)][BAr^F^_4_]	271 (28 500), 281 (29 300), 288 (26 400), 312 sh (11 400)	381 (3080)
[Cu(xantphos)(Me_2_bpy)][PF_6_]	246 (31 700), 279 (26 700), 285 (26 500), 304 (18 200), 316 (12 200)	374 (2580)
[Cu(xantphos)(Me_2_bpy)][BF_4_]	248 sh (28 700), 276 (23 900), 282 (24 300), 292 (22 500), 305 sh (16 200), 318 sh (11 000)	375 (2630)
[Cu(xantphos)(Me_2_bpy)][BPh_4_]	267 (23 300), 276 (23 700), 284 (22 400), 290 (21 200), 304 sh (15 300), 319 sh (9500)	376 (2200)
[Cu(xantphos)(Me_2_bpy)][BAr^F^_4_]	271 (28 100), 280 (28 400), 292 sh (23 400), 304 sh (17 300), 318 sh (11 600)	374 (2680)

The MLCT absorption of the Me_2_bpy-containing complexes is shifted to higher energies compared to the analogous Mebpy-containing compounds (Table 3). This is consistent with the electron-donating methyl groups destabilizing the LUMO to a greater extent in the Me_2_bpy – compared to the Mebpy containing compounds, the LUMO being mainly located on the N^N ligand.

**Fig. 7:**
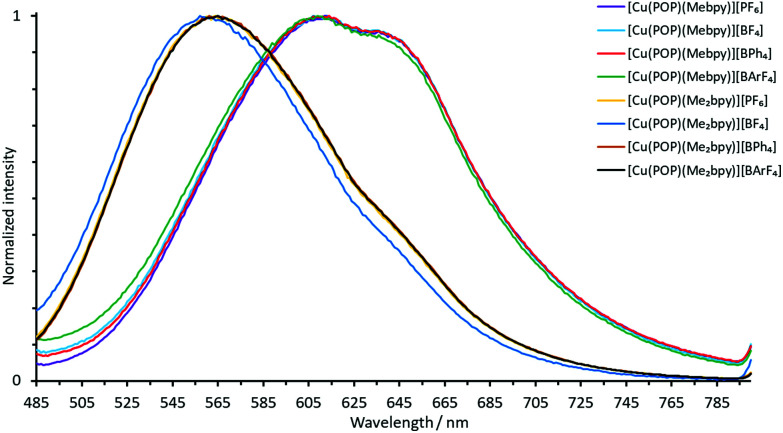
Normalized solution emission spectra of the POP-containing heteroleptic copper(i) complexes (deaerated CH_2_Cl_2_, 1.0 × 10^−5^ mol dm^−3^, *λ*_exc_ = 365 nm).

**Fig. 8:**
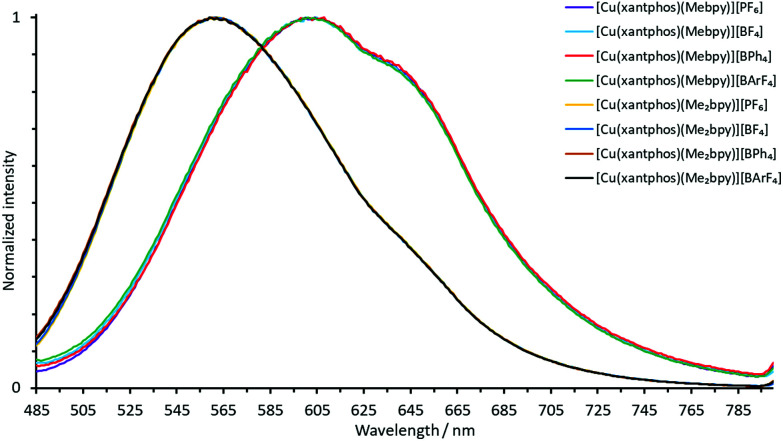
Normalized solution emission spectra of the xantphos-containing heteroleptic copper(i) complexes (deaerated CH_2_Cl_2_, 1.0 × 10^−5^ mol dm^−3^, *λ*_exc_ = 365 nm).

**Fig. 9:**
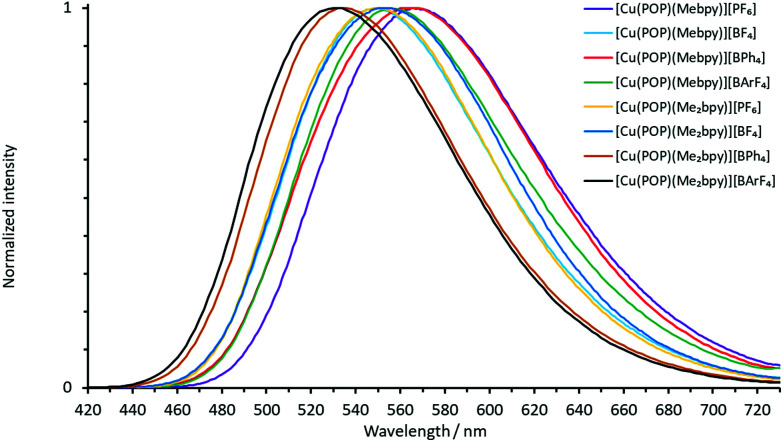
Normalized emission spectra of powdered samples of the POP-containing heteroleptic copper(i) complexes (*λ*_exc_ = 365 nm).

**Fig. 10:**
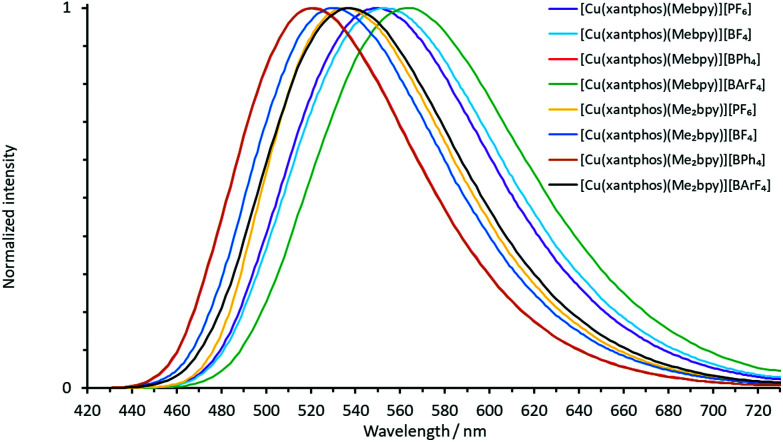
Normalized emission spectra of powdered samples of the xantphos-containing heteroleptic copper(i) complexes (*λ*_exc_ = 365 nm).

The normalized solution emission spectra of the complexes in deaerated CH_2_Cl_2_ solution with excitation wavelengths in the region of their MLCT band are displayed in Fig. 7 and 8. The solid-state (powder) emission spectra of the complexes are shown in Fig. 9 and 10, and photophysical data are summarized in Table 4. Solution emission spectra were measured with an excitation wavelength of *λ*_exc_ = 410 nm to avoid overlapping of the second harmonic of the excitation peak with the broad emission band. Excitation at *λ*_exc_ = 365 nm resulted in an identical emission band after normalization.

**Table 4:** Photophysical properties of the [Cu(P^P)(N^N)][A] complexes

Complex	Solution (CH_2_Cl_2_, de-aerated, 1.0 × 10^−5^ mol dm^−3^)				Powder					
	*λ*_exc_/nm	*λ* ^em^_max_/nm	PLQY/%	*τ*/μs	*λ*_exc_/nm	*λ*_max_^em^/nm	PLQY/%	*τ* a/μs	*τ*(1)/μs (*A*_1_)	*τ*(2)/μs (*A*_2_)
[Cu(POP)(Mebpy)][PF_6_]	410	609, 637	1.1	0.37	365	565	12	2.9	0.6 (0.070)	3.1 (0.89)
[Cu(POP)(Mebpy)][BF_4_]	410	609, 637	1.2	0.37	365	549	21	8.0	2.8 (0.19)	9.4 (0.77)
[Cu(POP)(Mebpy)][BPh_4_]	410	609, 637	0.9	0.39	365	563	10	4.9	2.1 (0.34)	6.5 (0.57)
[Cu(POP)(Mebpy)][BAr^F^_4_]	410	609, 637	1.5	0.42	365	555	6.6	3.3	1.7 (0.41)	4.7 (0.47)
[Cu(POP)(Me_2_bpy)][PF_6_]	410	566, 620	13	4.5	365	549	34	8.7	2.5 (0.14)	9.8 (0.81)
[Cu(POP)(Me_2_bpy)][BF_4_]	410	560, 616	12	4.1	365	553	28	8.7	2.6 (0.14)	9.7 (0.82)
[Cu(POP)(Me_2_bpy)][BPh_4_]	410	566, 620	13	4.2	365	533	24	10.0	11.0 (0.84)	2.1 (0.11)
[Cu(POP)(Me_2_bpy)][BAr^F^_4_]	410	566, 620	14	4.5	365	532	24	8.4	3.0 (0.34)	11.4 (0.60)
[Cu(xantphos)(Mebpy)][PF_6_]	410	603, 636	1.3	0.72	365	550	33	10.5	11.2 (0.91)	1.6 (0.067)
[Cu(xantphos)(Mebpy)][BF_4_]	410	603, 636	1.3	0.82	365	552	20	7.5	2.0 (0.23)	9.3 (0.69)
[Cu(xantphos)(Mebpy)][BPh_4_]	410	603, 636	1.4	0.77	365	520	13	12.7	13.8 (0.87)	1.7 (0.095)
[Cu(xantphos)(Mebpy)][BAr^F^_4_]	410	603, 636	1.5	0.83	365	562	13	5.2	1.9 (0.10)	5.6 (0.86)
[Cu(xantphos)(Me_2_bpy)][PF_6_]	410	563, 631	8.3	3.3	365	535	62	14.7	15.1 (0.93)	0.99 (0.020)
[Cu(xantphos)(Me_2_bpy)][BF_4_]	410	563, 631	9.1	3.1	365	530	44	8.7	1.6 (0.34)	13.1 (0.57)
[Cu(xantphos)(Me_2_bpy)][BPh_4_]	410	563, 631	8.2	3.0	365	520	35	12.9	14.0 (0.88)	2.0 (0.083)
[Cu(xantphos)(Me_2_bpy)][BAr^F^_4_]	410	563, 631	8.3	3.7	365	536	27	8.5	3.1 (0.30)	11.1 (0.64)

a A biexponential fit to the lifetime delay was used because a single exponential gave a poor fit; *τ* is calculated from the equation ∑*A*_*i*_*τ*_*i*_/∑(*A*_*i*_) and *A*_*i*_ is the pre-exponential factor for the lifetime and values of *τ*(1), *τ*(2), *A*_1_ and *A*_2_ are also given. Deaeration was performed by bubbling a stream of argon through the solution.

The solid-state emission maxima of the complexes lie between 520 and 565 nm and thus, the complexes are green to yellow emitters in powdered form. In solution, the emission maxima are red-shifted to a range between 560 and 636 nm which gives yellow to orange emission. This red-shift corresponds to previous observations for similar families of complexes.^58^ Upon changing from complexes containing Mebpy to Me_2_bpy for a given P^P ligand, the solution emission maxima are significantly blue-shifted in the range of 55–60 nm, caused by the increased steric stabilization of the coordination sphere. The previously reported solution emission maxima of *λ*^max^_em_ = 618, 649 nm for [Cu(POP)(bpy)][PF_6_] and *λ*^max^_em_ = 620, 650 nm for [Cu(xantphos)(bpy)][PF_6_] are consistent with this.^55^

In the solid-state emission spectra, the highest energy emission maxima are for [Cu(xantphos)(Mebpy)][BPh_4_] and [Cu(xantphos)(Me_2_bpy)][BPh_4_] (*λ*^em^_max_ = 520 nm) whereas the lowest energy emission maxima are for [Cu(POP)(Mebpy)][PF_6_] and [Cu(POP)(Mebpy)][BPh_4_] with *λ*^em^_max_ = 565 nm and 563 nm, respectively. Upon going from complexes containing Mebpy to Me_2_bpy for a given P^P ligand, the solid-state emission maxima undergo a blue-shift of 15–30 nm.

**Fig. 11:**
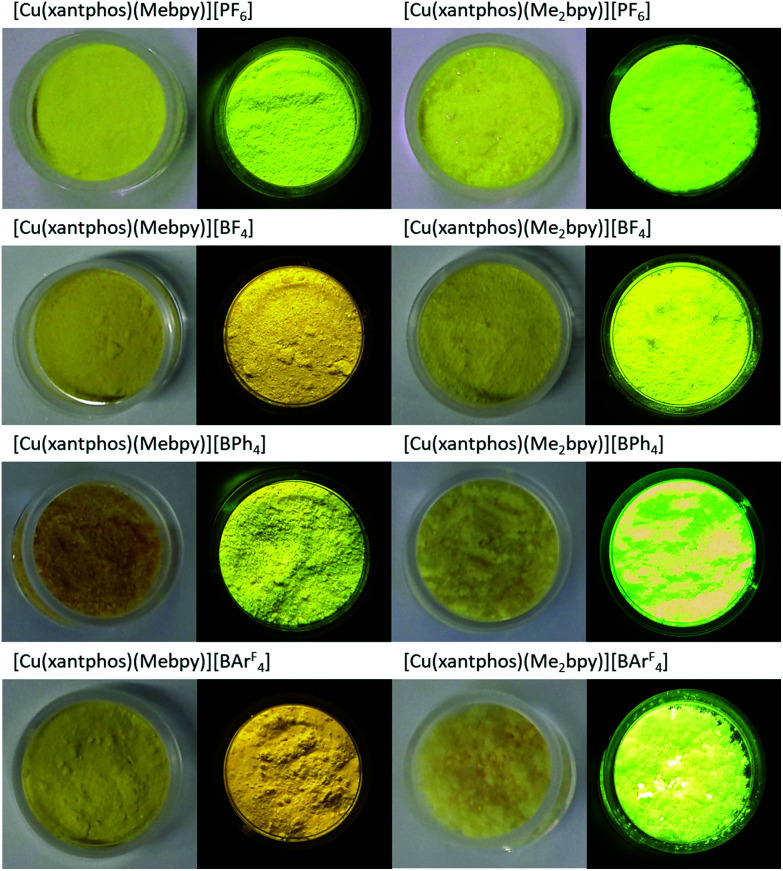
Powder samples of [Cu(xantphos)(N^N)][A] complexes under ambient light (left) and under UV light (*λ*_exc_ = 366 nm, right).

The appearance and luminescence of powdered samples of the [Cu(xantphos)(N^N)][A] and [Cu(POP)(N^N)][A] complexes are illustrated in Fig. 11 and Fig. S79,† respectively, with samples shown under daylight and under UV irradiation (*λ*_exc_ = 366 nm).

The emissive behaviour of the complexes is enhanced in the solid-state relative to deaerated solution. The solid-state PLQY values lie within the range of 10–62%, compared to solution values of 0.9–14% (Table 4). Salts of [Cu(POP)(Me_2_bpy)]^+^ and [Cu(xantphos)(Me_2_bpy)]^+^ have the highest PLQYs both in the solid state and solution which can be ascribed to the increased steric hindrance in the Cu(i) coordination sphere provided by the Me_2_bpy ligand. This impedes flattening of the tetrahedron upon excitation. This is consistent with the solid-state PLQY-values reported for the unsubstituted bpy containing complexes of 3.0% for [Cu(POP)(bpy)][PF_6_] and 1.7% for [Cu(xantphos)(bpy)][PF_6_].^55^ Sterically protected copper centres are less accessible to, for example, solvent molecules. Exciton quenching by non-radiative intermolecular processes like collisional quenching, Förster resonance energy transfer^84^ and Dexter electron transfer^85^ are also expected to be reduced. In solution, salts of [Cu(POP)(Me_2_bpy)]^+^ have the highest PLQYs (Table 4, average 13%). In contrast, in the solid state, salts of [Cu(xantphos)(Me_2_bpy)]^+^ show the highest PLQY values with a range of 27–62% (Table 4). The solid-state PLQY of [Cu(POP)(Mebpy)][PF_6_] of 12% is higher than the value we have previously reported (9.5%).^21^ On the other hand, for [Cu(POP)(Me_2_bpy)][PF_6_], a lower solid-state PLQY of 34% was measured compared to the reported 38%.^21^ There is also some variation when comparing the measured PLQY values of 62% for [Cu(xantphos)(Me_2_bpy)][PF_6_] to the reported value of 37%, respectively.^58^ The PLQYs in this work were recorded on the same instrument as the previously reported data and the most likely reason is the different morphology of the samples. Linfoot *et al.* have previously observed a similar phenomenon.^86^ Both in solution and in solid-state, the *λ*^em^_max_ of [Cu(POP)(Mebpy)][PF_6_] and [Cu(POP)(Me_2_bpy)][PF_6_] are very close to the data reported by Keller *et al.*^21^ In terms of the solid-state structures, two factors have been identified in the literature that may contribute to the solid-state PLQY. The first is the non-bonded Cu⋯O distance, the O atom being in the POP or xantphos ligand,^87^ and the second is the intra-cation π-stacking.^88^ In the series of compounds described in this paper, it is difficult to see clear correlations between these structural factors and the solid-state PLQY values. It is also complicated by the introduction of aromatic groups in the [BPh_4_]^−^ and [BAr^F^_4_]^−^ anions.

The excited state lifetimes *τ* of the solid-state samples were determined using a biexponential fit;^89^ the data for both solution and solid state are displayed in Table 4. The excited state lifetimes of the powder samples range from 2.9 μs for [Cu(POP)(Mebpy)][PF_6_] to 14.7 μs for [Cu(xantphos)(Me_2_bpy)][PF_6_]. Between solutions of all salts, both Me_2_bpy containing cations consistently exhibit increased excited state lifetimes compared to the Mebpy containing cations.

### Device properties

We have previously reported the performances of LECs containing [Cu(POP)(Mebpy)][PF_6_], [Cu(POP)(Me_2_bpy)][PF_6_], [Cu(xantphos)(Mebpy)][PF_6_] and [Cu(xantphos)(Me_2_bpy)][PF_6_] in their active layers, but under different device driving conditions.^21,58^ These compounds exhibit some of the highest PLQY values of known [Cu(P^P)(N^N)]^+^ complexes and are, therefore, good candidates for LECs. In this work, we chose to focus on the series [Cu(xantphos)(Me_2_bpy)][PF_6_], [Cu(xantphos)(Me_2_bpy)][BF_4_], [Cu(xantphos)(Me_2_bpy)][BPh_4_] and [Cu(xantphos)(Me_2_bpy)][BAr^F^_4_] to study their electroluminescence properties when used as active layers in LECs.

**Fig. 12:**
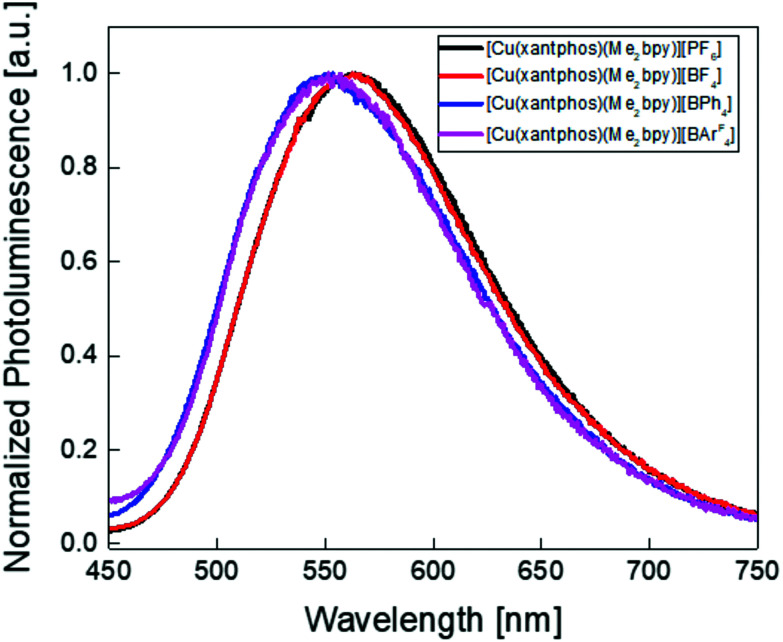
Normalized thin-film photoluminescence spectra of the [Cu(xantphos)(Me_2_bpy)]^+^ complexes with different counterions.

**Fig. 13:**
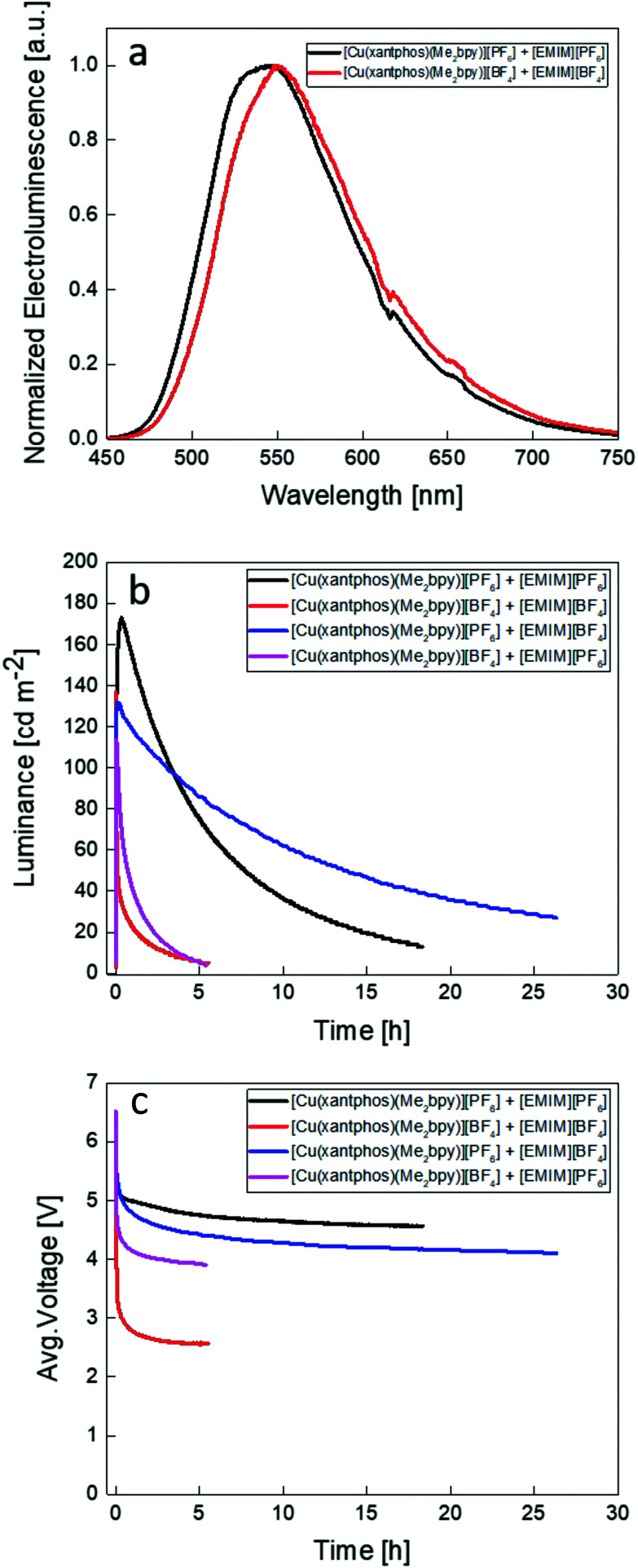
(a) Normalized electroluminescence spectra of the two best performing complexes [Cu(xantphos)(Me_2_bpy)][PF_6_] and [Cu(xantphos)(Me_2_bpy)][BF_4_]. (b) Luminance values and (c) voltage of [Cu(xantphos)(Me_2_bpy)][PF_6_] and [Cu(xantphos)(Me_2_bpy)][BF_4_] LECs driven at an average current density of 50 A m^−2^ with different IL counterions.

The thin-film PL spectra and PLQY of complexes were measured and are shown in Fig. 12 and Table 5. The PL spectra reveal that the four complexes do not have exactly the same PL maximum, consistent with the solution and powder PL spectra. Both [Cu(xantphos)(Me_2_bpy)][PF_6_] and [Cu(xantphos)(Me_2_bpy)][BF_4_] show a PL maximum at 563 nm whereas thin-films of [Cu(xantphos)(Me_2_bpy)][BPh_4_] and [Cu(xantphos)(Me_2_bpy)][BAr^F^_4_] have values of *λ*^em^_max_ = 548 and 552 nm, respectively. This is possibly associated with the different cation⋯anion interactions discussed earlier (Fig. 3). The PLQYs of the thin films are 44, 45, 32 and 35%, respectively, for the [PF_6_]^−^, [BF_4_]^−^, [BPh_4_]^−^ and [BAr^F^_4_]^−^ salts. The EL spectra of the LECs using the best performing complexes, [Cu(xantphos)(Me_2_bpy)][PF_6_] and [Cu(xantphos)(Me_2_bpy)][BF_4_], were also measured with values of *λ*^em^_max_(EL) of 546 and 550 nm, respectively (Fig. 13a). The EL is blue-shifted with respect to the PL in solution and red-shifted with respect to the PL in solid state.^20^Table 6 displays the active layers of the devices and LEC main figures of merit. As described in the Experimental section, the complexes were mixed with ILs (4 : 1 molar ratio complex : IL) containing the same and different counterions in order to study the behaviour of these complexes in LEC devices and the specific effect of the IL anion on the performance of the device. The cells were then driven under an average pulsed current of 50 A m^−2^ while monitoring the luminance and voltage behaviour. Device performances of LECs containing [Cu(xantphos)(Me_2_bpy)][PF_6_] and [Cu(xantphos)(Me_2_bpy)][BF_4_] mixed with [EMIM]^+^ ILs using the same counterion as the copper(i) complex can be seen in Fig. 13b (black and red curves, respectively). In both cases the cells have the typical LEC behaviour characterized by an initial high resistance and hence, a high initial voltage. As the electrochemical doping takes place over time, the film conductivity increases, and the voltage drops. The luminance increases following the electrochemical doping until a maximum value is reached. Then, a rapid loss of the EL intensity is observed, probably due to quenching caused by the growing doped zones as the voltage maintains a steady value, where smaller anions result in lower steady state voltage (Fig. 13c). Both devices show a fast turn-on time of 58 s and 15 s (the time to reach a luminance of 100 cd m^−2^) with a maximum luminance of 173 cd m^−2^ and 137 cd m^−2^ respectively. As expected from the anion sizes, [Cu(xantphos)(Me_2_bpy)][BF_4_] has a faster turn-on time, as the [BF_4_]^−^ ion has smaller radius than [PF_6_]^−^, and thus is expected to have a higher mobility in the device. The LECs have a maximum current efficiency (CE) of 3.5 cd A^−1^ and 2.7 cd A^−1^, respectively (Fig. 80†). Recent works explored the electroluminescent properties of copper complexes with similar P^P and N^N ligands: [Cu(xantphos)(4,5,6-Me_3_bpy)][PF_6_],^20^ [Cu(xantphos)(Mebpy)][PF_6_]^58^ and [Cu(BnN-xantphos)(Me_2_bpy)][PF_6_].^90^ When comparing the performances of these complexes with the LECs in the current investigation (Table 6) we notice similar luminance and current efficiencies (CE) with values of 190 cd m^−2^ and 3.8 cd A^−1^, 90 cd m^−2^ and 1.9 cd A^−1^ and 179 cd m^−2^ and 3.6 cd A^−1^, respectively, when operated under the same driving conditions. It is important to notice that the added IL was not always the same in all studies, and this can affect the performance of the device, as we show here. Additionally, the reported [Cu(xantphos)(Me_2_bpy)][PF_6_] was also previously studied^58^ mixed with the IL [1-butyl-3-methylimidazolium][PF_6_] ([BMIM][PF_6_]). The devices show a slightly lower luminance of 145 cd m^−2^ and CE of 3.0 cd A^−1^. The performances of devices using [Cu(xantphos)(Me_2_bpy)][PF_6_] and [Cu(xantphos)(Me_2_bpy)][BF_4_] with mixed counterions ([EMIM][BF_4_] and [EMIM][PF_6_]) can be seen in Fig. 13b (blue and purple curves, respectively) and in Table 5. The addition of the IL with a different counterion seems to affect the luminance and the turn-on time of the LEC. Both the luminance of LECs containing [Cu(xantphos)(Me_2_bpy)][BF_4_] and [Cu(xantphos)(Me_2_bpy)][PF_6_] decrease to 132 cd m^−2^ and 114 cd m^−2^ at a maximum CE of 2.6 cd A^−1^ and 2.3 cd A^−1^, respectively. The turn-on time (time to reach 100 cd m^−2^) increases to 47 s for [Cu(xantphos)(Me_2_bpy)][BF_4_], while [Cu(xantphos)(Me_2_bpy)][PF_6_] shows a similar turn-on time of 61 s (Table 5).

**Table 5:** Photoluminescence properties of thin films of the [Cu(xantphos)(Me_2_bpy)]^+^ complexes with different counterions

Compound	*λ* ^em^_max_/nm (*λ*_exc_ = 365 nm)	PLQY/%
[Cu(xantphos)(Me_2_bpy)][PF_6_]	563	44
[Cu(xantphos)(Me_2_bpy)][BF_4_]	563	45
[Cu(xantphos)(Me_2_bpy)][BPh_4_]	548	32
[Cu(xantphos)(Me_2_bpy)][BAr^F^_4_]	552	35

**Table 6:** Performance of LECs with the [Cu(xantphos)(Me_2_bpy)]^+^ series in the active layer; cell architecture ITO/PEDOT:PSS/[Cu(xantphos)(Me_2_bpy)][A] : [EMIM][A] (4 : 1 molar ratio)/Al. LECs were measured using a pulsed current driving (average current density 50 A m^−2^, 1 kHz, 50% duty cycle, block wave)

Complex	Max luminance/cd m^−2^	Max current efficiency/cd A^−1^	Turn-on time^a^/s
[Cu(xantphos)(Me_2_bpy)][PF_6_] + [EMIM][PF_6_]	173	3.5	58
[Cu(xantphos)(Me_2_bpy)][BF_4_] + [EMIM][BF_4_]	137	2.7	15
[Cu(xantphos)(Me_2_bpy)][PF_6_] + [EMIM][BF_4_]	132	2.6	61
[Cu(xantphos)(Me_2_bpy)][BF_4_] + [EMIM][PF_6_]	114	2.3	47

a Turn-on-time is time to time to reach a luminance of 100 cd m^−2^.

The two complexes with larger aryl-substituted anions, [Cu(xantphos)(Me_2_bpy)][BPh_4_] and [Cu(xantphos)(Me_2_bpy)][BAr^F^_4_], were also used in LECs. As before, two ILs with different anions were employed: one in which the anion in the IL ([EMIM][BPh_4_] and [EMIM][BAr^F^_4_]) matched the anion in the complex, and another in which the anion in the IL is [PF_6_]^−^ (IL = [EMIM][PF_6_]). In both cases the devices maintained a high voltage value of 9 V (limit of our setup) and did not turn-on after several minutes even at higher driving current density (avg. 100 A m^−2^ and 200 A m^−2^) and with higher IL concentrations (2 : 1, Cu : IL). The failure to turn on is an indication of low charge injection/transport efficiency within the thin film. In the case of large counterions, the charge injection might be less efficient due to the lower ionic mobility. These results indicate that mixing counterions is not likely to produce a beneficial change in performance, since it reduces all the figures of merit in LECs, as shown for [Cu(xantphos)(Me_2_bpy)][PF_6_] and [Cu(xantphos)(Me_2_bpy)][BF_4_] (Table 5). Moreover, using ILs with smaller counterions (*e.g.* [PF_6_]^−^) for devices using big aryl-substituted complexes, such as [Cu(xantphos)(Me_2_bpy)][BPh_4_] and [Cu(xantphos)(Me_2_bpy)][BAr^F^_4_] is not sufficient to turn on these LECs.

## Conclusions

We have described the syntheses of [Cu(POP)(Mebpy)][A], [Cu(POP)(Me_2_bpy)][A], [Cu(xantphos)(Mebpy)][A] and [Cu(xantphos)(Me_2_bpy)][A] in which [A]^−^ is [BF_4_]^−^, [PF_6_]^−^, [BPh_4_]^−^ or [BAr^F^_4_]^−^. The [PF_6_]^−^ salts have previously been described,^21,58^ but are reported here for comparative purposes. Nine [Cu(P^P)(N^N)][A] salts were characterised by single crystal X-ray crystallography. As expected, a change from [BF_4_]^−^ or [PF_6_]^−^ to the more sterically demanding [BPh_4_]^−^ or [BAr^F^_4_]^−^ counterions has a significant impact on the packing interactions in the solid state. Cation⋯cation interactions are effectively switched off in [Cu(xantphos)(Me_2_bpy][BAr^F^_4_] as a result of the steric demands of the anions. In contrast, in [Cu(xantphos)(Me_2_bpy][PF_6_] and [Cu(xantphos)(Me_2_bpy][BF_4_], there are extensive C–H⋯F contacts between cations and anions, but accommodation of the [BF_4_]^−^ and [PF_6_]^−^ anions in the lattices still allows cation⋯cation interactions.

We reported the effects of the counterion on the photophysical properties of [Cu(POP)(N^N)][A] and [Cu(xantphos)(N^N)][A] (N^N = Mebpy and Me_2_bpy). While a change from Mebpy to Me_2_bpy has previosuly been explored,^21,58^ the current investigation revealed an anion dependence on *λ*^em^_max_ and PLQY. In the solid-state emission spectra, the highest energy *λ*^em^_max_ values are for [Cu(xantphos)(Mebpy)][BPh_4_] and [Cu(xantphos)(Me_2_bpy)][BPh_4_] (*λ*^em^_max_ = 520 nm) whereas the lowest energy *λ*^em^_max_ values occur for [Cu(POP)(Mebpy)][PF_6_] and [Cu(POP)(Mebpy)][BPh_4_] (565 nm and 563 nm, respectively). Variation in PLQY is illustrated for the [Cu(xantphos)(Me_2_bpy)][A] series, in which PLQYs decrease from 62% for [PF_6_]^−^, to 44%, 35% and 27% for [BF_4_]^−^, [BPh_4_]^−^ and [BAr^F^_4_]^−^, respectively. The [Cu(xantphos)(Me_2_bpy)][A] compounds were incorporated into the active layers of LECs. The luminophores were mixed with [EMIM][A] ILs in which [A]^−^ was the same or a different counterion than in the copper(i) complex. LECs containing [Cu(xantphos)(Me_2_bpy)][BPh_4_] and [Cu(xantphos)(Me_2_bpy)][BAr^F^_4_] failed to turn on under the LEC operating conditions, whereas those with the smaller [PF_6_]^−^ or [BF_4_]^−^ counterions had rapid turn-on times and exhibited maximum luminances of 173 or 137 cd m^−2^ and current efficiencies of 3.5 and 2.6 cd A^−1^, respectively, if the IL contained the same counterion as the luminophore. Mixing the counterions ([PF_6_]^−^ and [BF_4_]^−^) in the active complex and the IL led to a reduction in all the figures of merit of the LECs.

## Conflicts of interest

There are no conflicts to declare.

## Supplementary Material

DT-050-D1DT03239A-s001

DT-050-D1DT03239A-s002
