# Supplementary material for: A counterion study of a series of [Cu(P^P)(N^N)][A] compounds with bis(phosphane) and 6-methyl and 6,6′-dimethyl-substituted 2,2′-bipyridine ligands for light-emitting electrochemical cells
Source: Dalton Trans. 2021 Nov 2;50(48):17920–34. doi: 10.1039/d1dt03239a (PMC8669729; doi:10.1039/d1dt03239a)
Supplement: DT-050-D1DT03239A-s001 [file DT-050-D1DT03239A-s001.pdf]

Supporting Information:

A counterion study of a series of [Cu(P^P)(N^N)][A] compounds with bis(phosphane) and 6-methyl and 6,6'-dimethyl-substituted 2,2'-bipyridine ligands for light-emitting electrochemical cells

Marco Meyer,[a] Lorenzo Mardegan,[b] Daniel Tordera,[b] Alessandro Prescimone,[a] Michele Sessolo,*[b] Edwin C. Constable,[b] and Catherine E. Housecroft*[a]

Contents

Experimental and analytical data

Figs. S1–S64	^{1}H, HMQC and HMBC NMR spectra and mass spectra of the copper(I) complexes
Table S1	Crystallographic data
Figs. S65–S74	Structural figures
Figs. S75–S78	Cyclic voltammograms
Table S2	PL properties of thin films and electroluminescence maxima for LECs
Fig. S79	Photography of powdered samples of [Cu(POP)(N^N)][A] complexes
Fig. S80	Current efficiency of the best working LECs

Experimental and analytical data

General Procedures for $[PF_6]^-$ and $[BF_4]^-$ salts of the copper(I) complexes

POP-containing compounds were synthesized by the following procedure: POP (1.1 eq.) and $[Cu(MeCN)_4][PF_6]$ or $[Cu(MeCN)_4][BF_4]$ (1.0 eq.) were dissolved in CH_2Cl_2 (20 mL) and the reaction mixture was stirred for 1.5 h. The respective N^N ligand (1.0 eq.) was added, followed by stirring the mixture for 2 h. The solvent was then removed under reduced pressure. The residue was purified by precipitation from CH_2Cl_2 with Et_2O, followed by centrifugation and the decantation of the supernatant. This step was repeated four times. Then the product was washed with cyclohexane (100 mL).

Compounds containing xantphos were prepared according to the following procedure: A solution of the respective N^N-ligand (1.0 eq.) and xantphos (1.1 eq.) in CH_2Cl_2 (10 mL) was added dropwise to a CH_2Cl_2 solution (10 mL) of $[Cu(MeCN)_4][PF_6]$ or $[Cu(MeCN)_4][BF_4]$ (1.0 eq.). The reaction mixture was then stirred for 2 h before the solvent was removed under reduced pressure. The residue was purified by precipitation from CH_2Cl_2 with Et_2O, followed by centrifugation and the decantation of the supernatant. This step was repeated four times. Then the product was washed with cyclohexane (100 mL) and dried under high vacuum.

General Procedures for $[BPh_4]^-$ and $[BAr^F{}_4]^-$ salts of the copper(I) complexes

The following procedure was adapted from a literature method[1]. To synthesise the $[BPh_4]^-$ and $[BAr^F{}_4]^-$ salts, an ion exchange was carried out starting with the respective $[PF_6]^-$ salt.

The $[PF_6]^-$ salt (1.0 eq.) of the desired complex was dissolved in a minimal amount of MeOH at 45 °C while sonicating. Then, $NaBPh_4$ (1.3 eq.) or $NaBAr^F{}_4$ (1.3 eq.), respectively, was added into the warm solution. The mixture was sonicated and H_2O (60 mL) was added to precipitate the product. The product was then washed with H_2O and dried under vacuum. For the $[BPh_4]^-$ salts, the crude product was dissolved in a minimal amount of $MeOH/CH_2Cl_2$ (3:1) while sonicating, followed by another addition of $NaBPh_4$ (1.3 eq.). The mixture was sonicated and H_2O (60 mL) was added to precipitate the product. The product was then washed with H_2O and dried under vacuum. The products were purified as follows. The crude product was dissolved in CH_2Cl_2 (20 mL) and water was added (15 mL). The mixture was vigorously shaken, centrifuged and the aqueous phase was removed. The organic phase was dried with $MgSO_4$. The product was precipitated from CH_2Cl_2 with Et_2O, followed by centrifugation and the decantation of the

Please do not adjust margins

supernatant. This step was repeated three times. Then the product was washed with cyclohexane (100 mL) and dried under high vacuum.

[Cu(POP)(Mebpy)][PF$_6$]

The reagents were POP (137 mg, 0.252 mmol), Mebpy (42.6 mg, 0.250 mmol) and [Cu(MeCN)$_4$][PF$_6$] (93.3 mg, 0.250 mmol). [Cu(POP)(Mebpy)][PF$_6$] was isolated as a yellow solid (165 mg, 0.180 mmol, 72%).
^{1}H NMR (500 MHz, acetone-d$_6$) δ/ppm 8.68 (d, $^2J_{HH}$ = 4.7 Hz, 1H, H^{A6}), 8.50 (d, $^2J_{HH}$ = 8.1 Hz, 1H, H^{A3}), 8.41 (d, $^2J_{HH}$ = 7.9 Hz, 1H, H^{B3}), 8.10 (m, 1H, H^{B4}), 8.08 (m, 1H, H^{A4}), 7.47 (d, $^2J_{HH}$ = 7.7 Hz, 1H, H^{B5}), 7.43 (m, 2H, H^{D4}), 7.41 (m, 1H, H^{A5}), 7.41 (m, 2H, H^{C5}), 7.38 (m, 2H, H$^{D4'}$), 7.27 (m, 4H, H^{D3}), 7.27 (m, 4H, H$^{D3'}$), 7.19 (m, 2H, H^{C6}), 7.15 (m, 2H, H^{C4}), 7.14 (m, 4H, H^{D2}), 7.10 (m, 4H, D2'), 6.90 (m, 2H, H^{C3}), 2.35 (s, 3H, H$^{B6\text{-}Me}$).
^{13}C{^{1}H} NMR (126 MHz, acetone-d$_6$) δ/ppm 160.0 (s, C^{B6}), 158.9 (pseudo-t, 2J$_{CP}$ = 6.0 Hz, C^{C1}), 153.5 (s, C^{A2}), 152.6 (s, C^{B2}), 150.3 (s, C^{A6}), 140.1 (s, C^{B4}), 139.7 (s, C^{A4}), 135.0 (s, C^{C3}), 134.0 (pseudo-t, $^2J_{CP}$ = 7.5 Hz, C^{D2}), 133.8 (pseudo-t, $^2J_{CP}$ = 7.2 Hz, C$^{D2'}$), 133.2 (s, C^{D4}), 132.1 (pseudo-t, $^1J_{CP}$ = 15.7 Hz, C^{D1}+C$^{D1'}$), 131.0 (s, C$^{D4'}$), 131.0 (s, C^{C5}), 129.7 (pseudo-t, $^3J_{CP}$ = 4.7 Hz, C^{D3}+C$^{D3'}$), 127.1 (s, C^{B5}), 126.6 (s, C^{A5}), 126.1 (pseudo-t, $^3J_{CP}$ = 2.3 Hz, C^{C4}), 125.1 (pseudo-t, $^1J_{CP}$ = 14.3 Hz, C^{C2}), 123.6 (s, C^{A3}), 121.2 (pseudo-t, $^3J_{CP}$ = 2.3 Hz, C^{C6}), 120.9 (s, C^{B3}), 26.4 (s, C$^{B6\text{-}Me}$).
^{19}F{^{1}H} NMR (202 MHz, 298 K, acetone-d$_6$) δ/ppm –72.7 (d, $^1J_{FP}$ = 707 Hz).
^{31}P{^{1}H} NMR (202 MHz, 298 K, acetone-d$_6$) δ/ppm –12.9 (broad, FWHM ≈ 320 Hz, P^{POP}), –144.2 (hept., $^1J_{PF}$ = 707 Hz, P^{PF6}).
ESI(+)-MS (DCM/MeOH, m/z): 600.88 [M–PF$_6$–(bpy)]$^+$ (calc. 601.09), 770.88 [M–PF$_6$]$^+$ (base peak, calc. 717.17).
ESI(–)-MS (DCM/MeOH, m/z): 144.96 [PF$_6$]$^–$ (calc. 144.96).
Found: C 61.53, H 4.24, N 3.33; $C_{47}H_{38}CuF_6N_2OP_3$ requires C 61.54, H 4.18, N 3.05.

[Cu(POP)(Me$_2$bpy)][PF$_6$]

The reagents were POP (136 mg, 0.252 mmol), Me$_2$bpy (46.2 mg, 0.251 mmol) and [Cu(MeCN)$_4$][PF$_6$] (93.2 mg, 0.250 mmol). [Cu(POP)(Me$_2$bpy)][PF$_6$] was isolated as a yellow solid (191 mg, 0.205 mmol, 82%).
^{1}H NMR (500 MHz, acetone-d$_6$) δ/ppm 8.23 (d, $^2J_{HH}$ = 8.0, 2H, H^{B3}), 8.02 (m, 2H, H^{B4}), 7.45 (m, 2H, H^{C5}), 7.44 (m, 2H, H^{B5}), 7.35 (m, 4H, H^{D4}), 7.30 (m, 2H, H^{C4}), 7.29 (m, 2H, H^{C3}), 7.21 (m, 8H, H^{D3}), 7.11 (m, 8H, H^{D2}), 7.06 (m, 2H, H^{C6}), 2.32 (s, H$^{B6\text{-}Me}$).
^{13}C{^{1}H} NMR (126 MHz, acetone-d$_6$) δ/ppm 159.6 (s, C^{B6}), 159.1 (pseudo-t, $^2J_{CP}$ = 6.0 Hz, C^{C1}), 153.3 (s, C^{B2}), 139.9 (s, C^{B4}), 134.5 (s, C^{C3}), 133.9 (pseudo-t, $^2J_{CP}$ = 7.8 Hz, C^{D2}), 133.3 (s, C^{C5}), 132.8 (pseudo-t, $^1J_{CP}$ = 16.2 Hz, C^{C2}), 130.8 (s, C^{D4}), 129.6 (pseudo-t, $^3J_{CP}$ = 4.6 Hz, C^{D3}), 127.1 (s, C^{B5}), 126.2 (pseudo-t, $^1J_{CP}$ = 13.9 Hz, C^{D1}), 126.2 (pseudo-t, $^3J_{CP}$ = 2.2 Hz, C^{C4}), 121.1 (pseudo-t, $^3J_{CP}$ = 2.0 Hz, C^{C6}), 120.9 (s, C^{B3}), 26.9 (s, C$^{B6\text{-}Me}$).
^{19}F{^{1}H} NMR (202 MHz, 298 K, acetone-d$_6$) δ/ppm –72.7 (d, $^1J_{FP}$ = 707 Hz).
^{31}P{^{1}H} NMR (202 MHz, 298 K, acetone-d$_6$) δ/ppm –13.6 (broad, FWHM ≈ 300 Hz, P^{POP}), –144.2 (hept., $^1J_{PF}$ = 707 Hz, P^{PF6}).
ESI(+)-MS (DCM/MeOH, m/z): 600.88 [M–PF$_6$–(Me$_2$bpy)]$^+$ (calc. 601.09), 784.88 [M–PF$_6$]$^+$ (base peak, calc. 785.19).
ESI(–)-MS (DCM/MeOH, m/z): 144.96 [PF$_6$]$^–$ (calc. 144.96).
Found: C 62.03, H 4.51, N 2.94; $C_{48}H_{40}CuF_6N_2OP_3$ requires C 61.90, H 4.33, N 3.01.

[Cu(xantphos)(Mebpy)][PF$_6$]

The reagents were xantphos (175 mg, 0.302 mmol), Mebpy (50.1 mg, 0.294 mmol) and [Cu(MeCN)$_4$][PF$_6$] (111 mg, 0.298 mmol). [Cu(xantphos)(Mebpy)][PF$_6$] was isolated as a yellow solid (264 mg, 0.276 mmol, 94%).
^{1}H NMR (500 MHz, acetone-d$_6$) δ/ppm 8.54 (m, 1H, H^{A3}), 8.54 (m, 1H, H^{A6}), 8.45 (d, $^2J_{HH}$ = 8.0 Hz, 1H, H^{B3}), 8.15 (m, 1H, H^{B4}), 8.10 (m, 1H, H^{A4}), 7.85 (dd, $^2J_{HH}$ = 7.8 Hz, $^3J_{HH}$ = 1.4 Hz, 2H, H^{C5}), 7.52 (d, $^2J_{HH}$ = 7.4 Hz, 1H, H^{A5}), 7.49 (m, 1H, H^{B5}), 7.39 (m, 2H, H^{D4}), 7.34 (m, 2H, H$^{D4'}$), 7.30 (d, $^2J_{HH}$ = 7.7 Hz, 2H, H^{C4}), 7.26 (m, 4H, H$^{D3'}$), 7.19 (m, 4H, H^{D3}), 7.17 (m, 4H, $^{D2'}$), 6.92 (m, 4H, H^{D2}), 6.66 (m, 2H, H^{C3}), 2.03 (m, 3H, H$^{B6\text{-}Me}$), 1.89 (m, 3H, H$^{xantphos\text{-}Me'}$), 1.69 (m, 3H, H$^{xantphos\text{-}Me}$).
^{13}C{^{1}H} NMR (126 MHz, acetone-d$_6$) δ/ppm 159.4 (s, C^{B6}), 155.8 (pseudo-t, $^2J_{CP}$ = 6.1 Hz, C^{C1}), 153.3 (s, C^{A2}), 152.4 (s, C^{B2}), 149.9 (s, C^{A6}), 140.3 (s, C^{B4}), 139.9 (s, C^{A4}), 135.0 (s, C^{C6}), 133.9 (pseudo-t, $^2J_{CP}$ = 8.0 Hz, C^{D2}), 133.5 (pseudo-t, $^2J_{CP}$ = 7.8 Hz, C$^{D2'}$), 132.6 (pseudo-t, $^1J_{CP}$ = 16.5 Hz, C$^{D1'}$), 132.5 (pseudo-t, $^1J_{CP}$ = 17.6 Hz, C^{D1}), 131.5 (s, C^{C3}), 131.1 (s, C$^{D4'}$), 131.0 (s, C^{D4}), 129.9 (pseudo-t, $^3J_{CP}$ = 4.8 Hz, C$^{D3'}$), 129.7 (pseudo-t, $^3J_{CP}$ = 4.7 Hz, C^{D3}), 128.5 (s, C^{C5}), 127.1 (s, C^{A5}), 127.0 (s, C^{B5}), 126.3 (pseudo-t, $^3J_{CP}$ = 2.6 Hz, C^{C4}), 123.8 (s, C^{A3}), 121.5 (pseudo-t, $^1J_{CP}$ = 13.5 Hz, C^{C2}), 121.1 (s, C^{B3}), 36.9 (s, C$^{xantphos\text{-}bridge}$), 30.3 (s, C$^{xantphos\text{-}Me}$), 26.4 (s, C$^{xantphos\text{-}Me'}$), 26.3 (s, C$^{B6\text{-}Me}$).
^{19}F{^{1}H} NMR (202 MHz, 298 K, acetone-d$_6$) δ/ppm –72.6 (d, $^1J_{FP}$ = 707 Hz).
^{31}P{^{1}H} NMR (202 MHz, 298 K, acetone-d$_6$) δ/ppm –12.8 (broad, FWHM ≈ 280 Hz, P^{xantphos}), –144.2 (hept., $^1J_{PF}$ = 707 Hz, P^{PF6}).
ESI(+)-MS (DCM/MeOH, m/z): 641.04 [M–PF$_6$–(Mebpy)]$^+$ (calc. 641.12), 811.14 [M–PF$_6$]$^+$ (base peak, calc. 811.21).
ESI(–)-MS (DCM/MeOH, m/z): 144.94 [PF$_6$]$^–$ (calc. 144.96).
Found: C 62.68, H 4.87, N 3.00; $C_{50}H_{42}CuF_6N_2OP_3$ requires C 62.73, H 4.42, N 2.93.

This journal is © The Royal Society of Chemistry 20xx

Please do not adjust margins

Please do not adjust margins

[Cu(xantphos)(Me$_2$bpy)][PF$_6$]

The reagents were xantphos (145 mg, 0.250 mmol), Me$_2$bpy (0.45.8 mg, 0.248 mmol) and [Cu(MeCN)$_4$][PF$_6$] (94.3 mg, 0.253 mmol). [Cu(xantphos)(Me$_2$bpy)][PF$_6$] was isolated as a yellow solid (214 mg, 0.221 mmol, 89%).

^{1}H NMR (500 MHz, acetone-d$_6$) δ/ppm 8.19 (d, $^2J_{HH}$ = 8.0 Hz, 2H, H^{B3}), 7.98 (m, 2H, H^{B4}), 7.83 (dd, $^2J_{HH}$ = 7.9, $^3J_{HH}$ = 1.4 Hz, 2H, H^{C5}), 7.42 (m, 4H, H^{D4}), 7.39 (m, 2H, H^{B5}), 7.31 (m, 2H, H^{C4}), 7.23 (m, 8H, H^{D3}), 7.17 (m, 8H, H^{D2}), 6.95 (m, 2H, H^{C3}), 2.14 (s, 6H, $H^{6\text{-}Me}$), 1.76 (s, 6H, $H^{xantphos\text{-}Me}$).

^{13}C{^{1}H} NMR (126 MHz, acetone-d$_6$) δ/ppm 159.2 (s, C^{B6}), 155.9 (pseudo-t, $^2J_{CP}$ = 6.5 Hz, C^{C1}), 153.1 (s, C^{B2}), 139.9 (s, C^{B4}), 134.9 (s, C^{C6}), 134.0 (pseudo-t, $^2J_{CP}$ = 7.6 Hz, C^{D2}), 132.5 (pseudo-t, $^1J_{CP}$ = 16.0 Hz, C^{D1}), 131.1 (s, C^{C3}), 131.0 (s, C^{D4}), 129.7 (pseudo-t, $^3J_{CP}$ = 4.5 Hz, C^{D3}), 128.6 (s, C^{C5}), 126.7 (s, C^{B5}), 126.3 (pseudo-t, $^3J_{CP}$ = 2.3 Hz, C^{C4}), 122.7 (pseudo-t, $^1J_{CP}$ = 12.2 Hz, C^{C2}), 121.0 (s, C^{B3}), 36.9 (s, $C^{xantphos\text{-}bridge}$), 28.6 (s, $C^{B6\text{-}Me}$), 27.0 (s, $C^{xantphos\text{-}Me}$).

^{19}F{^{1}H} NMR (202 MHz, 298 K, acetone-d$_6$) δ/ppm –72.6 (d, $^1J_{FP}$ = 707 Hz).

^{31}P{^{1}H} NMR (202 MHz, 298 K, acetone-d$_6$) δ/ppm –13.7 (broad, FWHM ≈ 285 Hz, $P^{xantphos}$), –144.2 (hept., $^1J_{PF}$ = 707 Hz, P^{PF6}).

ESI(+)-MS (DCM/MeOH, m/z): 641.05 [M–PF$_6$–(Me$_2$bpy)]$^+$ (calc. 641.12), 825.14 [M–PF$_6$]$^+$ (base peak, calc. 825.22).

ESI(–)-MS (DCM/MeOH, m/z): 144.94 [PF$_6$]$^-$ (calc. 144.96).

Found: C 62.96, H 4.87, N 3.11; $C_{51}H_{44}CuF_6N_2OP_3$ requires C 63.06, H 4.57, N 2.88.

[Cu(POP)(Mebpy)][BF$_4$]

The reagents were POP (136 mg, 0.252 mmol), Mebpy (42.6 mg, 0.250 mmol) and [Cu(MeCN)$_4$][BF$_4$] (78.7 mg, 0.250 mmol). [Cu(POP)(Mebpy)][BF$_4$] was isolated as a yellow solid (182 mg, 0.213 mmol, 85%).

^{1}H NMR (500 MHz, acetone-d$_6$) δ/ppm 8.68 (d, $^2J_{HH}$ = 5.1 Hz, 1H, H^{A6}), 8.50 (d, $^2J_{HH}$ = 8.2 Hz, 1H, H^{A3}), 8.42 (d, $^2J_{HH}$ = 7.9 Hz, 1H, H^{B3}), 8.10 (m, 1H, H^{B4}), 8.08 (m, 1H, H^{A4}), 7.47 (d, $^2J_{HH}$ = 7.7 Hz, 1H, H^{B5}), 7.43 (m, 2H, H^{D4}), 7.41 (m, 1H, H^{A5}), 7.41 (m, 2H, H^{C5}), 7.38 (m, 2H, $H^{D4'}$), 7.27 (m, 4H, H^{D3}), 7.27 (m, 4H, $H^{D3'}$), 7.19 (m, 2H, H^{C6}), 7.15 (m, 2H, H^{C4}), 7.14 (m, 4H, H^{D2}), 7.10 (m, 4H, $H^{D2'}$), 6.90 (m, 2H, H^{C3}), 2.35 (s, 3H, $H^{B6\text{-}Me}$).

^{13}C{^{1}H} NMR (126 MHz, acetone-d$_6$) δ/ppm 159.9 (s, C^{B6}), 158.9 (pseudo-t, $^2J_{CP}$ = 6.0 Hz, C^{C1}), 153.4 (s, C^{A2}), 152.6 (s, C^{B2}), 150.3 (s, C^{A6}), 140.1 (s, C^{B4}), 139.7 (s, C^{A4}), 135.0 (s, C^{C3}), 134.1 (pseudo-t, $^2J_{CP}$ = 7.5 Hz, C^{D2}), 133.8 (pseudo-t, $^2J_{CP}$ = 7.2 Hz, $C^{D2'}$), 133.2 (s, C^{D4}), 132.1 (pseudo-t, $^1J_{CP}$ = 15.7 Hz, C^{D1}+$C^{D1'}$), 131.0 (s, $C^{D4'}$), 131.0 (s, C^{C5}), 129.7 (pseudo-t, $^3J_{CP}$ = 4.7 Hz, C^{D3}+$C^{D3'}$), 127.1 (s, C^{B5}), 126.6 (s, C^{A5}), 126.1 (pseudo-t,$^3J_{CP}$ = 2.3 Hz, C^{C4}), 125.1 (pseudo-t, $^1J_{CP}$ = 14.3 Hz, C^{C2}), 123.6 (s, C^{A3}), 121.2 (pseudo-t, $^3J_{CP}$ = 2.3 Hz, C^{C6}), 120.9 (s, C^{B3}), 26.4 (s, $C^{B6\text{-}Me}$).

^{11}B{^{1}H} NMR (160 MHz, 298 K, acetone-d$_6$) δ/ppm –0.9 (s).

^{19}F{^{1}H} NMR (202 MHz, 298 K, acetone-d$_6$) δ/ppm –151.8 (s).

^{31}P{^{1}H} NMR (202 MHz, 298 K, acetone-d$_6$) δ/ppm –13.0 (broad, FWHM ≈ 310 Hz).

ESI(+)-MS (DCM/MeOH, m/z): 600.88 [M–BF$_4$–(Mebpy)]$^+$ (calc. 601.09), 770.88 [M–BF$_4$]$^+$ (base peak, calc. 771.17).

ESI(–)-MS (DCM/MeOH, m/z): 86.96 [BF$_4$]$^-$ (calc. 87.00).

Found: C 65.50, H 4.37, N 3.28; $C_{47}H_{38}BCuF_4N_2OP_2$ requires C 65.71, H 4.46, N 3.26.

[Cu(POP)(Me$_2$bpy)][BF$_4$]

The reagents were POP (136 mg, 0.252 mmol), Me$_2$bpy (46.1 mg, 0.250 mmol) and [Cu(MeCN)$_4$][BF$_4$] (78.7 mg, 0.250 mmol). [Cu(POP)(Me$_2$bpy)][BF$_4$] was isolated as a yellow solid (192 mg, 0.220 mmol, 88%).

^{1}H NMR (500 MHz, acetone-d$_6$) δ/ppm 8.24 (d, $^2J_{HH}$ = 8.0, 2H, H^{B3}), 8.02 (m, 2H, H^{B4}), 7.45 (m, 2H, H^{C5}), 7.44 (m, 2H, H^{B5}), 7.35 (m, 4H, H^{D4}), 7.30 (m, 2H, H^{C4}), 7.28 (m, 2H, H^{C3}), 7.21 (m, 8H, H^{D3}), 7.11 (m, 8H, H^{D2}), 7.06 (m, 2H, H^{C6}), 2.32 (s, $H^{B6\text{-}Me}$).

^{13}C{^{1}H} NMR (126 MHz, acetone-d$_6$) δ/ppm 159.6 (s, C^{B6}), 159.1 (pseudo-t, $^2J_{CP}$ = 6.0 Hz, C^{C1}), 153.3 (s, C^{B2}), 139.9 (s, C^{B4}), 134.5 (s, C^{C3}), 133.9 (pseudo-t, $^2J_{CP}$ = 7.8 Hz, C^{D2}), 133.3 (s, C^{C5}), 132.8 (pseudo-t, $^1J_{CP}$ = 16.3 Hz, C^{C2}), 130.8 (s, C^{D4}), 129.6 (pseudo-t, $^3J_{CP}$ = 4.6 Hz, C^{D3}), 127.1 (s, C^{B5}), 126.2 (pseudo-t, $^1J_{CP}$ = 14.0 Hz, C^{D1}), 126.2 (pseudo-t, $^3J_{CP}$ = 2.2 Hz, C^{C4}), 121.0 (pseudo-t, $^3J_{CP}$ = 2.0 Hz, C^{C6}), 120.9 (s, C^{B3}), 26.9 (s, $C^{B6\text{-}Me}$).

^{11}B{^{1}H} NMR (160 MHz, 298 K, acetone-d$_6$) δ/ppm –0.9 (s).

^{19}F{^{1}H} NMR (202 MHz, 298 K, acetone-d$_6$) δ/ppm –151.8 (s).

^{31}P{^{1}H} NMR (202 MHz, 298 K, acetone-d$_6$) δ/ppm –13.7 (broad, FWHM ≈ 310 Hz).

ESI(+)-MS (DCM/MeOH, m/z): 600.88 [M–BF$_4$–(Me$_2$bpy)]$^+$ (calc. 601.09), 784.88 [M–BF$_4$]$^+$ (base peak, calc. 785.19).

ESI(–)-MS (DCM/MeOH, m/z): 86.96 [BF$_4$]$^-$ (calc. 87.00).

Found: C 65.70, H 4.51, N 3.26; $C_{48}H_{40}BCuF_4N_2OP_2$ requires C 66.03, H 4.62, N 3.21.

[Cu(xantphos)(Mebpy)][BF$_4$]

The reagents were xantphos (147 mg, 0.253 mmol), Mebpy (42.5 mg, 0.254 mmol) and [Cu(MeCN)$_4$][BF$_4$] (78.6 mg, 0.250 mmol). [Cu(xantphos)(Mebpy)][BF$_4$] was isolated as a yellow solid (205 mg, 0.228 mmol, 91%).

This journal is © The Royal Society of Chemistry 20xx

Please do not adjust margins

Please do not adjust margins

^{1}H NMR (500 MHz, acetone-d$_6$) δ/ppm 8.54 (m, 1H, H^{A3}), 8.54 (m, 1H, H^{A6}), 8.46 (d, $^2J_{HH}$ = 8.0 Hz, 1H, H^{B3}), 8.15 (m, 1H, H^{B4}), 8.10 (m, 1H, H^{A4}), 7.85 (dd, $^2J_{HH}$ = 7.8 Hz, $^3J_{HH}$ = 1.4 Hz, 2H, H^{C5}), 7.52 (d, $^2J_{HH}$ = 7.6 Hz, 1H, H^{A5}), 7.49 (m, 1H, H^{B5}), 7.39 (m, 2H, H^{D4}), 7.34 (m, 2H, H$^{D4'}$), 7.30 (d, $^2J_{HH}$ = 7.7 Hz, 2H, H^{C4}), 7.26 (m, 4H, H$^{D3'}$), 7.19 (m, 4H, H^{D3}), 7.17 (m, 4H, $^{D2'}$), 6.92 (m, 4H, H^{D2}), 6.66 (m, 2H, H^{C3}), 2.03 (m, 3H, H$^{B6\text{-}Me}$), 1.89 (m, 3H, H$^{xantphos\text{-}Me'}$), 1.69 (m, 3H, H$^{xantphos\text{-}Me}$).
^{13}C{^{1}H} NMR (126 MHz, acetone-d$_6$) δ/ppm 159.3 (s, C^{B6}), 155.9 (pseudo-t, $^2J_{CP}$ = 6.3 Hz, C^{C1}), 153.3 (s, C^{A2}), 152.4 (s, C^{B2}), 149.9 (s, C^{A6}), 140.3 (s, C^{B4}), 139.9 (s, C^{A4}), 135.0 (s, C^{C6}), 133.9 (pseudo-t, $^2J_{CP}$ = 8.0 Hz, C^{D2}), 133.5 (pseudo-t, $^2J_{CP}$ = 7.8 Hz, C$^{D2'}$), 132.6 (pseudo-t, $^1J_{CP}$ = 16.6 Hz, C$^{D1'}$), 132.5 (pseudo-t, $^1J_{CP}$ = 17.6 Hz, C^{D1}), 131.5 (s, C^{C3}), 131.1 (s, C$^{D4'}$), 130.9 (s, C^{D4}), 129.9 (pseudo-t, $^3J_{CP}$ = 4.7 Hz, C$^{D3'}$), 129.7 (pseudo-t, $^3J_{CP}$ = 4.7 Hz, C^{D3}), 128.5 (s, C^{C5}), 127.1 (s, C^{A5}), 127.0 (s, C^{B5}), 126.3 (pseudo-t, $^3J_{CP}$ = 2.5 Hz, C^{C4}), 123.8 (s, C^{A3}), 121.5 (pseudo-t, $^1J_{CP}$ = 13.7 Hz, C^{C2}), 121.1 (s, C^{B3}), 36.9 (s, C$^{xantphos\text{-}bridge}$), 30.3 (s, C$^{xantphos\text{-}Me}$), 26.4 (s, C$^{xantphos\text{-}Me'}$), 26.3 (s, C$^{B6\text{-}Me}$).
^{11}B{^{1}H} NMR (160 MHz, 298 K, acetone-d$_6$) δ/ppm –0.9 (s).
^{19}F{^{1}H} NMR (202 MHz, 298 K, acetone-d$_6$) δ/ppm –151.9 (s).
^{31}P{^{1}H} NMR (202 MHz, 298 K, acetone-d$_6$) δ/ppm –12.9 (broad, FWHM ≈ 320 Hz).
ESI(+)-MS (DCM/MeOH, m/z): 640.88 [M–BF$_4$–(Mebpy)]$^+$ (calc. 641.12), 810.88 [M–BF$_4$]$^+$ (base peak, calc. 811.21).
ESI(–)-MS (DCM/MeOH, m/z): 86.96 [BF$_4$]$^–$ (calc. 87.00).
Found: C 66.48, H 5.06, N 3.31; $C_{50}H_{42}BCuF_4N_2OP_2$ requires C 66.79, H 4.71, N 3.12.

[Cu(xantphos)(Me$_2$bpy)][BF$_4$]

The reagents were xantphos (145 mg, 0.250 mmol), Me$_2$bpy (46.1 mg, 0.250 mmol) and [Cu(MeCN)$_4$][BF$_4$] (78.7 mg, 0.250 mmol). [Cu(xantphos)(Me$_2$bpy)][BF$_4$] was isolated as a yellow solid (189 mg, 0.208 mmol, 83%).
^{1}H NMR (500 MHz, acetone-d$_6$) δ/ppm
8.20 (d, $^2J_{HH}$ = 8.0 Hz, 2H, H^{B3}), 7.98 (m, 2H, H^{B4}), 7.83 (dd, $^2J_{HH}$ = 7.9, $^3J_{HH}$ = 1.4 Hz, 2H, H^{C5}), 7.41 (m, 4H, H^{D4}), 7.39 (m, 2H, H^{B5}), 7.31 (m, 2H, H^{C4}), 7.23 (m, 8H, H^{D3}), 7.17 (m, 8H, H^{D2}), 6.94 (m, 2H, H^{C3}), 2.14 (s, 6H, H$^{6\text{-}Me}$), 1.76 (s, 6H, H$^{xantphos\text{-}Me}$).
^{13}C{^{1}H} NMR (126 MHz, acetone-d$_6$) δ/ppm 159.2 (s, C^{B6}), 155.9 (pseudo-t, $^2J_{CP}$ = 6.5 Hz, C^{C1}), 153.1 (s, C^{B2}), 139.9 (s, C^{B4}), 134.9 (s, C^{C6}), 134.0 (pseudo-t, $^2J_{CP}$ = 7.7 Hz, C^{D2}), 132.5 (pseudo-t, $^1J_{CP}$ = 16.0 Hz, C^{D1}), 131.1 (s, C^{C3}), 131.0 (s, C^{D4}), 129.7 (pseudo-t, $^3J_{CP}$ = 4.6 Hz, C^{D3}), 128.6 (s, C^{C5}), 126.7 (s, C^{B5}), 126.3 (pseudo-t, $^3J_{CP}$ = 2.3 Hz, C^{C4}), 122.7 (pseudo-t, $^1J_{CP}$ = 12.2 Hz, C^{C2}), 121.1 (s, C^{B3}), 36.9 (s, C$^{xantphos\text{-}bridge}$), 28.6 (s, C$^{B6\text{-}Me}$), 27.0 (s, C$^{xantphos\text{-}Me}$).
^{11}B{^{1}H} NMR (160 MHz, 298 K, acetone-d$_6$) δ/ppm –0.9 (s).
^{19}F{^{1}H} NMR (202 MHz, 298 K, acetone-d$_6$) δ/ppm –151.9 (s).
^{31}P{^{1}H} NMR (202 MHz, 298 K, acetone-d$_6$) δ/ppm –12.9 (broad, FWHM ≈ 270 Hz, P^{xantphos}).
ESI(+)-MS (DCM/MeOH, m/z): 640.88 [M–BF$_4$–(Me$_2$bpy)]$^+$ (calc. 641.12), 824.88 [M–BF$_4$]$^+$ (base peak, calc. 825.22).
ESI(–)-MS (DCM/MeOH, m/z): 86.96 [BF$_4$]$^–$ (calc. 87.00).
Found: C 50.79, H 3.40, N 2.56; $C_{48}H_{34}Br_2CuF_6N_2OP_3\cdot CH_2Cl_2$ requires C 50.26, H 3.18, N 2.39.

[Cu(POP)(Mebpy)][BPh$_4$]

The reagents were [Cu(POP)(Mebpy)][PF$_6$] (153 mg, 0.167 mmol) and NaBPh$_4$ (149.4 mg, 0.436 mmol); two ion exchange steps executed, half amount of NaBPh$_4$ added per ion exchange step. [Cu(POP)(Mebpy)][BPh$_4$] was isolated as a yellow solid (131 mg, 0.120 mmol, 72%).
^{1}H NMR (500 MHz, acetone-d$_6$) δ/ppm 8.65 (d, $^2J_{HH}$ = 5.1 Hz, 1H, H^{A6}), 8.42 (d, $^2J_{HH}$ = 8.2 Hz, 1H, H^{A3}), 8.34 (d, $^2J_{HH}$ = 7.9 Hz, 1H, H^{B3}), 8.05 (m, 1H, H^{B4}), 8.02 (m, 1H, H^{A4}), 7.42 (d, $^2J_{HH}$ = 7.7 Hz, 1H, H^{B5}), 7.41 (m, 2H, H^{D4}), 7.37 (m, 1H, H^{A5}), 7.36 (m, 2H, H^{C5}), 7.35 (m, 2H, H$^{D4'}$), 7.34 (m, 8H, H^{E3}), 7.26 (m, 4H, H^{D3}), 7.26 (m, 4H, H$^{D3'}$), 7.16 (m, 2H, H^{C6}), 7.13 (m, 2H, H^{C4}), 7.12 (m, 4H, H^{D2}), 7.10 (m, 4H, H$^{D2'}$), 6.91 (m, 2H, H^{C3}), 6.91 (m, 8H, H^{E2}), 6.76 (m, 4H, H^{E4}), 2.32 (s, 3H, H$^{B6\text{-}Me}$).
^{13}C{^{1}H} NMR (126 MHz, acetone-d$_6$) δ/ppm 164.7 (q, $^1J_{CB}$ = 49.1 Hz, C^{E1}), 159.8 (s, C^{B6}), 158.6 (pseudo-t, $^2J_{CP}$ = 6.0 Hz, C^{C1}), 153.2 (s, C^{A2}), 152.3 (s, C^{B2}), 150.0 (s, C^{A6}), 139.7 (s, C^{B4}), 139.4 (s, C^{A4}), 136.8 (pseudo-q, $^2J_{CP}$ = 1.4 Hz, C^{E3}), 134.7 (s, C^{C3}), 133.7 (pseudo-t, $^2J_{CP}$ = 7.5 Hz, C^{D2}), 133.5 (pseudo-t, $^2J_{CP}$ = 7.2 Hz, C$^{D2'}$), 132.9 (s, C^{D4}), 131.9 (pseudo-t, $^1J_{CP}$ = 15.7 Hz, C^{D1}+C$^{D1'}$), 130.7 (s, C$^{D4'}$), 130.7 (s, C^{C5}), 129.5 (pseudo-t, $^3J_{CP}$ = 4.7 Hz, C^{D3}+C$^{D3'}$), 126.8 (s, C^{B5}), 126.4 (s, C^{A5}), 125.8 (pseudo-t, $^3J_{CP}$ = 2.3 Hz, C^{C4}), 125.8 (pseudo-q, $^2J_{CP}$ = 3.6 Hz, C^{E2}), 124.8 (pseudo-t, $^1J_{CP}$ = 14.3 Hz, C^{C2}), 123.3 (s, C^{A3}), 122.0 (m, C^{E4}), 121.0 (pseudo-t, $^3J_{CP}$ = 2.3 Hz, C^{C6}), 120.6 (s, C^{B3}), 26.2 (s, C$^{B6\text{-}Me}$).
^{11}B{^{1}H} NMR (160 MHz, 298 K, acetone-d$_6$) δ/ppm –6.9 (s).
^{31}P{^{1}H} NMR (202 MHz, 298 K, acetone-d$_6$) δ/ppm –12.9 (broad, FWHM ≈ 310 Hz).
ESI(+)-MS (DCM/MeOH, m/z): 601.66 [M–BPh$_4$–(Mebpy)]$^+$ (calc. 601.09), 771.66 [M–BPh$_4$]$^+$ (base peak, calc. 771.17).
ESI(–)-MS (DCM/MeOH, m/z): 318.88 [BPh$_4$]$^–$ (calc. 319.23).
Found: C 77.82, H 5.80, N 2.52; $C_{71}H_{58}BCuN_2OP_2$ requires C 78.12, H 5.36, N 2.57.

[Cu(POP)(Me$_2$bpy)][BPh$_4$]

This journal is © The Royal Society of Chemistry 20xx

Please do not adjust margins

Please do not adjust margins

The reagents were [Cu(POP)(Me_2bpy)][PF_6] (238 mg, 0.255 mmol) and $NaBPh_4$ (237 mg, 0.687 mmol); two ion exchange steps executed, half amount of $NaBPh_4$ added per ion exchange step. [Cu(POP)(Me_2bpy)][BPh_4] was isolated as a yellow solid (218 mg, 0.197 mmol, 77%).

^{1}H NMR (500 MHz, acetone-d$_6$) δ/ppm 8.15 (d, $^2J_{HH}$ = 8.0, 2H, H^{B3}), 7.96 (m, 2H, H^{B4}), 7.43 (m, 2H, H^{C5}), 7.39 (m, 2H, H^{B5}), 7.34 (m, 4H, H^{D4}), 7.34 (m, 8H, H^{E3}), 7.29 (m, 2H, H^{C4}), 7.28 (m, 2H, H^{C3}), 7.19 (m, 8H, H^{D3}), 7.10 (m, 8H, H^{D2}), 7.05 (m, 2H, H^{C6}), 6.91 (m, 8H, H^{E2}), 6.76 (m, 4H, H^{E4}), 2.31 (s, $H^{B6\text{-}Me}$).

^{13}C{^{1}H} NMR (126 MHz, acetone-d$_6$) δ/ppm 164.7 (q, $^1J_{CB}$ = 49.1 Hz, C^{E1}), 159.4 (s, C^{B6}), 158.9 (pseudo-t, $^2J_{CP}$ = 6.0 Hz, C^{C1}), 153.1 (s, C^{B2}), 139.6 (s, C^{B4}), 136.8 (pseudo-q, $^2J_{CP}$ = 1.4 Hz, C^{E3}), 134.2 (s, C^{C3}), 133.6 (pseudo-t, $^2J_{CP}$ = 7.8 Hz, C^{D2}), 133.0 (s, C^{C5}), 132.5 (pseudo-t, $^1J_{CP}$ = 16.3 Hz, C^{C2}), 130.5 (s, C^{D4}), 129.3 (pseudo-t, $^3J_{CP}$ = 4.6 Hz, C^{D3}), 126.8 (s, C^{B5}), 125.9 (pseudo-t, $^1J_{CP}$ = 14.0 Hz, C^{D1}), 125.9 (pseudo-t, $^3J_{CP}$ = 2.2 Hz, C^{C4}), 125.8 (pseudo-q, $^2J_{CP}$ = 3.6 Hz, C^{E2}), 122.0 (m, C^{E4}), 120.8 (pseudo-t, $^3J_{CP}$ = 2.0 Hz, C^{C6}), 120.7 (s, C^{B3}), 26.7 (s, $C^{B6\text{-}Me}$).

^{11}B{^{1}H} NMR (160 MHz, 298 K, acetone-d$_6$) δ/ppm –6.5 (s).

^{31}P{^{1}H} NMR (202 MHz, 298 K, acetone-d$_6$) δ/ppm –13.6 (broad, FWHM ≈ 250 Hz).

ESI(+)-MS (DCM/MeOH, m/z): 601.66 [M–BPh_4–(Me_2bpy)]$^+$ (calc. 601.09), 785.66 [M–BPh_4]$^+$ (base peak, calc. 785.19).

ESI(–)-MS (DCM/MeOH, m/z): 318.88 [BPh_4]$^-$ (calc. 319.23).

Found: C 78.17, H 6.23, N 2.34; $C_{72}H_{60}BCuN_2OP_2$ requires C 78.22, H 5.47, N 2.53.

[Cu(xantphos)(Mebpy)][BPh_4]

The reagents were [Cu(xantphos)(Mebpy)][PF_6] (149 mg, 0.156 mmol) and $NaBPh_4$ (143 mg, 0.418 mmol); two ion exchange steps executed, half amount of $NaBPh_4$ added per ion exchange step. [Cu(xantphos)(Mebpy)][BPh_4] was isolated as a yellow solid (141 mg, 0.125 mmol, 80%).

^{1}H NMR (500 MHz, acetone-d$_6$) δ/ppm 8.53 (m, 1H, H^{A3}), 8.52 (m, 1H, H^{A6}), 8.43 (d, $^2J_{HH}$ = 8.0 Hz, 1H, H^{B3}), 8.12 (m, 1H, H^{B4}), 8.08 (m, 1H, H^{A4}), 7.85 (dd, $^2J_{HH}$ = 7.8 Hz, $^3J_{HH}$ = 1.4 Hz, 2H, H^{C5}), 7.50 (d, $^2J_{HH}$ = 7.6 Hz, 1H, H^{A5}), 7.48 (m, 1H, H^{B5}), 7.38 (m, 2H, H^{D4}), 7.35 (m, 2H, $H^{D4'}$), 7.34 (m, 8H, H^{E3}), 7.29 (d, $^2J_{HH}$ = 7.7 Hz, 2H, H^{C4}), 7.26 (m, 4H, $H^{D3'}$), 7.19 (m, 4H, H^{D3}), 7.17 (m, 4H, $H^{D2'}$), 6.92 (m, 4H, H^{D2}), 6.91 (m, 8H, H^{E2}), 6.76 (m, 4H, H^{E4}), 6.66 (m, 2H, H^{C3}), 2.03 (m, 3H, $H^{B6\text{-}Me}$), 1.89 (m, 3H, $H^{xantphos\text{-}Me'}$), 1.68 (m, 3H, $H^{xantphos\text{-}Me}$).

^{13}C{^{1}H} NMR (126 MHz, acetone-d$_6$) δ/ppm 164.7 (q, $^1J_{CB}$ = 49.1 Hz, C^{E1}), 158.5 (s, C^{B6}), 153.1 (m, C^{C1}), 152.1 (s, C^{A2}), 149.7 (s, C^{B2}), 149.1 (s, C^{A6}), 140.1 (s, C^{B4}), 139.6 (s, C^{A4}), 136.8 (pseudo-q, $^2J_{CP}$ = 1.4 Hz, C^{E3}), 134.8 (s, C^{C6}), 133.6 (pseudo-t, $^2J_{CP}$ = 8.0 Hz, C^{D2}), 133.3 (pseudo-t, $^2J_{CP}$ = 7.8 Hz, $C^{D2'}$), 132.3 (pseudo-t, $^1J_{CP}$ = 16.6 Hz, $C^{D1'}$), 132.2 (pseudo-t, $^1J_{CP}$ = 17.6 Hz, C^{D1}), 131.3 (s, C^{C3}), 131.2 (s, $C^{D4'}$), 130.8 (s, C^{D4}), 129.6 (pseudo-t, $^3J_{CP}$ = 4.7 Hz, $C^{D3'}$), 129.5 (pseudo-t, $^3J_{CP}$ = 4.7 Hz, C^{D3}), 128.3 (s, C^{C5}), 126.8 (s, C^{A5}), 126.8 (s, C^{B5}), 126.1 (pseudo-t, $^3J_{CP}$ = 2.5 Hz, C^{C4}), 125.8 (pseudo-q, $^2J_{CP}$ = 3.6 Hz, C^{E2}), 123.5 (s, C^{A3}), 122.0 (m, C^{E4}), 121.3 (pseudo-t, $^1J_{CP}$ = 13.7 Hz, C^{C2}), 120.8 (s, C^{B3}), 36.6 (s, $C^{xantphos\text{-}bridge}$), 30.1 (s, $C^{xantphos\text{-}Me}$), 26.2 (s, $C^{xantphos\text{-}Me'}$), 26.0 (s, $C^{B6\text{-}Me}$).

^{11}B{^{1}H} NMR (160 MHz, 298 K, acetone-d$_6$) δ/ppm –6.5 (s).

^{31}P{^{1}H} NMR (202 MHz, 298 K, acetone-d$_6$) δ/ppm –12.9 (broad, FWHM ≈ 270 Hz).

ESI(+)-MS (DCM/MeOH, m/z): 640.88 [M–BPh_4–(Mebpy)]$^+$ (calc. 641.12), 810.66 [M–BPh_4]$^+$ (base peak, calc. 811.21).

ESI(–)-MS (DCM/MeOH, m/z): 318.88 [BPh_4]$^-$ (calc. 319.23).

Found: C 50.79, H 3.40, N 2.56; $C_{48}H_{34}Br_2CuF_6N_2OP_3 \cdot CH_2Cl_2$ requires C 50.26, H 3.18, N 2.39.

[Cu(xantphos)(Me_2bpy)][BPh_4]

The reagents were [Cu(xantphos)(Mebpy)][PF_6] (195 mg, 0.201 mmol) and $NaBPh_4$ (184 mg, 0.535 mmol); two ion exchange steps executed, half amount of $NaBPh_4$ added per ion exchange step. [Cu(xantphos)(Me_2bpy)][BPh_4] was isolated as a yellow solid (153 mg, 0.176 mmol, 78%).

^{1}H NMR (500 MHz, acetone-d$_6$) δ/ppm 8.15 (d, $^2J_{HH}$ = 8.0 Hz, 2H, H^{B3}), 7.95 (m, 2H, H^{B4}), 7.83 (dd, $^2J_{HH}$ = 7.9, $^3J_{HH}$ = 1.4 Hz, 2H, H^{C5}), 7.40 (m, 4H, H^{D4}), 7.39 (m, 2H, H^{B5}), 7.34 (m, 8H, H^{E3}), 7.30 (m, 2H, H^{C4}), 7.22 (m, 8H, H^{D3}), 7.17 (m, 8H, H^{D2}), 6.95 (m, 2H, H^{C3}), 6.91 (m, 8H, H^{E2}), 6.76 (m, 4H, H^{E4}), 2.13 (s, 6H, $H^{6\text{-}Me}$), 1.75 (s, 6H, $H^{xantphos\text{-}Me}$).

^{13}C{^{1}H} NMR (126 MHz, acetone-d$_6$) δ/ppm 164.7 (q, $^1J_{CB}$ = 49.1 Hz, C^{E1}), 159.1 (s, C^{B6}), 155.8 (pseudo-t, $^2J_{CP}$ = 6.5 Hz, C^{C1}), 153.1 (s, C^{B2}), 139.7 (s, C^{B4}), 136.8 (pseudo-q, $^2J_{CP}$ = 1.4 Hz, C^{E3}), 134.9 (s, C^{C6}), 133.8 (pseudo-t, $^2J_{CP}$ = 7.7 Hz, C^{D2}), 132.4 (pseudo-t, $^2J_{CP}$ = 16.0 Hz, C^{D1}), 131.0 (s, C^{C3}), 130.9 (s, C^{D4}), 129.6 (pseudo-t, $^3J_{CP}$ = 4.6 Hz, C^{D3}), 128.5 (s, C^{C5}), 126.6 (s, C^{B5}), 126.2 (pseudo-t, $^3J_{CP}$ = 2.3 Hz, C^{C4}), 125.8 (pseudo-q, $^2J_{CP}$ = 3.6 Hz, C^{E2}), 122.2 (pseudo-t, $^1J_{CP}$ = 12.2 Hz, C^{C2}), 122.0 (m, C^{E4}), 120.8 (s, C^{B3}), 36.8 (s, $C^{xantphos\text{-}bridge}$), 28.4 (s, $C^{B6\text{-}Me}$), 26.8 (s, $C^{xantphos\text{-}Me}$).

^{11}B{^{1}H} NMR (160 MHz, 298 K, acetone-d$_6$) δ/ppm –6.5 (s).

^{31}P{^{1}H} NMR (202 MHz, 298 K, acetone-d$_6$) δ/ppm –13.3 (broad, FWHM ≈ 240 Hz).

ESI(+)-MS (DCM/MeOH, m/z): 641.66 [M–BPh_4–(Me_2bpy)]$^+$ (calc. 641.12), 825.66 [M–BPh_4]$^+$ (base peak, calc. 826.42).

ESI(–)-MS (DCM/MeOH, m/z): 318.88 [BPh_4]$^-$ (calc. 319.23).

Found: C 78.10, H 5.72, N 2.69; $C_{75}H_{64}BCuN_2OP_2$ requires C 78.63, H 5.63, N 2.45.

Please do not adjust margins

Please do not adjust margins

[Cu(POP)(Mebpy)][BAr$^F_4$]

The reagents were [Cu(POP)(Mebpy)][PF_6] (257 mg, 0.280 mmol) and NaBAr$^F_4$ (332 mg, 0.375 mmol). [Cu(POP)(Mebpy)][BAr$^F_4$] was isolated as a yellow solid (381 mg, 0.232 mmol, 83%).

^{1}H NMR (500 MHz, acetone-d$_6$) δ/ppm 8.68 (d, $^2J_{HH}$ = 5.1 Hz, 1H, H^{A6}), 8.50 (d, $^2J_{HH}$ = 8.1 Hz, 1H, H^{A3}), 8.41 (d, $^2J_{HH}$ = 7.9 Hz, 1H, H^{B3}), 8.11 (m, 1H, H^{B4}), 8.07 (m, 1H, H^{A4}), 7.79 (m, 8H, H^{E2}), 7.67 (s, 4H, H^{E4}), 7.47 (d, $^2J_{HH}$ = 7.7 Hz, 1H, H^{B5}), 7.43 (m, 2H, H^{D4}), 7.26 (m, 1H, H^{A5}), 7.24 (m, 2H, H^{C5}), 7.35 (m, 2H, H$^{D4'}$), 7.27 (m, 4H, H^{D3}), 7.27 (m, 4H, H$^{D3'}$), 7.18 (m, 2H, H^{C6}), 7.15 (m, 2H, H^{C4}), 7.14 (m, 4H, H^{D2}), 7.09 (m, 4H, H$^{D2'}$), 6.90 (m, 2H, H^{C3}), 2.35 (s, 3H, H^{B6-Me}).

^{13}C{^{1}H} NMR (126 MHz, acetone-d$_6$) δ/ppm 162.2 (m, C^{E1}), 159.1 (s, C^{B6}), 158.0 (m, C^{C1}), 152.6 (s, C^{A2}), 152.5 (s, C^{A6}), 151.9 (s, C^{B2}), 139.0 (s, C^{B4}), 138.8 (s, C^{A4}), 134.6 (m, C^{E2}), 134.1 (s, C^{C3}), 133.1 (m, C^{D2}), 132.9 (m, C$^{D2'}$), 132.3 (s, C^{D4}), 130.1 (s, C$^{D4'}$), 130.1 (s, C^{C5}), 129.8 (m, C^{D1}+C$^{D1'}$), 128.9 (pseudo-t, $^3J_{CP}$ = 4.6 Hz, C^{D3}+C$^{D3'}$), 128.0 (m, C^{E3}) 126.2 (s, C^{B5}), 125.8 (s, C^{A5}), 125.2 (m, C^{C4}), 124.7 (q, $^1J_{CF}$ = 271.3 Hz, C^{E3-CF3}), 124.2 (m, C^{C2}), 122.7 (s, C^{A3}), 120.3 (m, C^{C6}), 120.0 (s, C^{B3}), 117.5 (m, C^{E4}), 25.5 (s, C^{B6-Me}).

^{11}B{^{1}H} NMR (160 MHz, 298 K, acetone-d$_6$) δ/ppm –6.6 (s).

^{19}F{^{1}H} NMR (202 MHz, 298 K, acetone-d$_6$) δ/ppm –63.3 (s).

^{31}P{^{1}H} NMR (202 MHz, 298 K, acetone-d$_6$) δ/ppm –12.9 (broad, FWHM ≈ 280 Hz).

ESI(+)-MS (DCM/MeOH, m/z): 601.03 [M–BAr$^F_4$–(Mebpy)]$^+$ (calc. 601.09), 771.12 [M–BAr$^F_4$]$^+$ (base peak, calc. 771.17).

ESI(–)-MS (DCM/MeOH, m/z): 862.00 [BAr$^F_4$]$^-$ (calc. 862.06).

Found: C 58.55, H 4.33, N 1.88; $C_{79}H_{49}BCuF_{24}N_2OP_2$ requires C 58.05, H 3.02, N 1.71.

[Cu(POP)(Me$_2$bpy)][BAr$^F_4$]

The reagents were [Cu(POP)(Me$_2$bpy)][PF_6] (259 mg, 0.277 mmol) and NaBAr$^F_4$ (331 mg, 0.374 mmol). [Cu(POP)(Me$_2$bpy)][BAr$^F_4$] was isolated as a yellow solid (385 mg, 0.234 mmol, 84%).

^{1}H NMR (500 MHz, acetone-d$_6$) δ/ppm 8.23 (d, $^2J_{HH}$ = 8.0, 2H, H^{B3}), 8.02 (m, 2H, H^{B4}), 7.79 (m, 8H, H^{E2}), 7.67 (s, 4H, H^{E4}), 7.44 (m, 2H, H^{C5}), 7.44 (m, 2H, H^{B5}), 7.35 (m, 4H, H^{D4}), 7.31 (m, 2H, H^{C4}), 7.29 (m, 2H, H^{C3}), 7.20 (m, 8H, H^{D3}), 7.11 (m, 8H, H^{D2}), 7.06 (m, 2H, H^{C6}), 2.32 (s, H^{B6-Me}).

^{13}C{^{1}H} NMR (126 MHz, acetone-d$_6$) δ/ppm 162.2 (m, C^{E1}), 158.7 (s, C^{B6}), 158.2 (pseudo-t, $^2J_{CP}$ = 6.0 Hz, C^{C1}), 152.5 (s, C^{B2}), 139.0 (s, C^{B4}), 134.6 (m, C^{E2}), 133.7 (s, C^{C3}), 133.0 (pseudo-t, $^2J_{CP}$ = 7.8 Hz, C^{D2}), 132.5 (s, C^{C5}), 131.9 (pseudo-t, $^1J_{CP}$ = 16.3 Hz, C^{C2}), 129.9 (s, C^{D4}), 129.1 (qq, $^2J_{CF}$ = 31.6 Hz, $^4J_{CF}$ = 5.8 Hz, C^{E3}), 128.7 (pseudo-t, $^3J_{CP}$ = 4.6 Hz, C^{D3}), 126.2 (s, C^{B5}), 125.4 (m, C^{D1}), 125.3 (pseudo-t, $^3J_{CP}$ = 2.2 Hz, C^{C4}), 124.5 (q, $^1J_{CF}$ = 272.3 Hz, C^{E3-CF3}), 120.2 (pseudo-t, $^3J_{CP}$ = 1.9 Hz, C^{C6}), 120.0 (s, C^{B3}), 117.6 (m, C^{E4}), 26.6 (s, C^{B6-Me}).

^{11}B{^{1}H} NMR (160 MHz, 298 K, acetone-d$_6$) δ/ppm –6.5 (s).

^{19}F{^{1}H} NMR (202 MHz, 298 K, acetone-d$_6$) δ/ppm –63.3 (s).

^{31}P{^{1}H} NMR (202 MHz, 298 K, acetone-d$_6$) δ/ppm –13.5 (broad, FWHM ≈ 280 Hz).

ESI(+)-MS (DCM/MeOH, m/z): 601.00 [M–BAr$^F_4$–(Me$_2$bpy)]$^+$ (calc. 601.09), 785.11 [M–BAr$^F_4$]$^+$ (base peak, calc. 785.19).

ESI(–)-MS (DCM/MeOH, m/z): 861.00 [BAr$^F_4$]$^-$ (calc. 862.06).

Found: C 58.23, H 4.13, N 1.82; $C_{80}H_{51}BCuF_{24}N_2OP_2$ requires C 58.29, H 3.12, N 1.70.

[Cu(xantphos)(Mebpy)][BAr$^F_4$]

The reagents were [Cu(xantphos)(Mebpy)][PF_6] (271 mg, 0.283 mmol) and NaBAr$^F_4$ (332 mg, 0.375 mmol). [Cu(xantphos)(Mebpy)][BAr$^F_4$] was isolated as a yellow solid (385 mg, 0.229 mmol, 81%).

^{1}H NMR (500 MHz, acetone-d$_6$) δ/ppm 8.54 (m, 1H, H^{A3}), 8.53 (m, 1H, H^{A6}), 8.45 (d, $^2J_{HH}$ = 7.9 Hz, 1H, H^{B3}), 8.15 (m, 1H, H^{B4}), 8.10 (m, 1H, H^{A4}), 7.85 (dd, $^2J_{HH}$ = 7.8 Hz, $^3J_{HH}$ = 1.4 Hz, 2H, H^{C5}), 7.79 (m, 8H, H^{E2}), 7.67 (s, 4H, H^{E4}), 7.52 (d, $^2J_{HH}$ = 8.0 Hz, 1H, H^{A5}), 7.49 (m, 1H, H^{B5}), 7.38 (m, 2H, H^{D4}), 7.34 (m, 2H, H$^{D4'}$), 7.30 (d, $^2J_{HH}$ = 7.7 Hz, 2H, H^{C4}), 7.26 (m, 4H, H$^{D3'}$), 7.19 (m, 4H, H^{D3}), 7.16 (m, 4H, H$^{D2'}$), 6.92 (m, 4H, H^{D2}), 6.66 (m, 2H, H^{C3}), 2.03 (m, 3H, H^{B6-Me}), 1.89 (m, 3H, H$^{xantphos-Me'}$), 1.69 (m, 3H, H$^{xantphos-Me}$).

^{13}C{^{1}H} NMR (126 MHz, acetone-d$_6$) δ/ppm 161.7 (q, $^1J_{CB}$ = 49.7 Hz, C^{E1}), 158.6 (s, C^{B6}), 154.9 (pseudo-t, $^2J_{CP}$ = 6.2 Hz, C^{C1}), 152.5 (s, C^{A2}), 151.5 (s, C^{B2}), 149.1 (s, C^{A6}), 139.4 (s, C^{B4}), 138.9 (s, C^{A4}), 134.6 (m, C^{E2}), 134.1 (s, C^{C6}), 133.0 (pseudo-t, $^2J_{CP}$ = 8.0 Hz, C^{D2}), 132.6 (pseudo-t, $^2J_{CP}$ = 7.9 Hz, C$^{D2'}$), 131.7 (pseudo-t, $^1J_{CP}$ = 16.5 Hz, C$^{D1'}$), 131.6 (pseudo-t, $^1J_{CP}$ = 17.7 Hz, C^{D1}), 130.6 (s, C^{C3}), 130.2 (s, C$^{D4'}$), 130.1 (s, C^{D4}), 129.0 (pseudo-t, $^3J_{CP}$ = 4.7 Hz, C$^{D3'}$), 128.8 (pseudo-t, $^3J_{CP}$ = 4.7 Hz, C^{D3}), 127.7 (m, C^{E3}), 127.6 (s, C^{C5}), 126.2 (s, C^{B5}), 126.1 (s, C^{A5}), 125.4 (pseudo-t, $^3J_{CP}$ = 2.5 Hz, C^{C4}), 124.5 (q, $^1J_{CF}$ = 271.8 Hz, C^{E3-CF3}), 122.9 (s, C^{A3}), 120.7 (pseudo-t, $^1J_{CP}$ = 12.9 Hz, C^{C2}), 120.2 (s, C^{B3}), 117.6 (m, C^{E4}), 36.0 (s, C$^{xantphos-bridge}$), 29.4 (s, C$^{xantphos-Me}$), 25.5 (s, C$^{xantphos-Me'}$), 25.3 (s, C^{B6-Me}).

^{11}B{^{1}H} NMR (160 MHz, 298 K, acetone-d$_6$) δ/ppm –6.6 (s).

^{19}F{^{1}H} NMR (202 MHz, 298 K, acetone-d$_6$) δ/ppm –63.3 (s).

^{31}P{^{1}H} NMR (202 MHz, 298 K, acetone-d$_6$) δ/ppm –13.0 (broad, FWHM ≈ 270 Hz).

ESI(+)-MS (DCM/MeOH, m/z): 641.06 [M–BAr$^F_4$–(Mebpy)]$^+$ (calc. 641.12), 811.11 [M–BAr$^F_4$]$^+$ (base peak, calc. 811.21).

ESI(–)-MS (DCM/MeOH, m/z): 862.0 [BAr$^F_4$]$^-$ (calc. 862.06).

Found: C 58.66, H 3.21, N 1.73; $C_{82}H_{53}BCuF_{24}N_2OP_2$ requires C 58.81, H 3.19, N 1.67.

This journal is © The Royal Society of Chemistry 20xx

Please do not adjust margins

Please do not adjust margins

[Cu(xantphos)(Me_2bpy)][BAr^F_4]

The reagents were [Cu(xantphos)(Mebpy)][PF_6] (272 mg, 0.280 mmol) and NaBAr$^F_4$ (333 mg, 0.376 mmol). [Cu(xantphos)(Me_2bpy)][BAr^F_4] was isolated as a yellow solid (360 mg, 0.213 mmol, 76%).

^{1}H NMR (500 MHz, acetone-d$_6$) δ/ppm 8.19 (d, $^2J_{HH}$ = 8.0 Hz, 2H, H^{B3}), 7.95 (m, 2H, H^{B4}), 7.83 (dd, $^2J_{HH}$ = 7.8, $^3J_{HH}$ = 1.4 Hz, 2H, H^{C5}), 7.79 (m, 8H, H^{E2}), 7.67 (s, 4H, H^{E4}), 7.40 (m, 4H, H^{D4}), 7.39 (m, 2H, H^{B5}), 7.31 (m, 2H, H^{C4}), 7.23 (m, 8H, H^{D3}), 7.17 (m, 8H, H^{D2}), 6.95 (m, 2H, H^{C3}), 2.14 (s, 6H, H$^{6\text{-}Me}$), 1.76 (s, 6H, H$^{xantphos\text{-}Me}$).

^{13}C{^{1}H} NMR (126 MHz, acetone-d$_6$) δ/ppm 161.9 (m, C^{E1}), 158.3 (s, C^{B6}), 154.9 (m, C^{C1}), 152.2 (s, C^{B2}), 139.0 (s, C^{B4}), 133.9 (s, C^{C6}), 134.6 (m, C^{E2}), 133.1 (pseudo-t, $^2J_{CP}$ = 7.6 Hz, C^{D2}), 131.50 (pseudo-t, $^2J_{CP}$ = 16.4 Hz, C^{D1}), 130.2 (s, C^{C3}), 130.1 (s, C^{D4}), 128.2 (pseudo-t, $^3J_{CP}$ = 4.5 Hz, C^{D3}), 127.8 (s, C^{C5}), 127.7 (m, C^{E3}), 125.8 (s, C^{B5}), 125.5 (pseudo-t, $^3J_{CP}$ = 2.3 Hz, C^{C4}), 124.5 (q, $^1J_{CF}$ = 271.8 Hz, C$^{E3\text{-}CF3}$), 121.8 (m, C^{C2}), 120.1 (s, C^{B3}), 117.5 (m, C^{E4}), 27.72 (s, C$^{xantphos\text{-}bridge}$), 26.8 (s, C$^{xantphos\text{-}Me}$), 26.13 (s, C$^{B6\text{-}Me}$),.

^{11}B{^{1}H} NMR (160 MHz, 298 K, acetone-d$_6$) δ/ppm –6.6 (s).

^{19}F{^{1}H} NMR (202 MHz, 298 K, acetone-d$_6$) δ/ppm –63.3 (s).

^{31}P{^{1}H} NMR (202 MHz, 298 K, acetone-d$_6$) δ/ppm –13.7 (broad, FWHM ≈ 280 Hz).

ESI(+)-MS (DCM/MeOH, m/z): 641.07 [M–BAr^F_4–(Me_2bpy)]$^+$ (calc. 641.12), 825.16 [M–BAr^F_4]$^+$ (base peak, calc. 826.42).

ESI(–)-MS (DCM/MeOH, m/z): 862.00 [BAr^F_4]$^-$ (calc. 862.06).

Found: C 59.12, H 3.56, N 1.75; $C_{83}H_{55}BCuF_{24}N_2OP_2$ requires C 59.04, H 3.28, N 1.66.

Please do not adjust margins

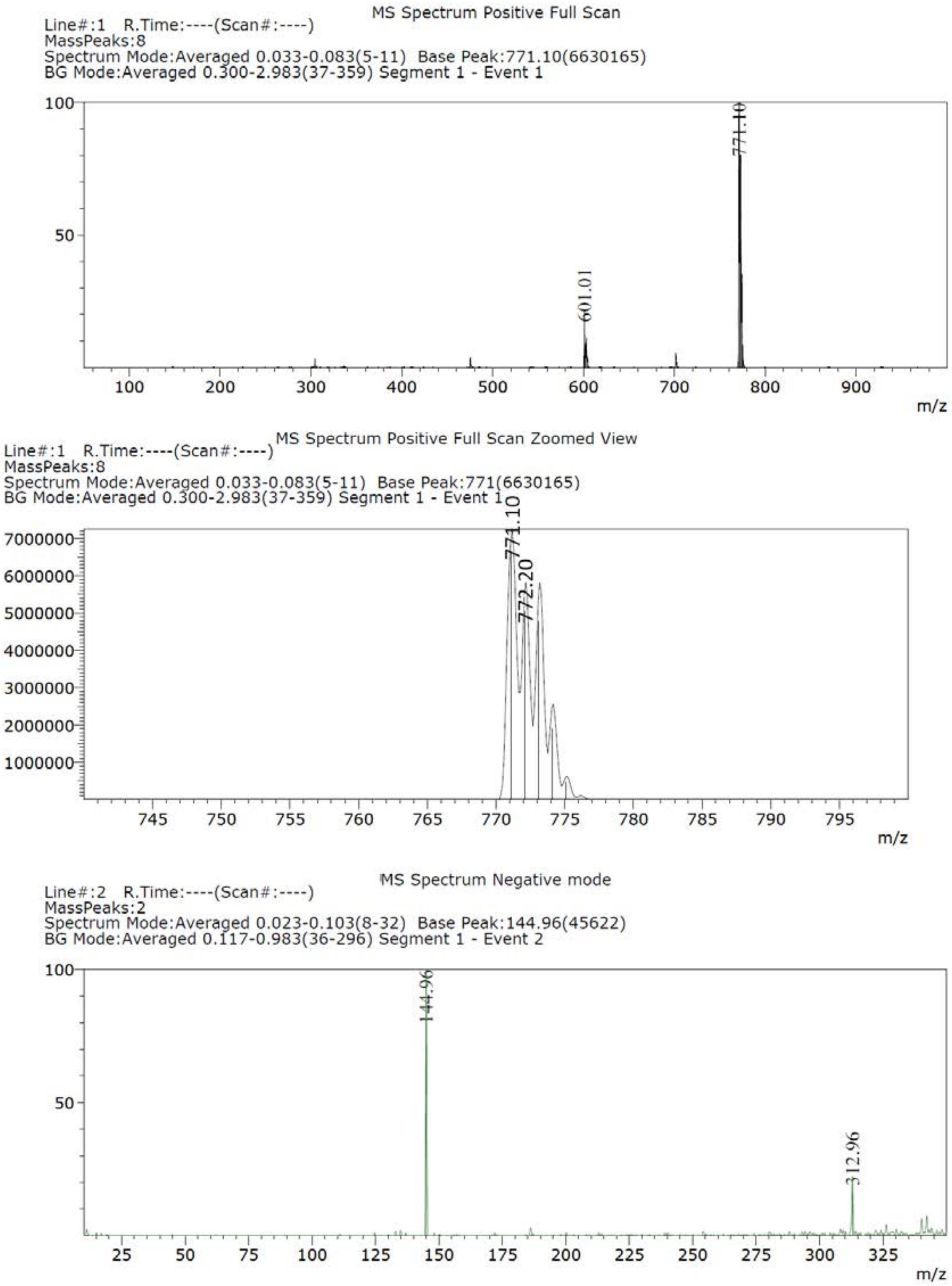

Fig. S1 The ESI mass spectrum (positive and negative mode) of [Cu(POP)(Mebpy)][PF_6].

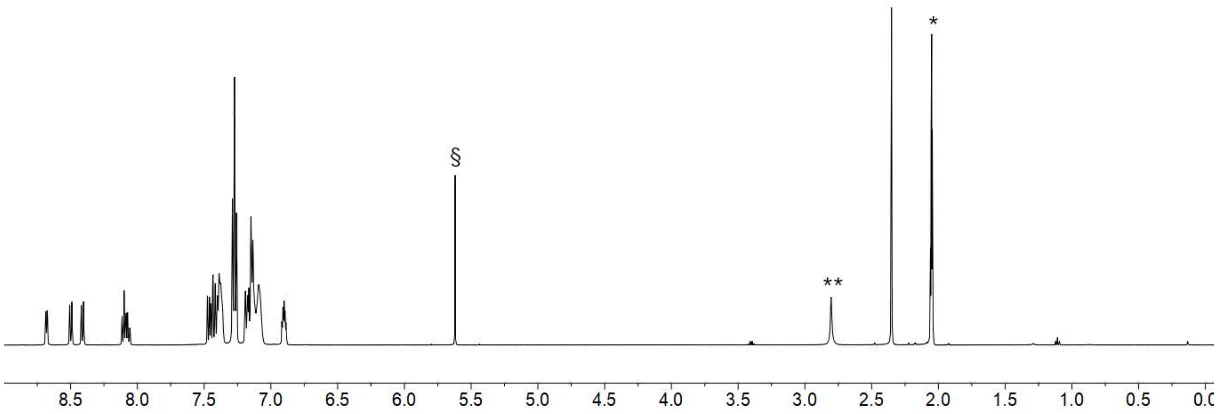

Fig. S2 ^{1}H NMR spectrum of [Cu(POP)(Mebpy)][PF_6] (500 MHz, 298 K, acetone-d_6). * = residual acetone-d_5; ** = H_2O and HDO; § = CH_2Cl_2.

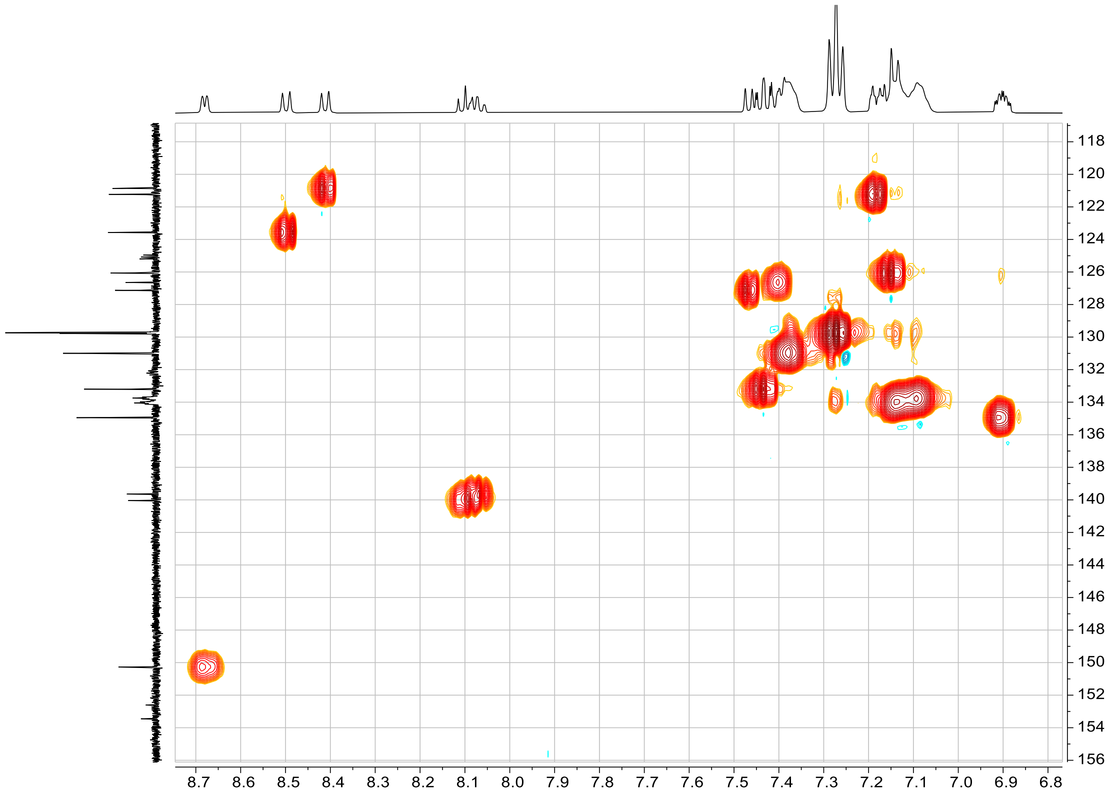

Fig. S3 The aromatic region of the HMQC spectrum (500 MHz ^{1}H, 126 MHz $^{13}C\{^{1}H\}$, acetone-d_6, 298 K) of [Cu(POP)(Mebpy)][PF_6]. Scale: δ / ppm.

This journal is © The Royal Society of Chemistry 20xx

Please do not adjust margins

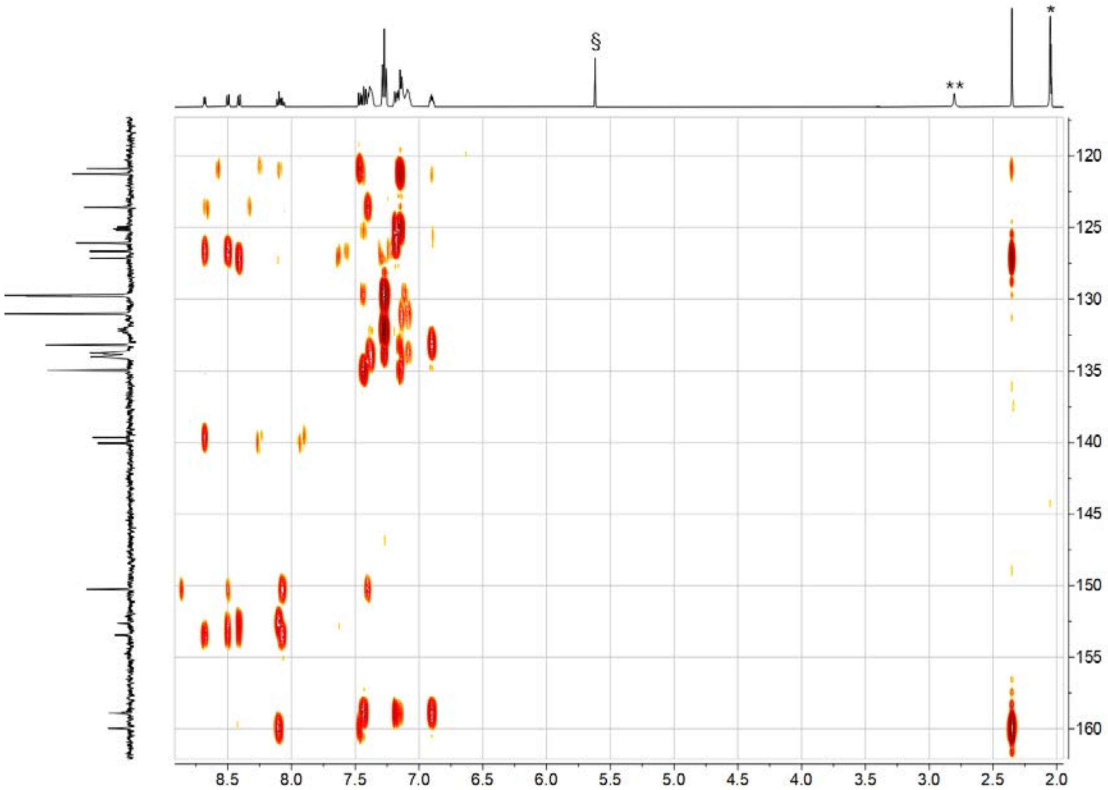

Fig. S4 Part of the HMBC spectrum (500 MHz 1H, 126 MHz $^{13}C\{^1H\}$, acetone-d_6, 298 K) of [Cu(POP)(Mebpy)][PF_6]. Scale: δ / ppm. ** = H_2O and HDO; § = CH_2Cl_2.

 This journal is © The Royal Society of Chemistry 20xx

Please do not adjust margins

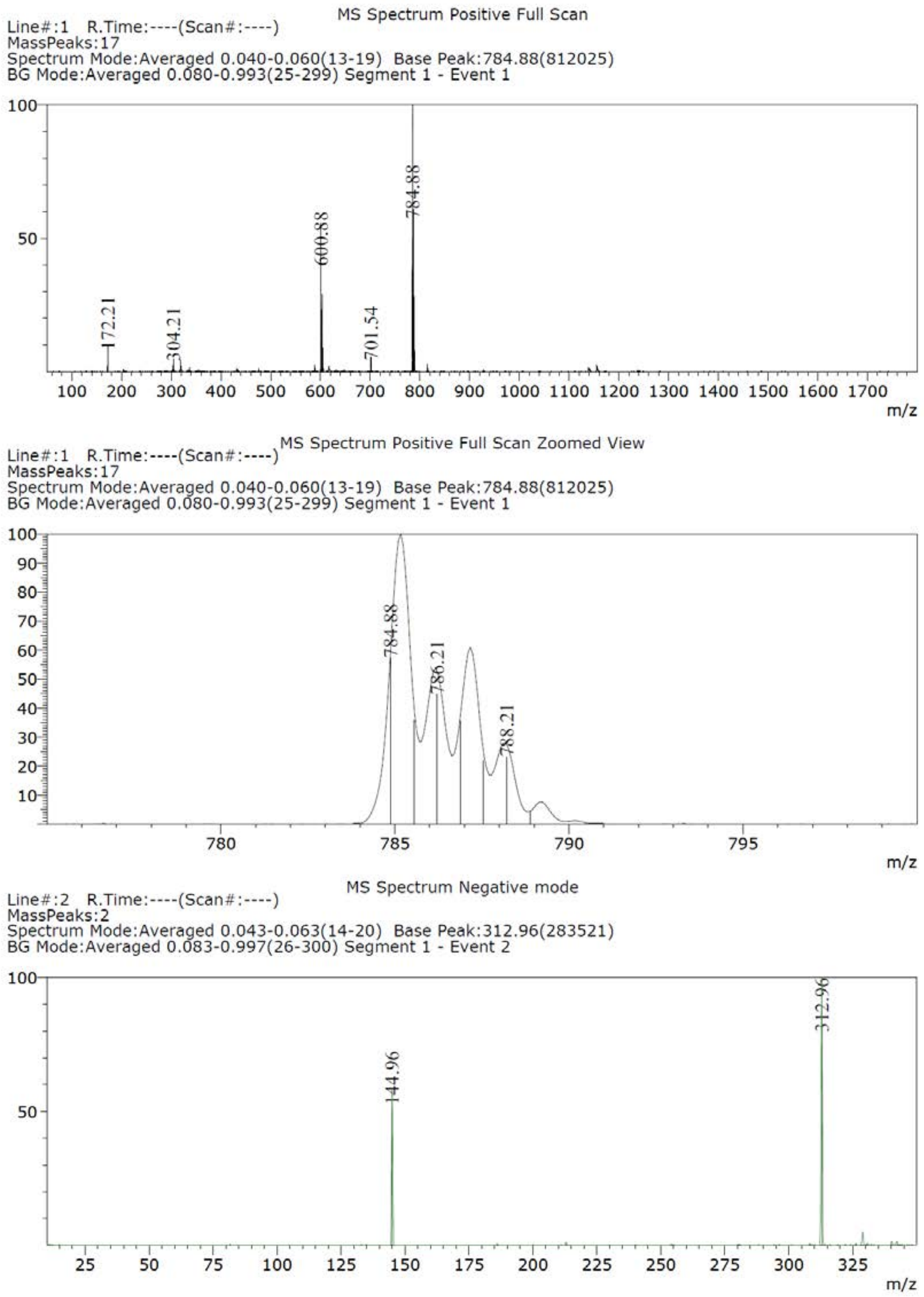

Fig. S5 The ESI mass spectrum (positive and negative mode) of [Cu(POP)(Me_2bpy)][PF_6].

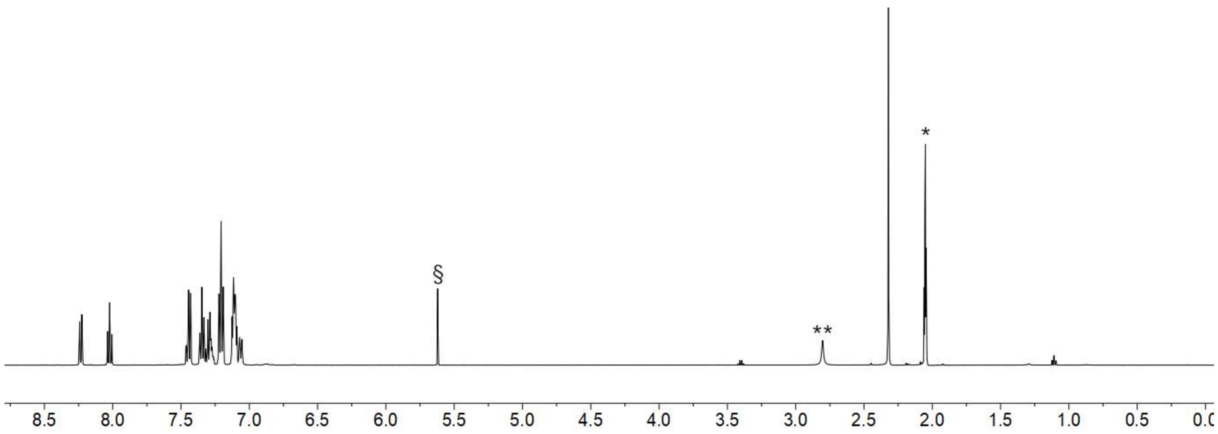

Fig. S6 ^{1}H NMR spectrum of [Cu(POP)(Me_2bpy)][PF_6] (500 MHz, 298 K, acetone-d6). * = residual acetone-*d*$_5$; ** = H_2O and HDO; § = CH_2Cl_2.

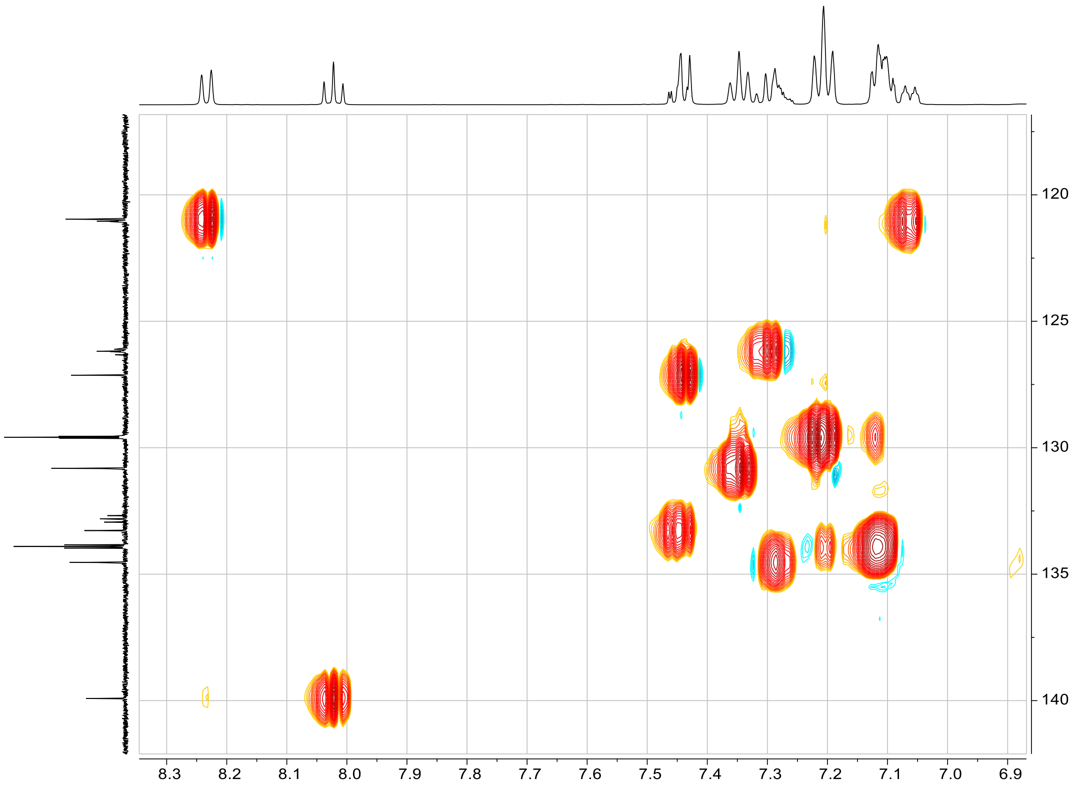

Fig. S7 The aromatic region of the HMQC spectrum (500 MHz ^{1}H, 126 MHz $^{13}C\{^{1}H\}$, acetone-*d*$_6$, 298 K) of [Cu(POP)(Me_2bpy)][PF_6]. Scale: δ / ppm.

This journal is © The Royal Society of Chemistry 20xx

Please do not adjust margins

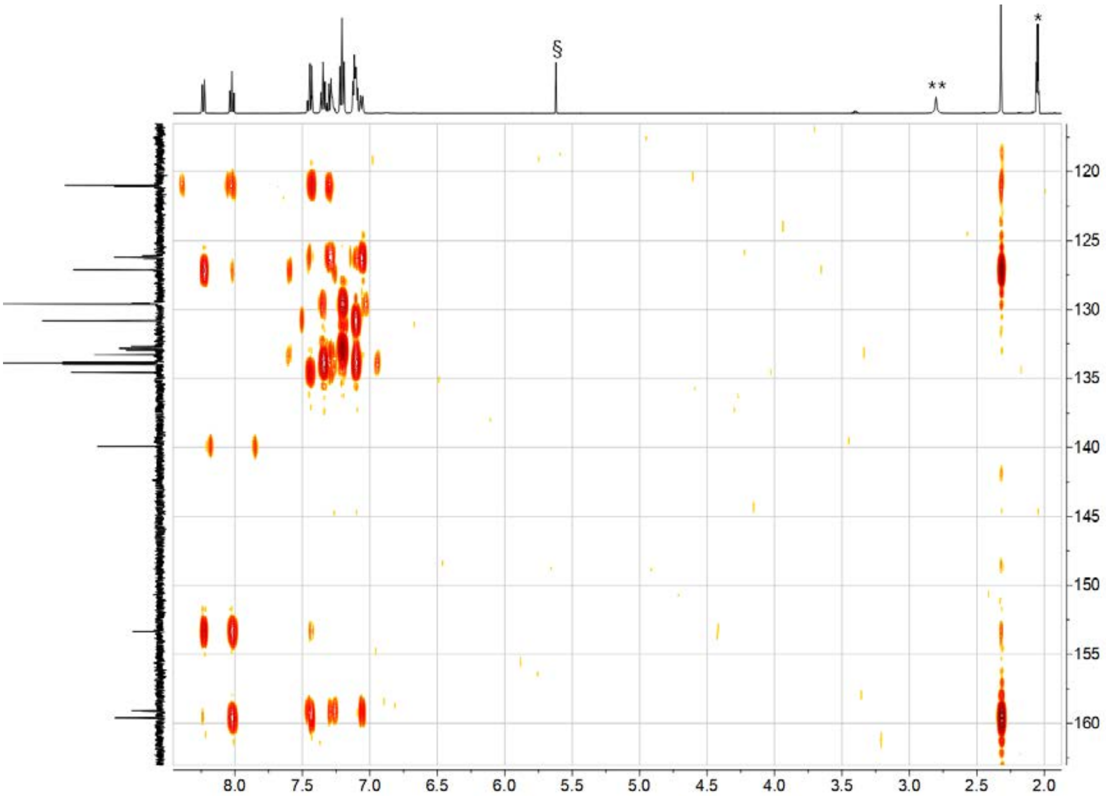

Fig. S8 Part of the HMBC spectrum (500 MHz 1H, 126 MHz $^{13}C\{^1H\}$, acetone-d_6, 298 K) of [Cu(POP)(Me_2bpy)][PF_6]. Scale: δ / ppm. ** = H_2O and HDO; § = CH_2Cl_2.

Please do not adjust margins

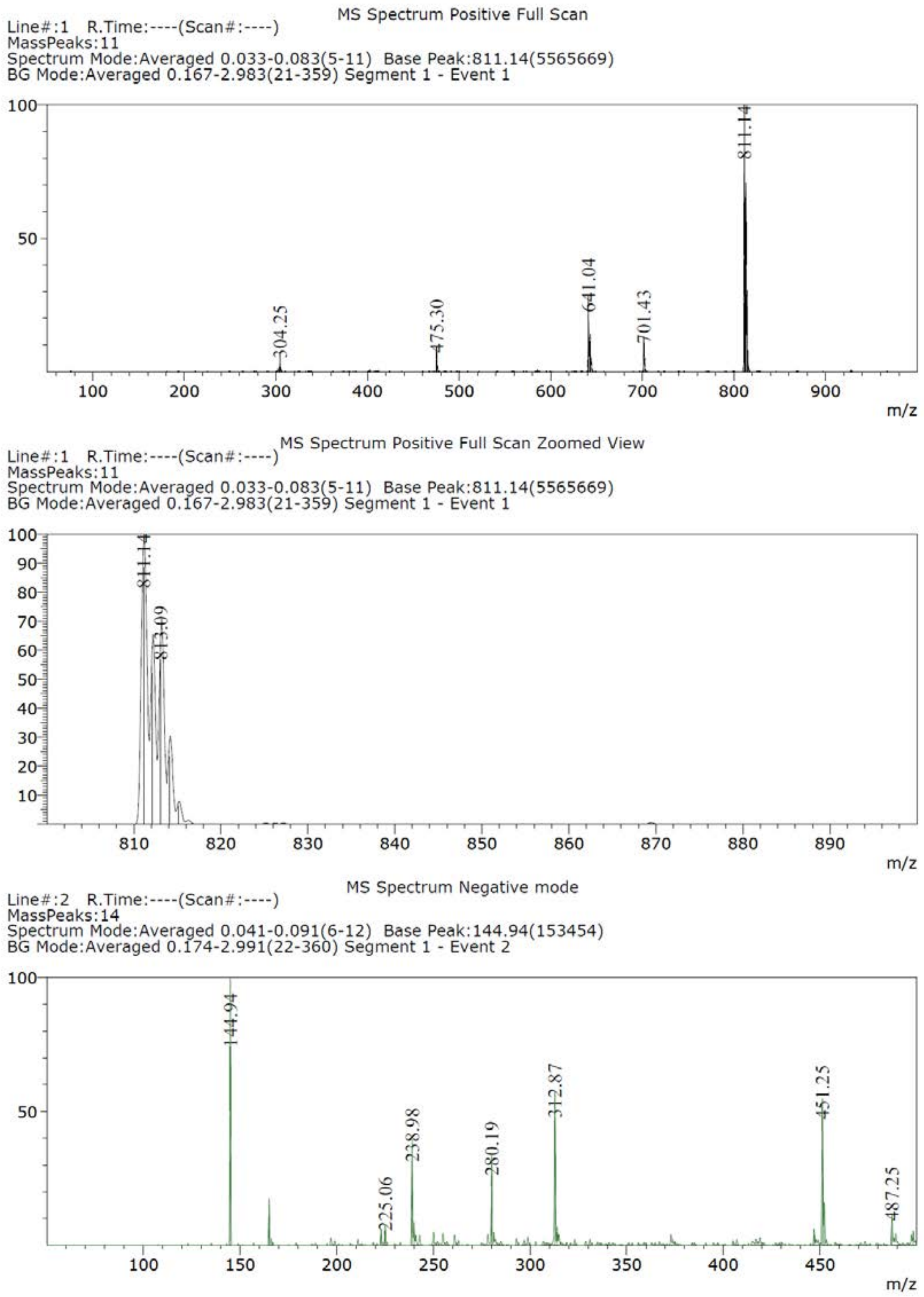

Fig. S9 The ESI mass spectrum (positive and negative mode) of [Cu(xantphos)(Mebpy)][PF_6].

This journal is © The Royal Society of Chemistry 20xx

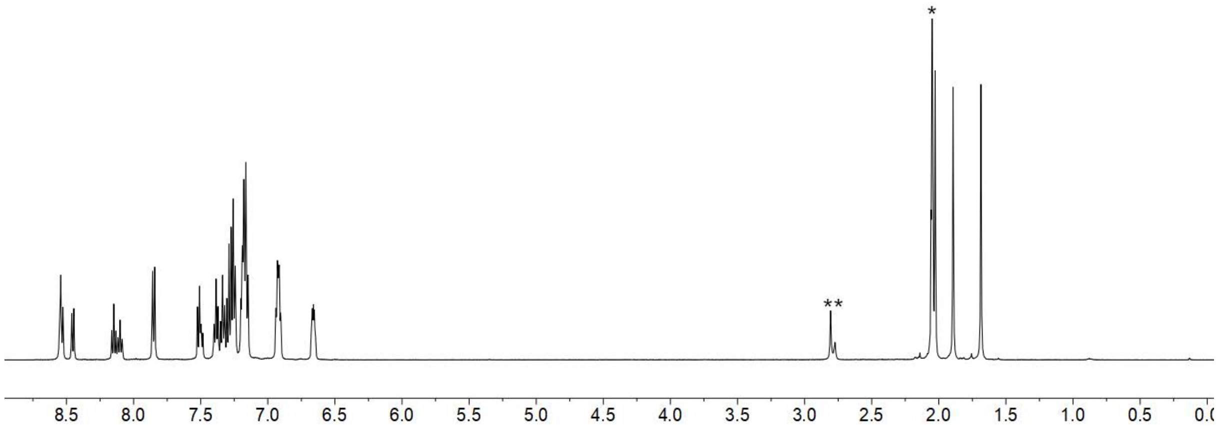

Fig. S10 ^{1}H NMR spectrum of [Cu(xantphos)(Mebpy)][PF_6] (500 MHz, 298 K, acetone-d6). * = residual acetone-d_5; ** = H_2O and HDO.

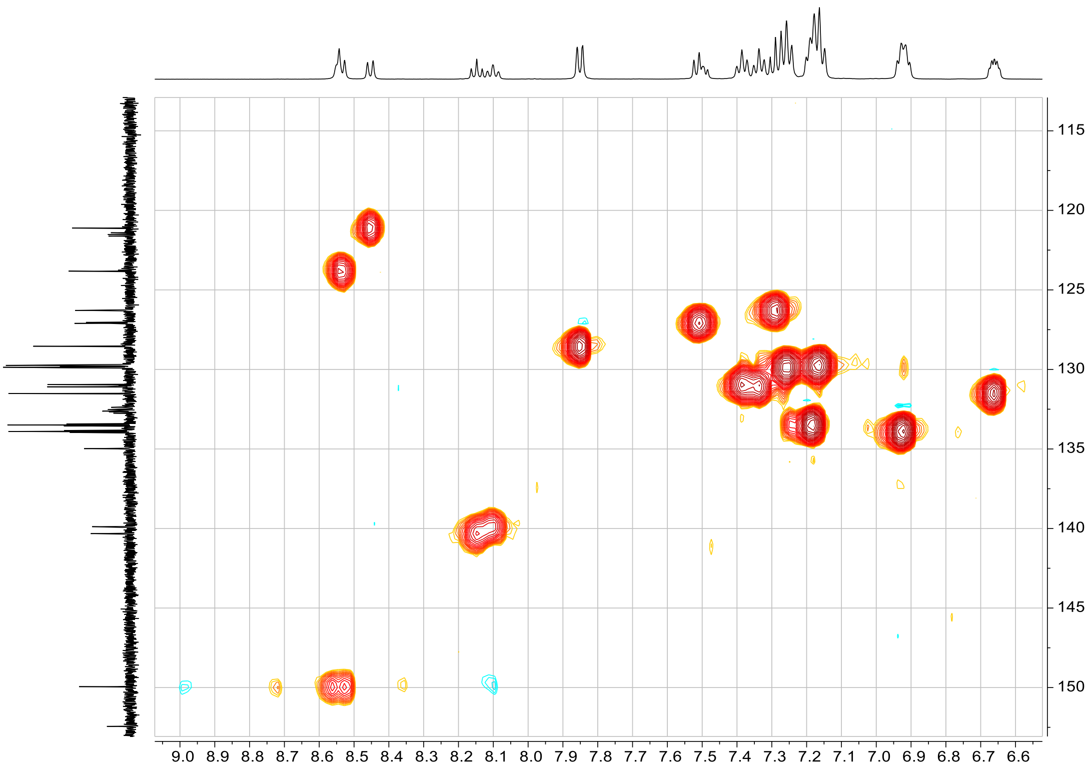

Fig. S11 The aromatic region of the HMQC spectrum (500 MHz ^{1}H, 126 MHz $^{13}C\{^1H\}$, acetone-d_6, 298 K) of [Cu(xantphos)(Mebpy)][PF_6]. Scale: δ / ppm.

Please do not adjust margins

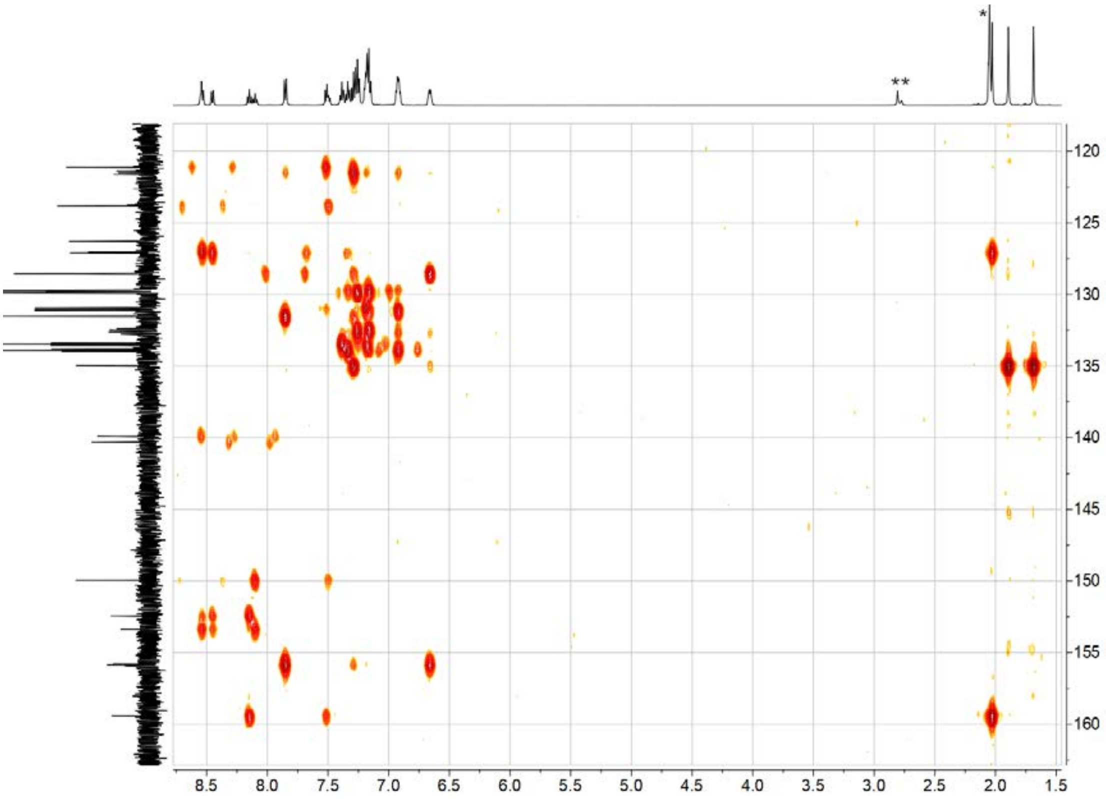

Fig. S12 Part of the HMBC spectrum (500 MHz 1H, 126 MHz $^{13}C\{^{1}H\}$, acetone-d_6, 298 K) of [Cu(xantphos)(Mebpy)][PF_6]. Scale: δ / ppm. ** = H_2O and HDO.

Please do not adjust margins

Please do not adjust margins

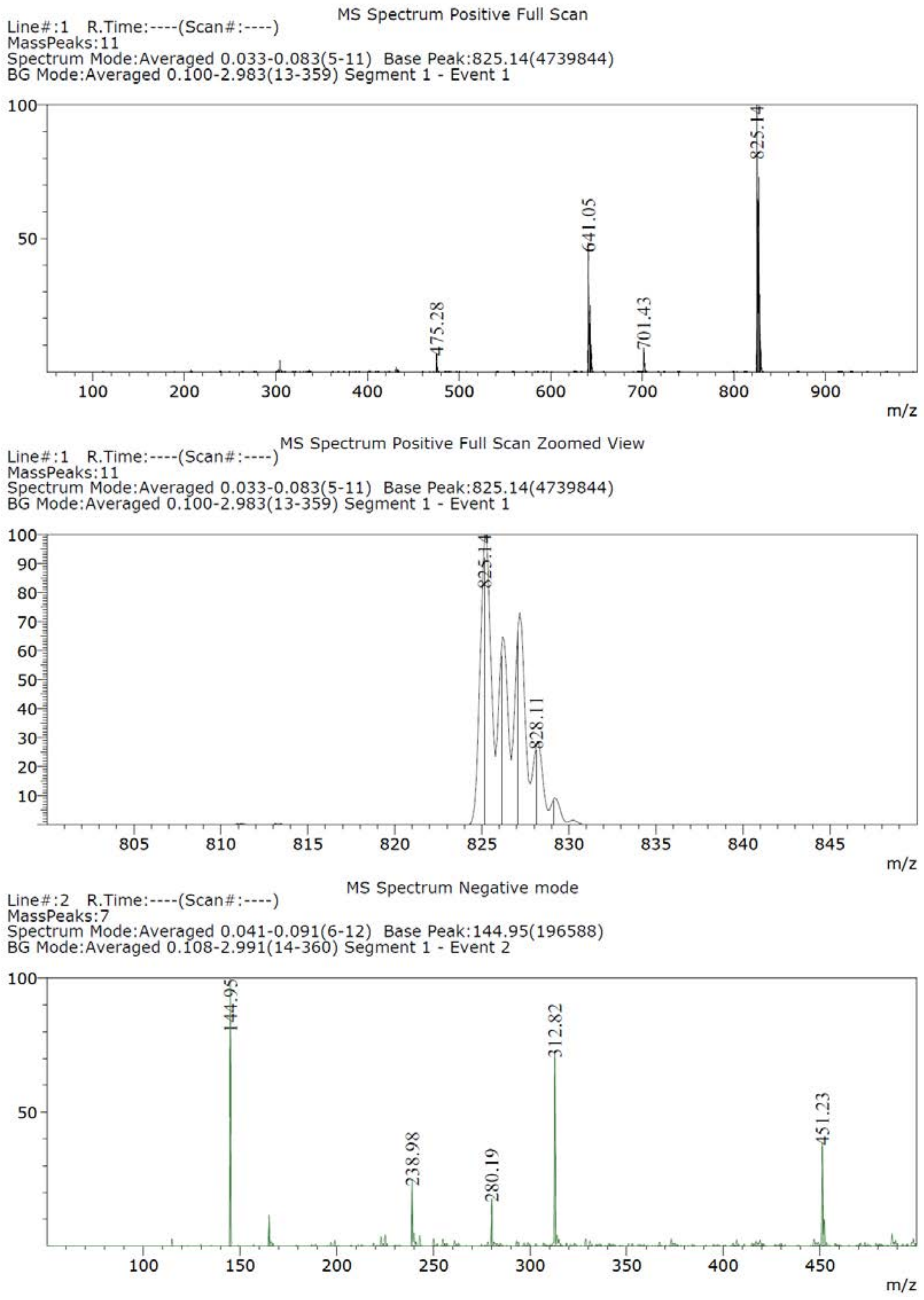

Fig. S13 The ESI mass spectrum (positive and negative mode) of [Cu(xantphos)(Me_2bpy)][PF_6].

Please do not adjust margins

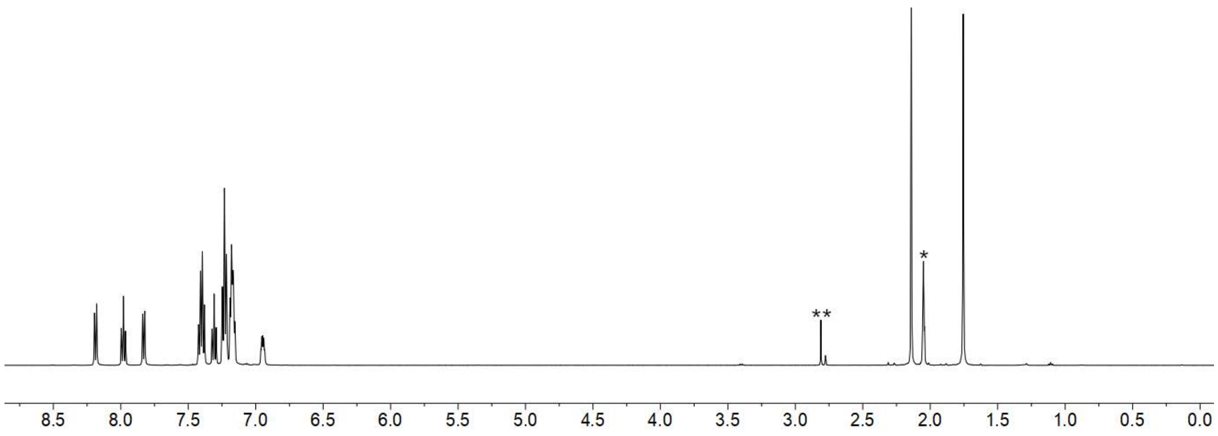

Fig. S14 ^{1}H NMR spectrum of [Cu(xantphos)(Me$_2$bpy)][PF$_6$] (500 MHz, 298 K, acetone-d6). * = residual acetone-d_5; ** = H_2O and HDO.

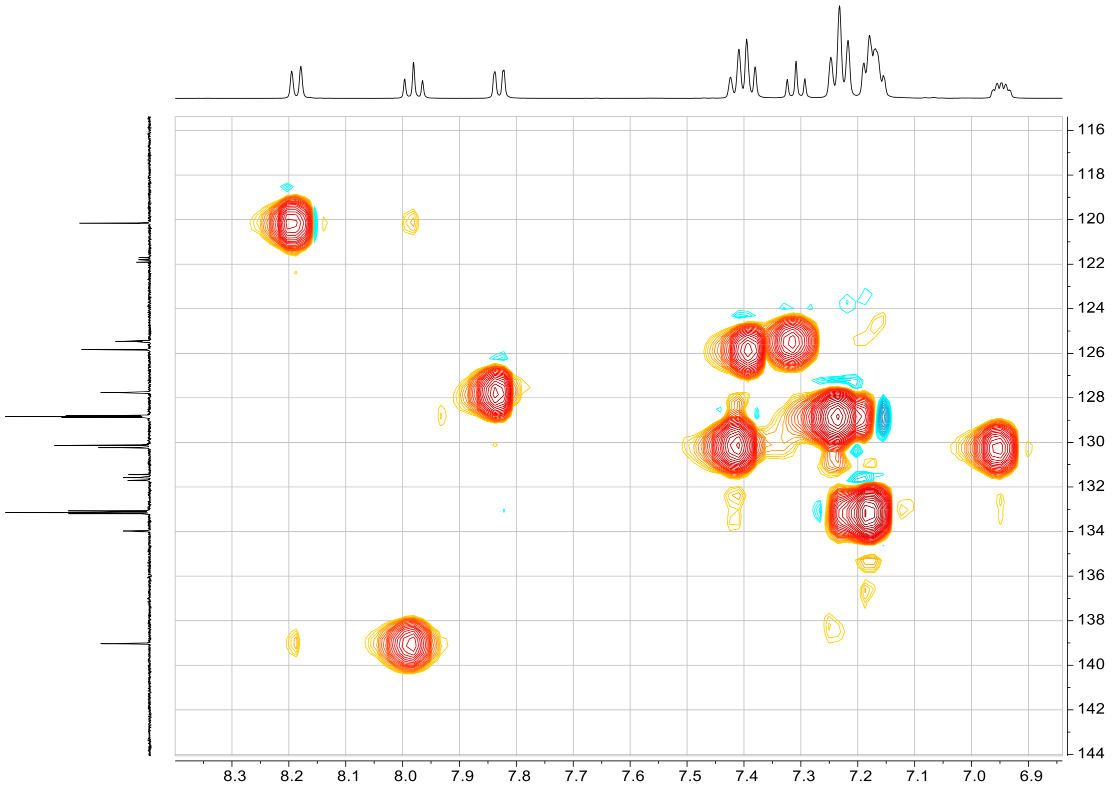

Fig. S15 The aromatic region of the HMQC spectrum (500 MHz ^{1}H, 126 MHz $^{13}C\{^{1}H\}$, acetone-d_6, 298 K) of [Cu(xantphos)(Me$_2$bpy)][PF$_6$]. Scale: δ / ppm.

This journal is © The Royal Society of Chemistry 20xx

Please do not adjust margins

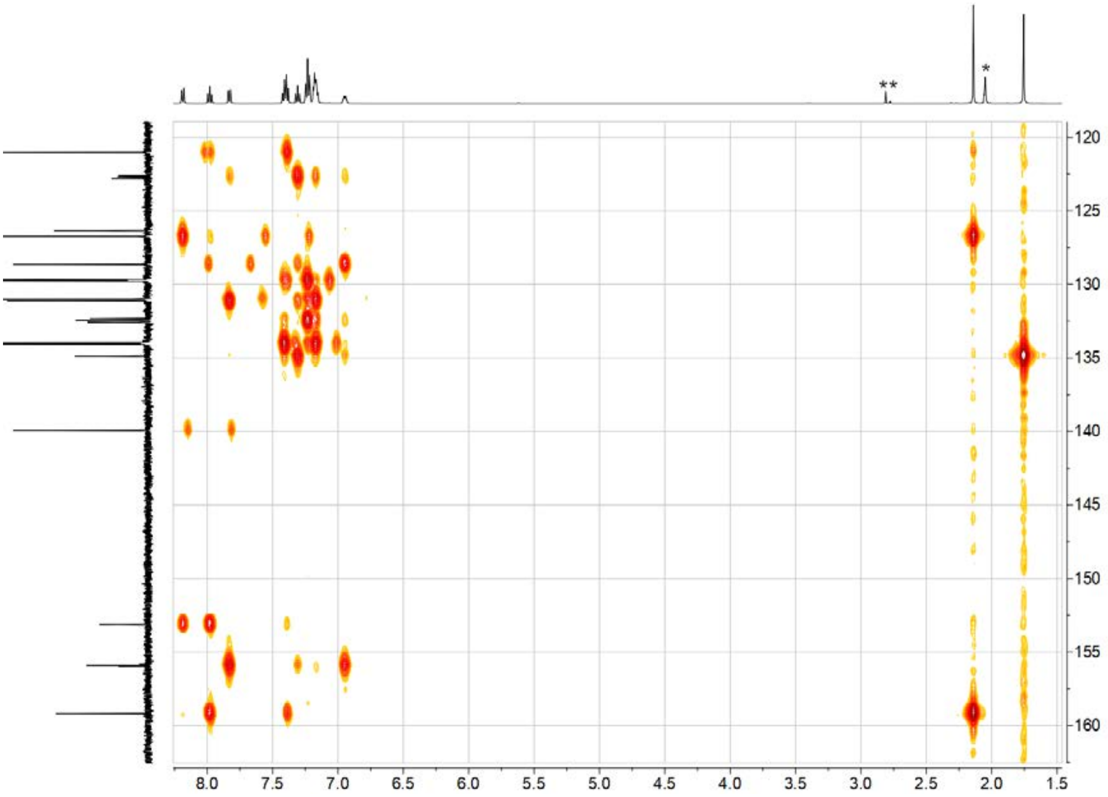

Fig. S16 Part of the HMBC spectrum (500 MHz 1H, 126 MHz $^{13}C\{^{1}H\}$, acetone-d_6, 298 K) of [Cu(xantphos)(Me_2bpy)][PF_6]. Scale: δ / ppm. ** = H_2O and HDO.

Please do not adjust margins

Please do not adjust margins

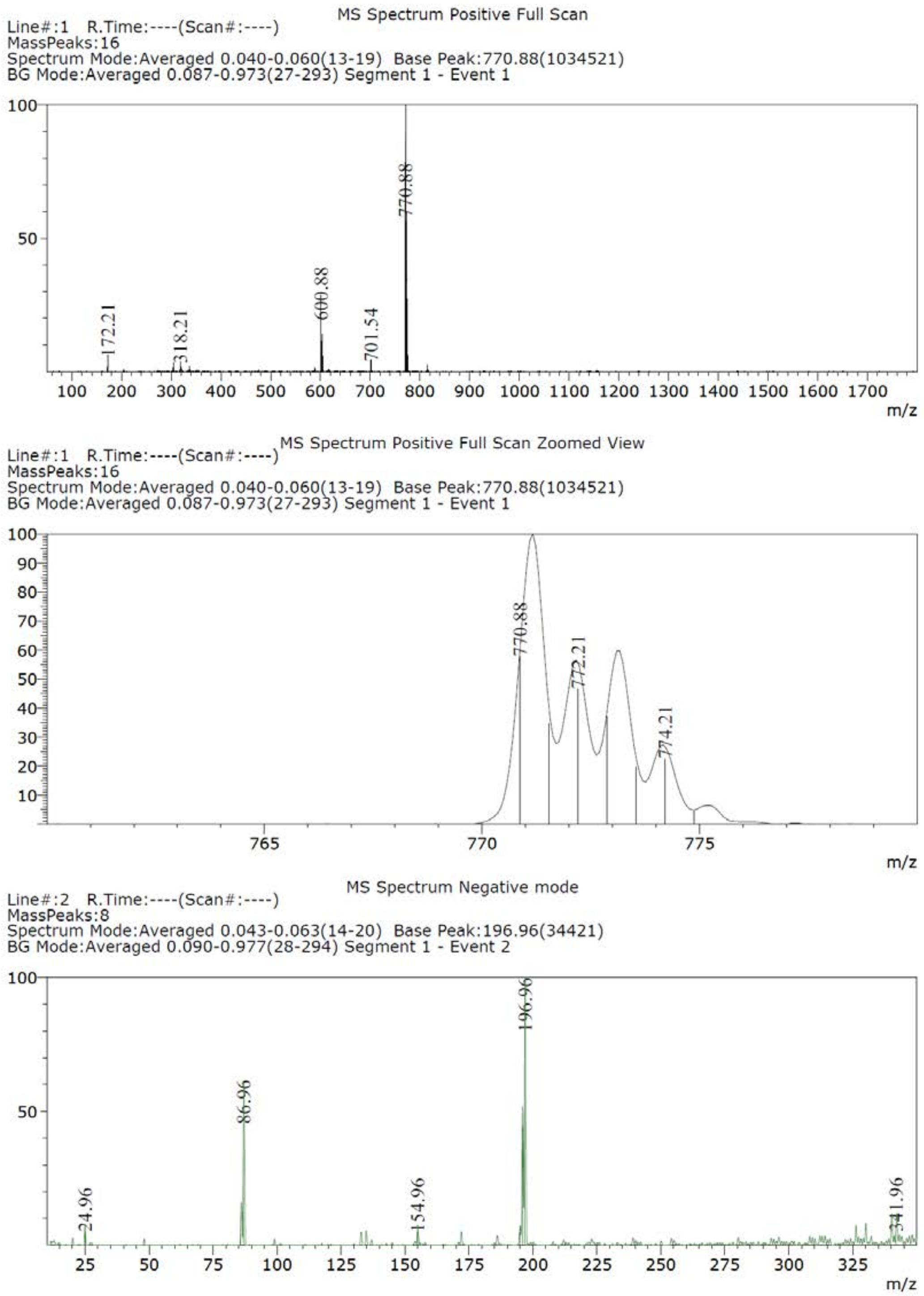

Fig. S17 The ESI mass spectrum (positive and negative mode) of [Cu(POP)(Mebpy)][BF_4].

This journal is © The Royal Society of Chemistry 20xx

Please do not adjust margins

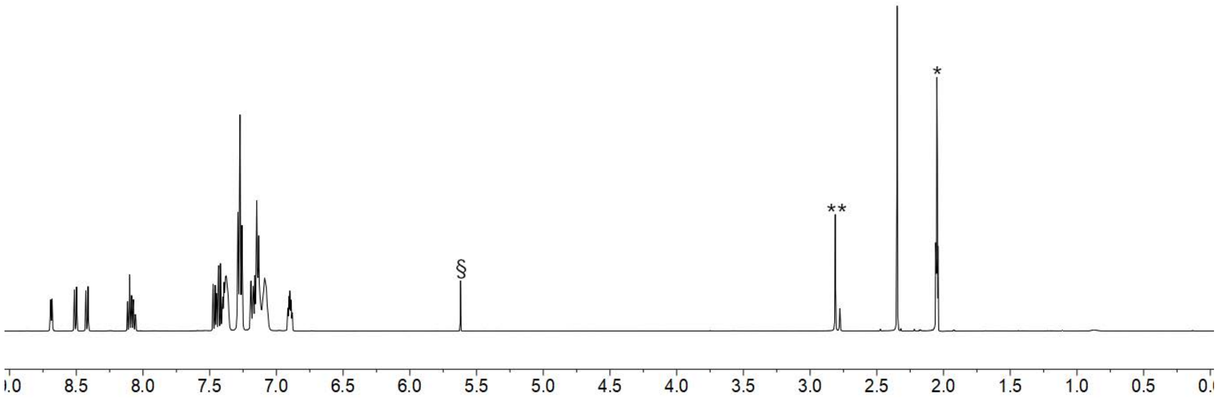

Fig. S18 ^{1}H NMR spectrum of [Cu(POP)(Mebpy)][BF_4] (500 MHz, 298 K, acetone-d6). * = residual acetone-d_5; ** = H_2O and HDO; § = CH_2Cl_2.

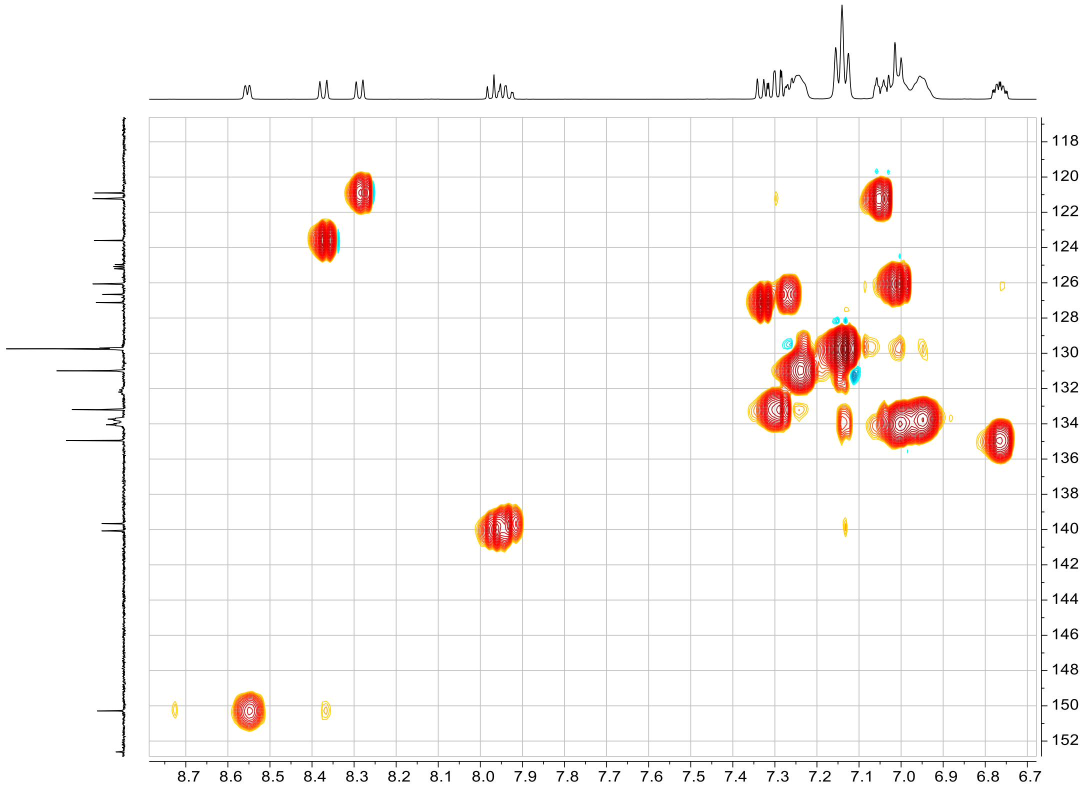

Fig. S19 The aromatic region of the HMQC spectrum (500 MHz ^{1}H, 126 MHz $^{13}C\{^{1}H\}$, acetone-d_6, 298 K) of [Cu(POP)(Mebpy)][BF_4]. Scale: δ / ppm.

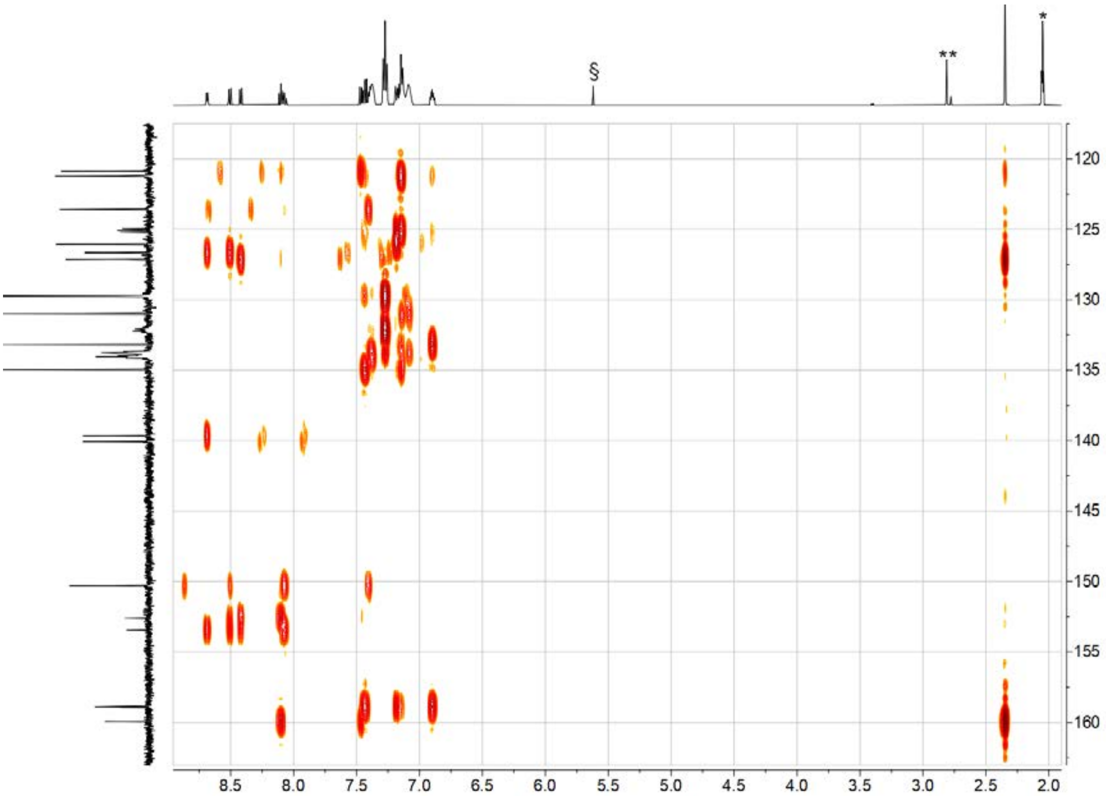

Fig. S20 Part of the HMBC spectrum (500 MHz 1H, 126 MHz $^{13}C\{^{1}H\}$, acetone-d_6, 298 K) of [Cu(POP)(Mebpy)][BF_4]. Scale: δ / ppm. ** = H_2O and HDO; § = CH_2Cl_2.

 This journal is © The Royal Society of Chemistry 20xx

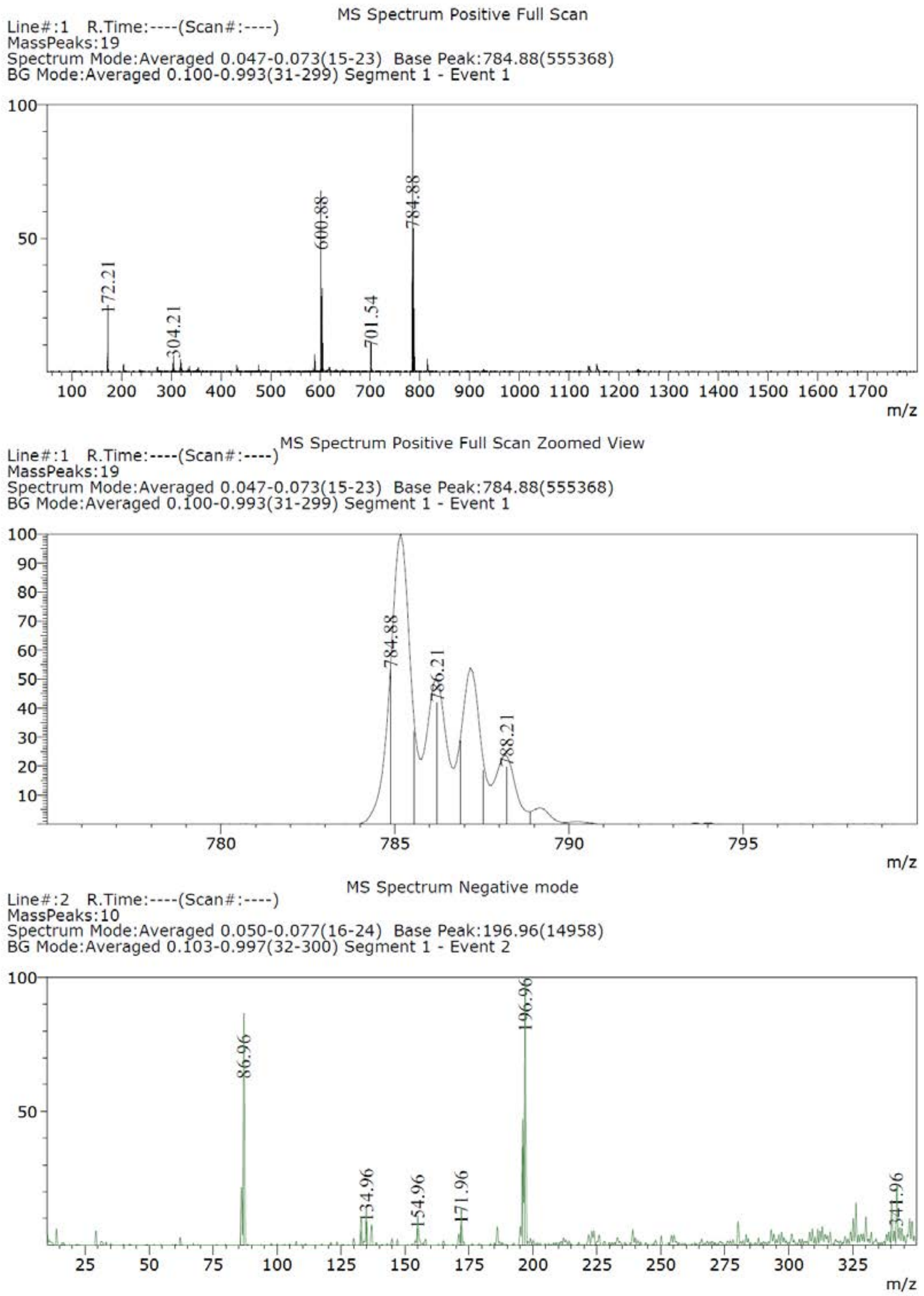

Fig. S21 The ESI mass spectrum (positive and negative mode) of [Cu(POP)(Me_2bpy)][BF_4].

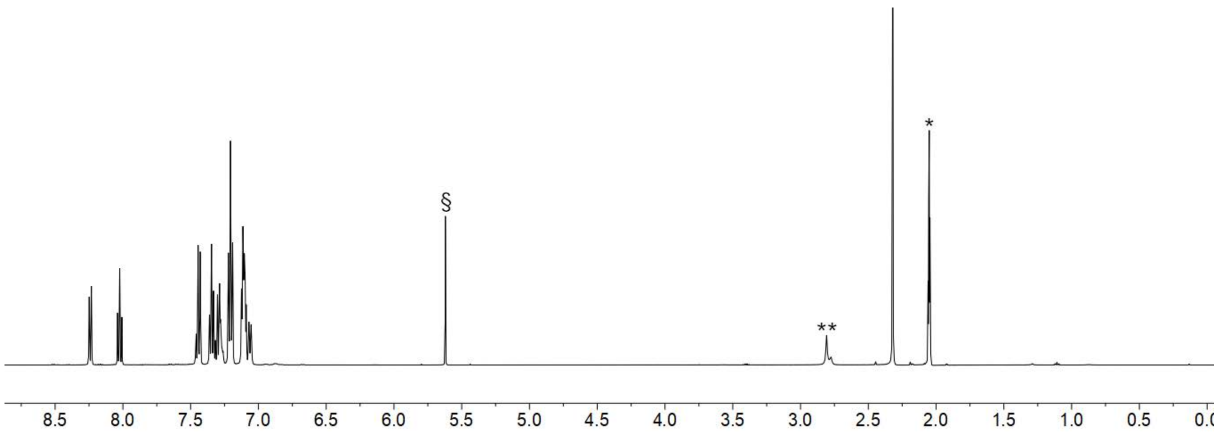

Fig. S22 ^{1}H NMR spectrum of [Cu(POP)(Me_2bpy)][BF_4] (500 MHz, 298 K, acetone-d6). * = residual acetone-d_5; ** = H_2O and HDO; § = CH_2Cl_2.

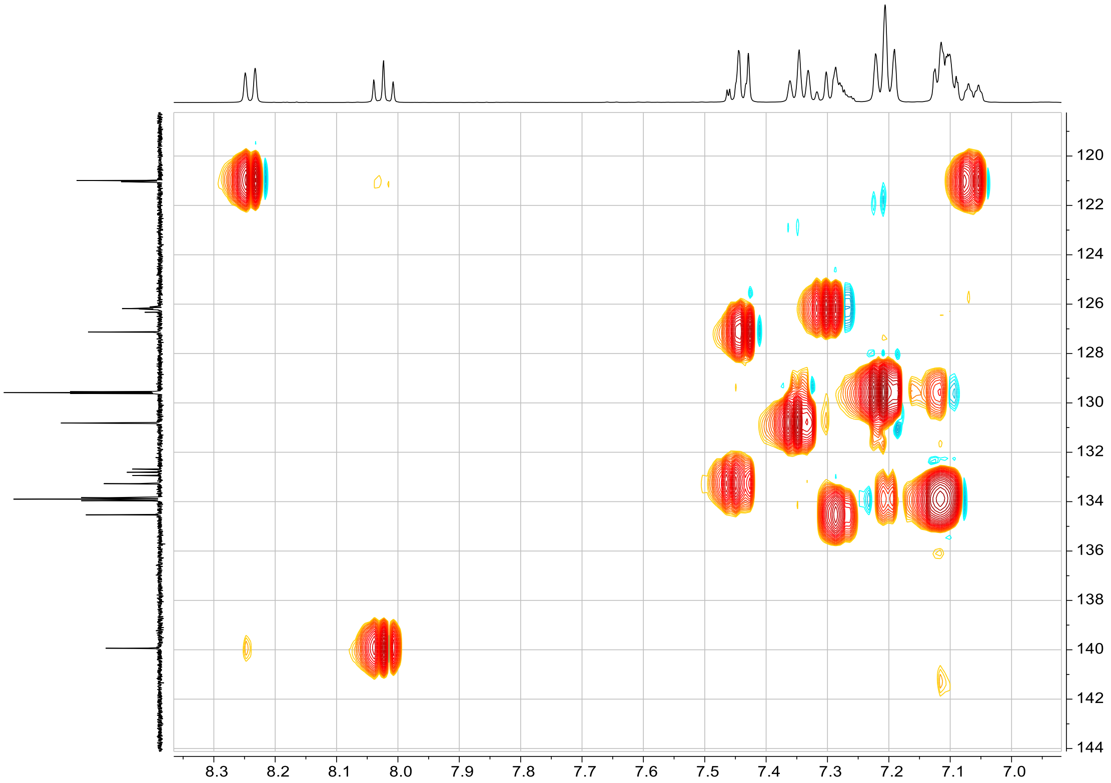

Fig. S23 The aromatic region of the HMQC spectrum (500 MHz ^{1}H, 126 MHz ^{13}C{^{1}H}, acetone-d_6, 298 K) of [Cu(POP)(Me_2bpy)][BF_4]. Scale: δ / ppm.

This journal is © The Royal Society of Chemistry 20xx

Please do not adjust margins

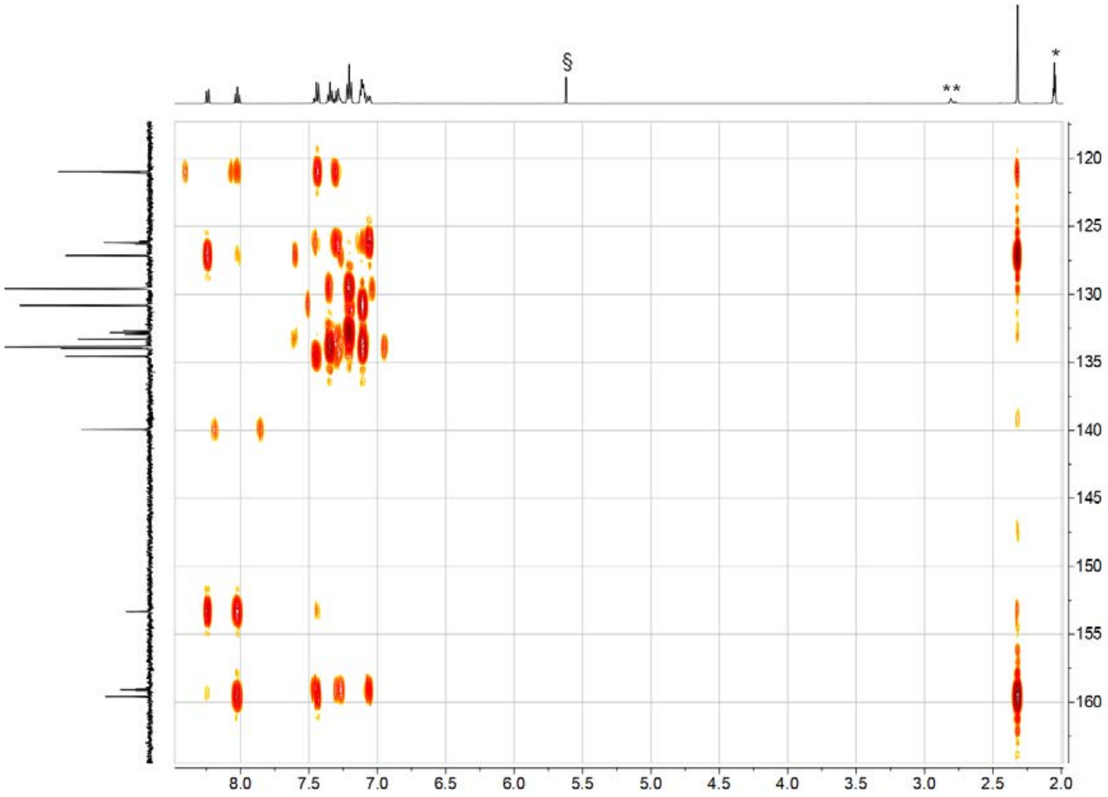

Fig. S24 Part of the HMBC spectrum (500 MHz 1H, 126 MHz $^{13}C\{^{1}H\}$, acetone-d_6, 298 K) of [Cu(POP)(Me$_2$bpy)][BF$_4$]. Scale: δ / ppm. ** = H_2O and HDO; § = CH_2Cl_2.

Please do not adjust margins

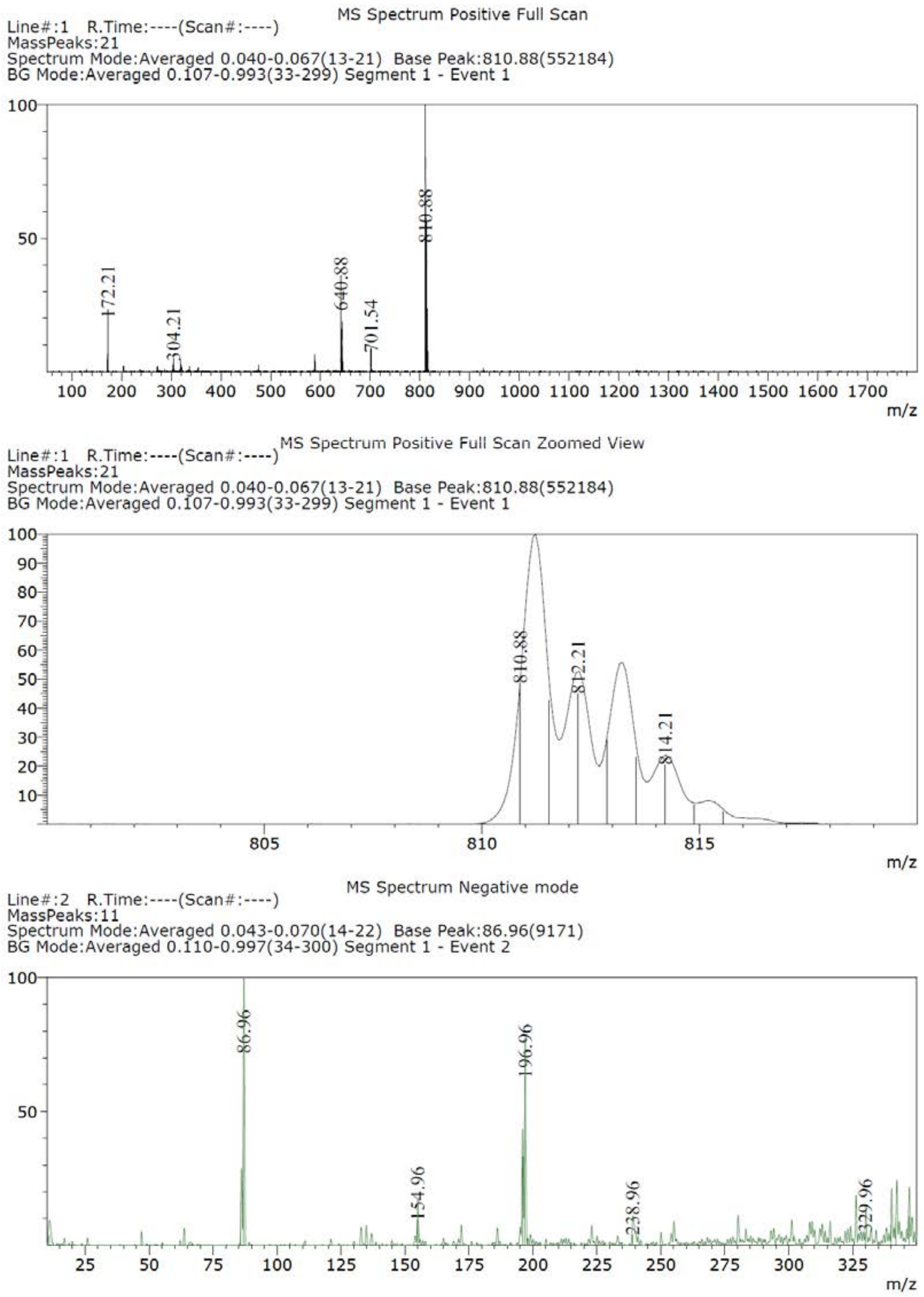

Fig. S25 The ESI mass spectrum (positive and negative mode) of [Cu(xantphos)(Mebpy)][BF_4].

This journal is © The Royal Society of Chemistry 20xx

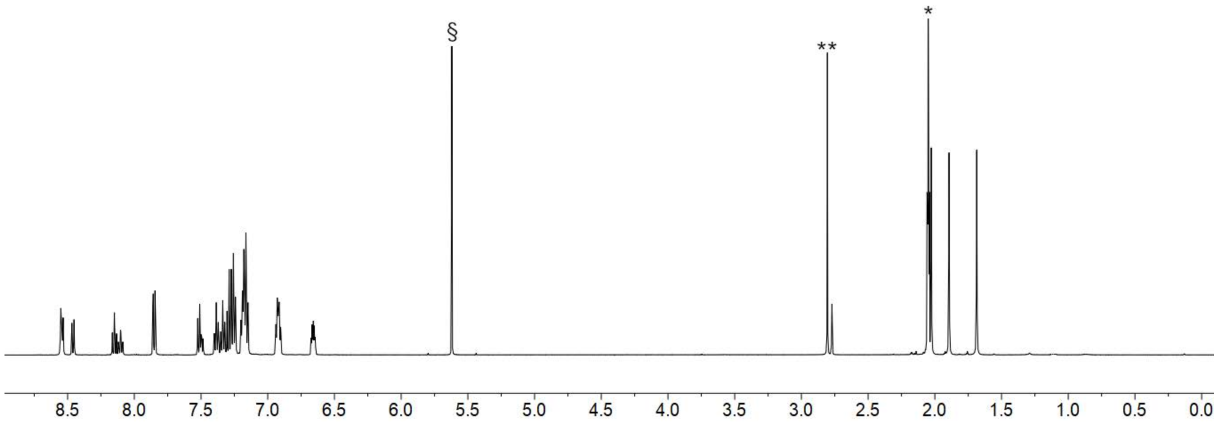

Fig. S26 ^1H NMR spectrum of [Cu(xantphos)(Mebpy)][BF_4] (500 MHz, 298 K, acetone-d6). * = residual acetone-d_5; ** = H_2O and HDO; § = CH_2Cl_2.

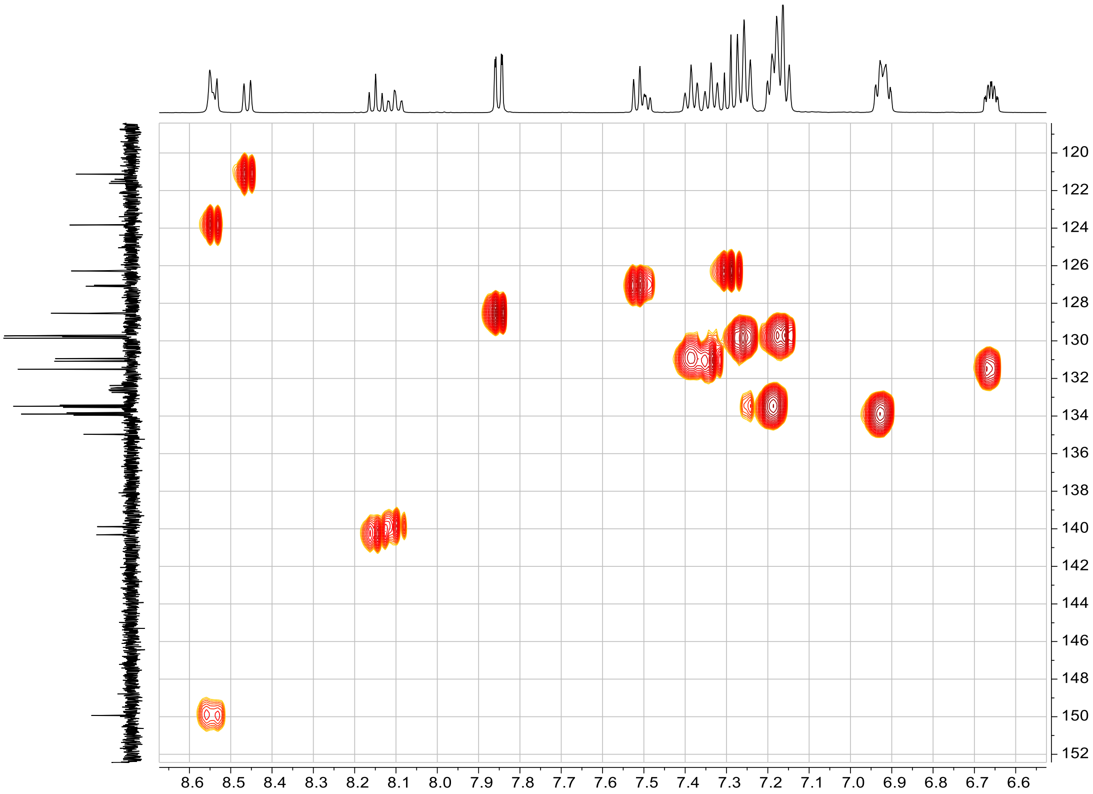

Fig. S27 The aromatic region of the HMQC spectrum (500 MHz ^1H, 126 MHz $^{13}C\{^1H\}$, acetone-d_6, 298 K) of [Cu(xantphos)(Mebpy)][BF_4]. Scale: δ / ppm.

Please do not adjust margins

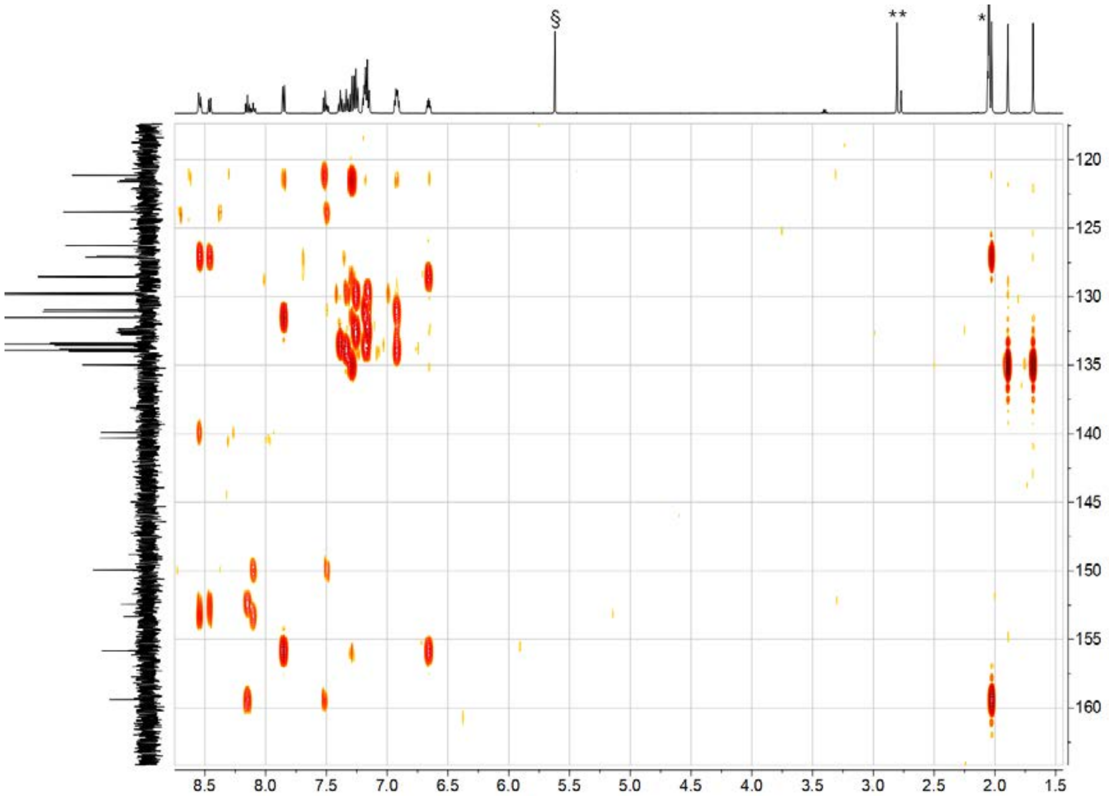

Fig. S28 Part of the HMBC spectrum (500 MHz 1H, 126 MHz $^{13}C\{^{1}H\}$, acetone-d_6, 298 K) of [Cu(xantphos)(Mebpy)][BF_4]. Scale: δ / ppm. ** = H_2O and HDO; § = CH_2Cl_2.

This journal is © The Royal Society of Chemistry 20xx

Please do not adjust margins

Please do not adjust margins

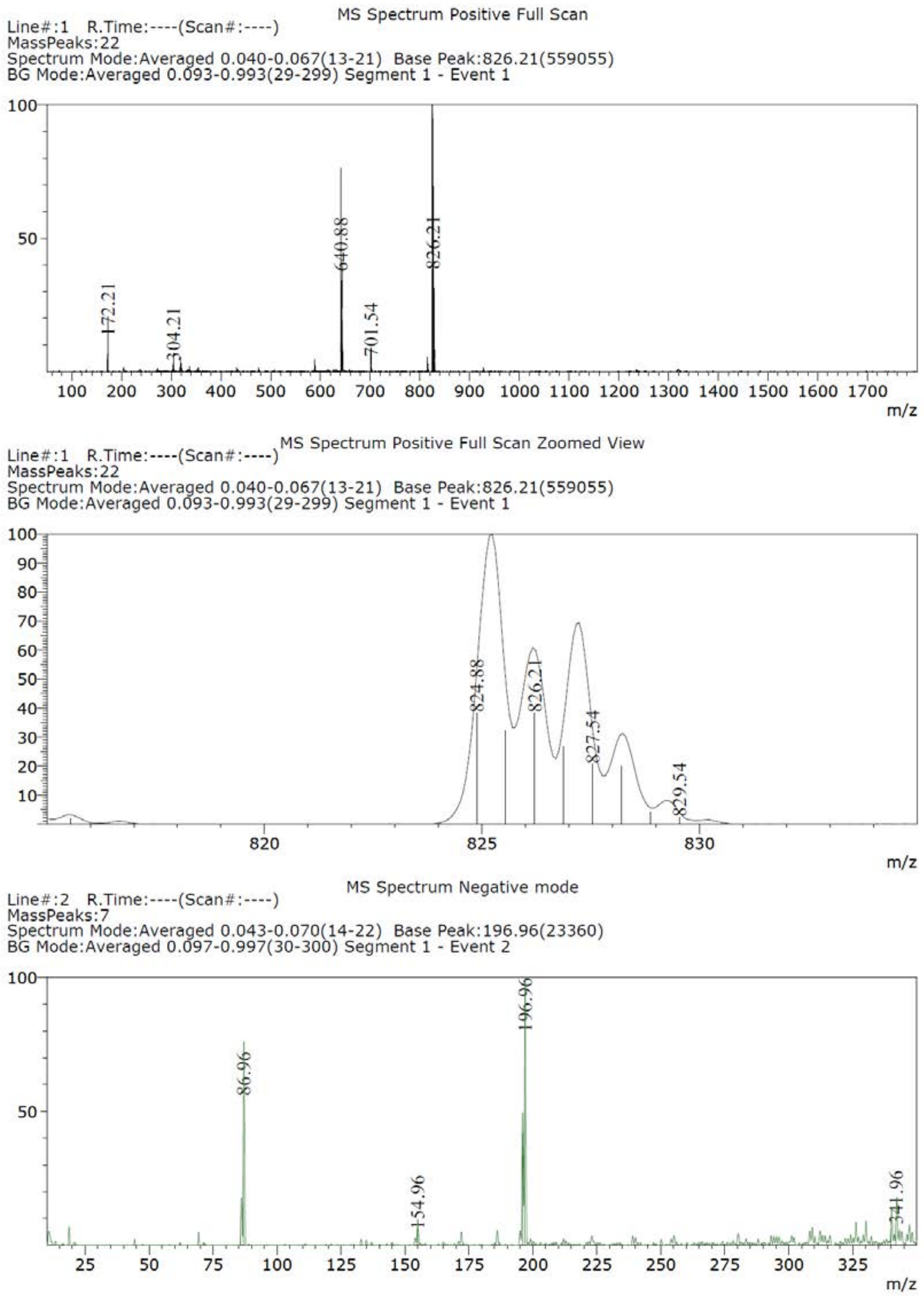

Fig. S29 The ESI mass spectrum (positive and negative mode) of [Cu(xantphos)(Me2bpy)][BF_4].

Please do not adjust margins

Please do not adjust margins

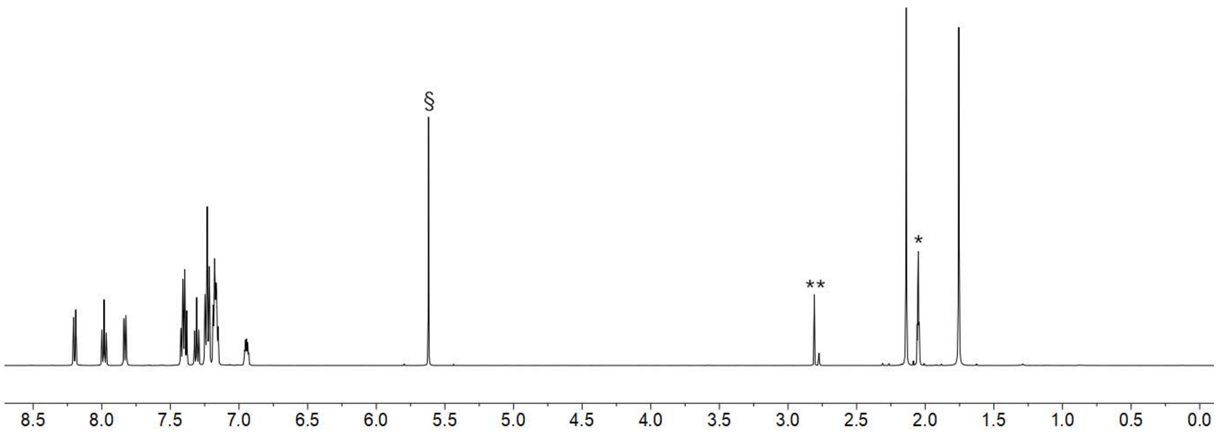

Fig. S30 ^1H NMR spectrum of [Cu(xantphos)(Me_2bpy)][BF_4] (500 MHz, 298 K, acetone-d6). * = residual acetone-d_5; ** = H_2O and HDO; § = CH_2Cl_2.

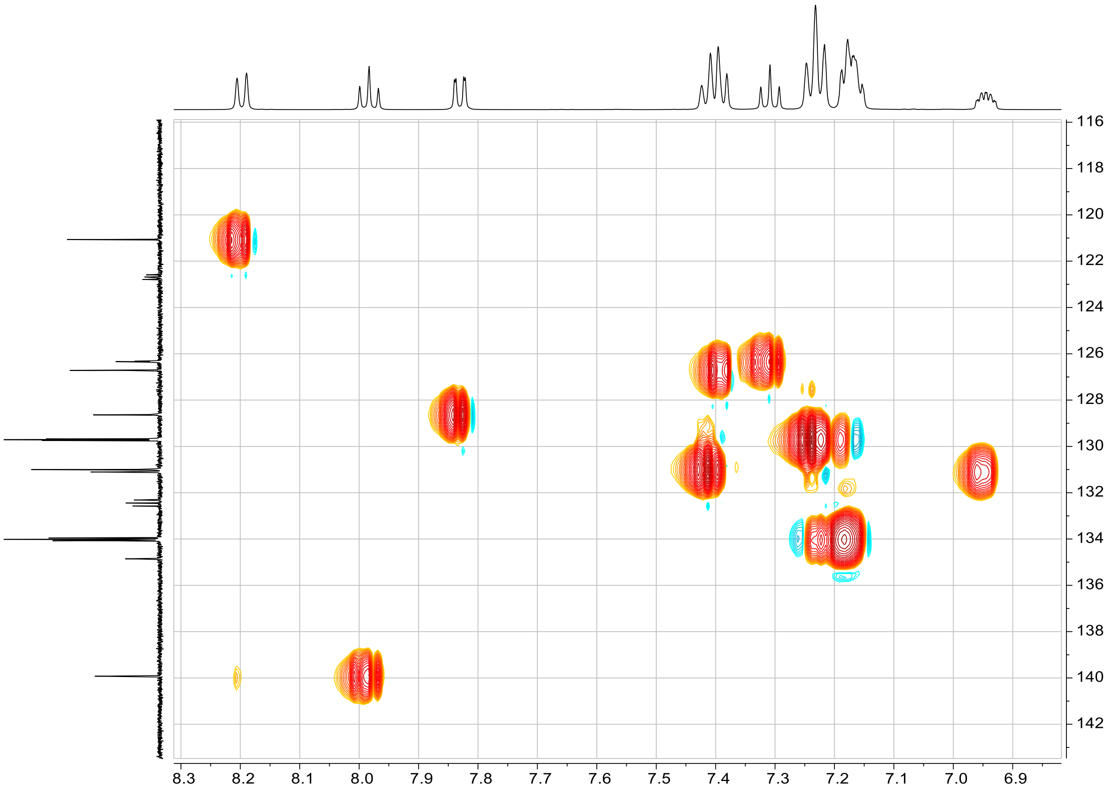

Fig. S31 The aromatic region of the HMQC spectrum (500 MHz ^1H, 126 MHz $^{13}C\{^1H\}$, acetone-d_6, 298 K) of [Cu(xantphos)(Me_2bpy)][BF_4]. Scale: δ / ppm.

This journal is © The Royal Society of Chemistry 20xx

Please do not adjust margins

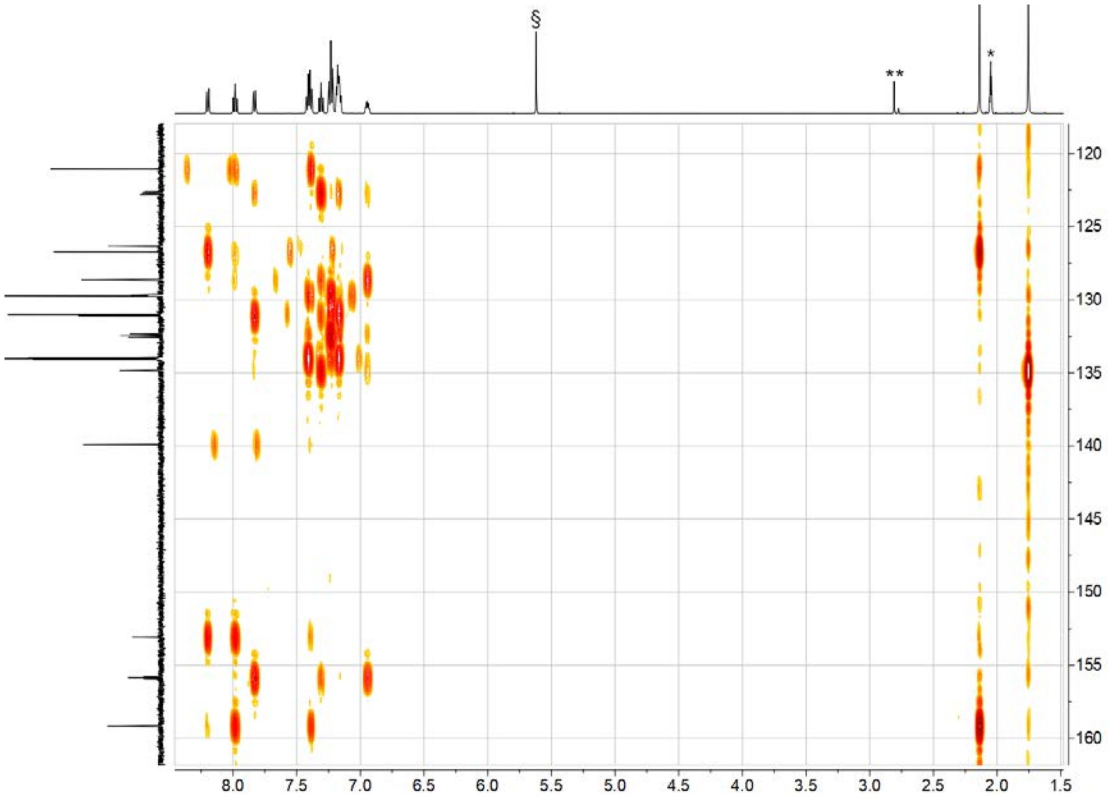

Fig. S32 Part of the HMBC spectrum (500 MHz 1H, 126 MHz $^{13}C\{^1H\}$, acetone-d_6, 298 K) of [Cu(xantphos)(Me_2bpy)][BF_4]. Scale: δ / ppm. ** = H_2O and HDO; § = CH_2Cl_2.

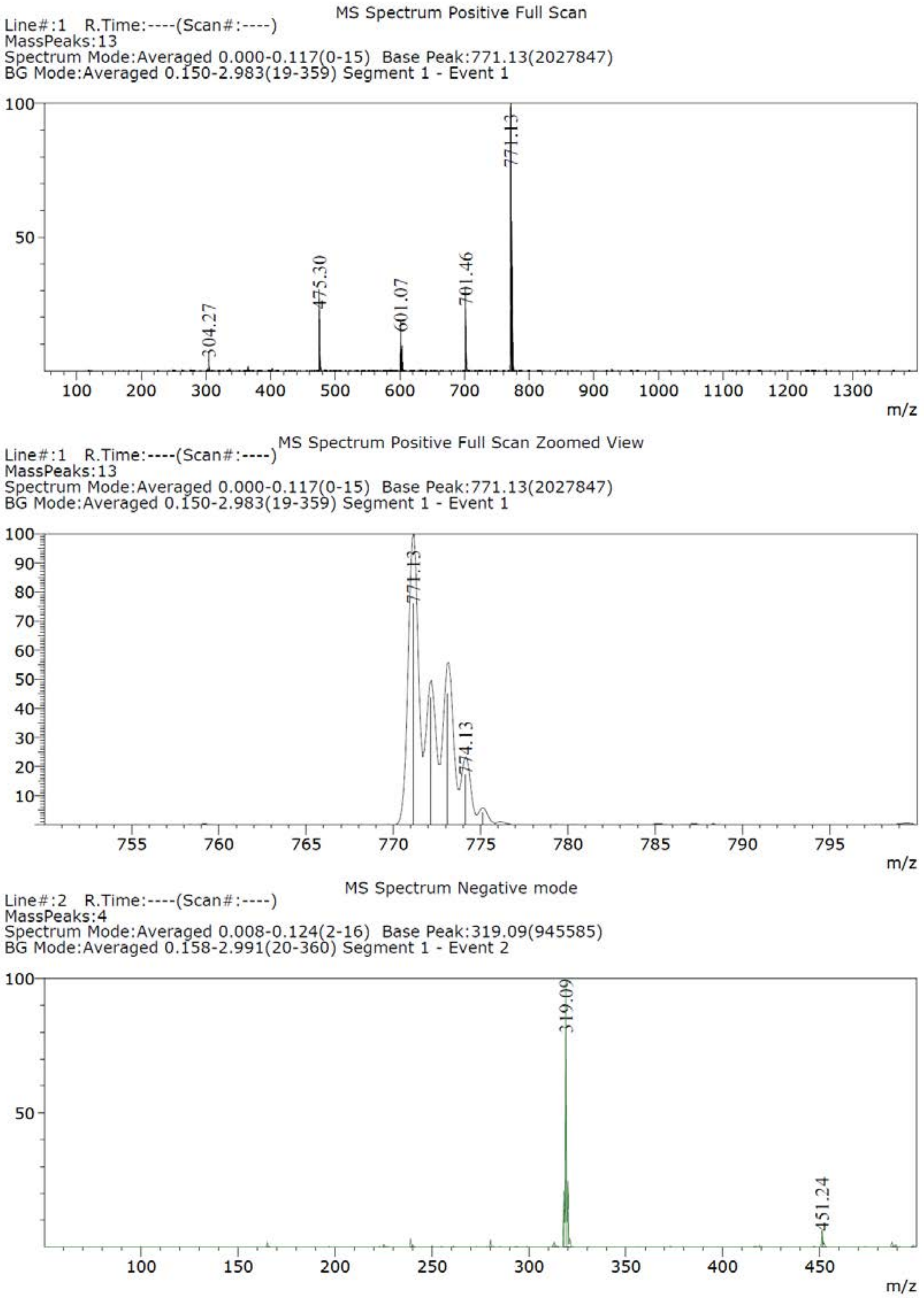

Fig. S33 The ESI mass spectrum (positive and negative mode) of [Cu(POP)(Mebpy)][BPh_4].

This journal is © The Royal Society of Chemistry 20xx

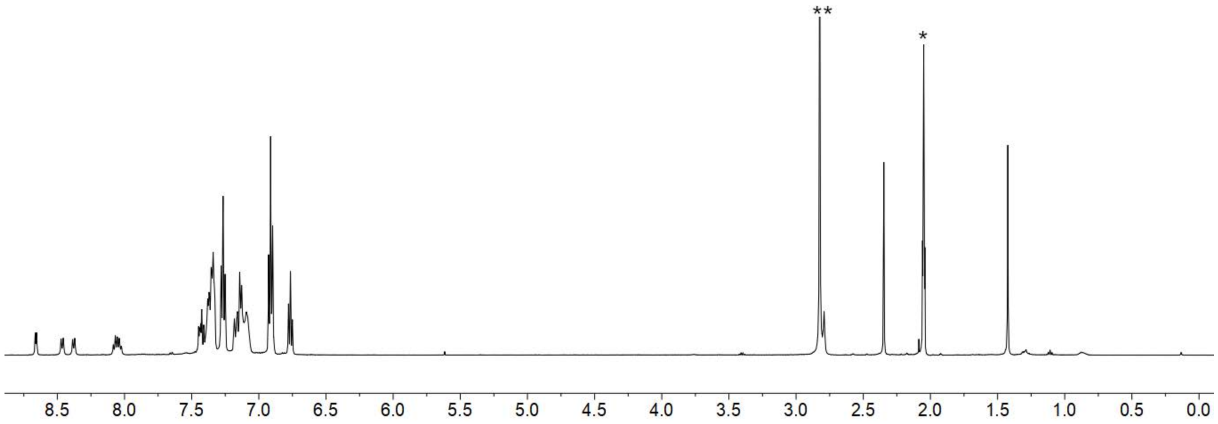

Fig. S34 ^{1}H NMR spectrum of [Cu(POP)(Mebpy)][BPh_4] (500 MHz, 298 K, acetone-d6). * = residual acetone-d_5; ** = H_2O and HDO.

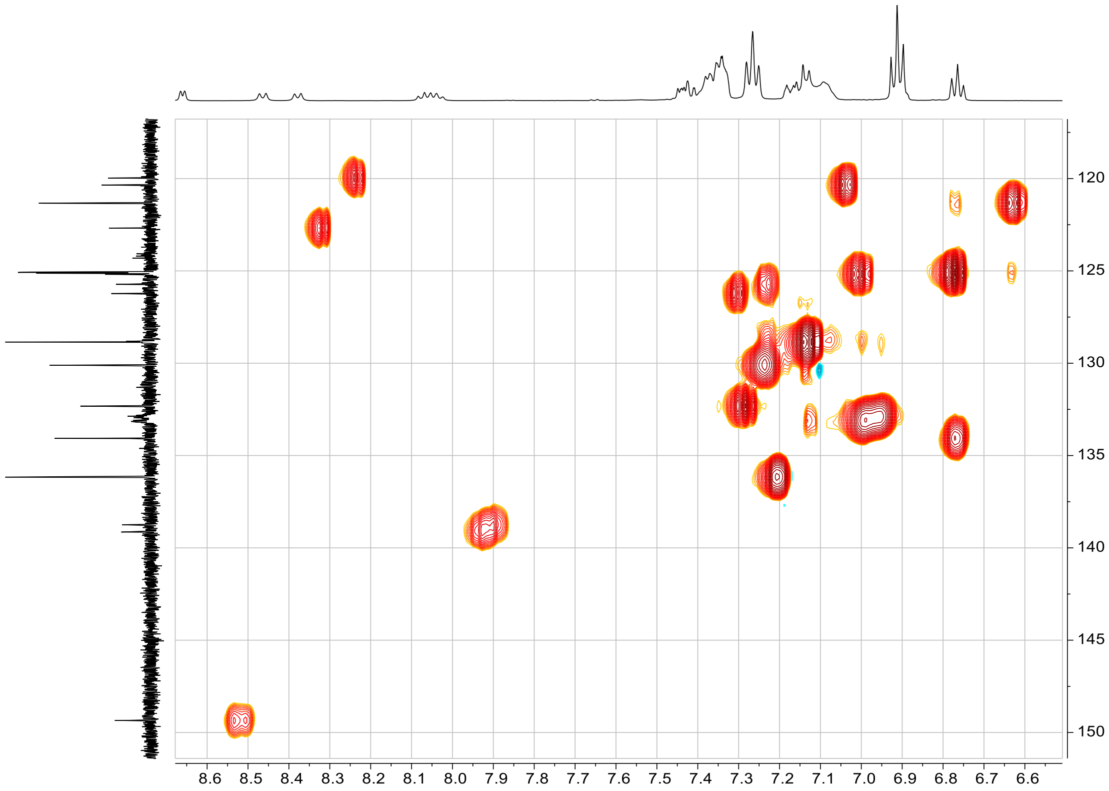

Fig. S35 The aromatic region of the HMQC spectrum (500 MHz ^{1}H, 126 MHz $^{13}C\{^{1}H\}$, acetone-d_6, 298 K) of [Cu(POP)(Mebpy)][BPh_4]. Scale: δ / ppm.

Please do not adjust margins

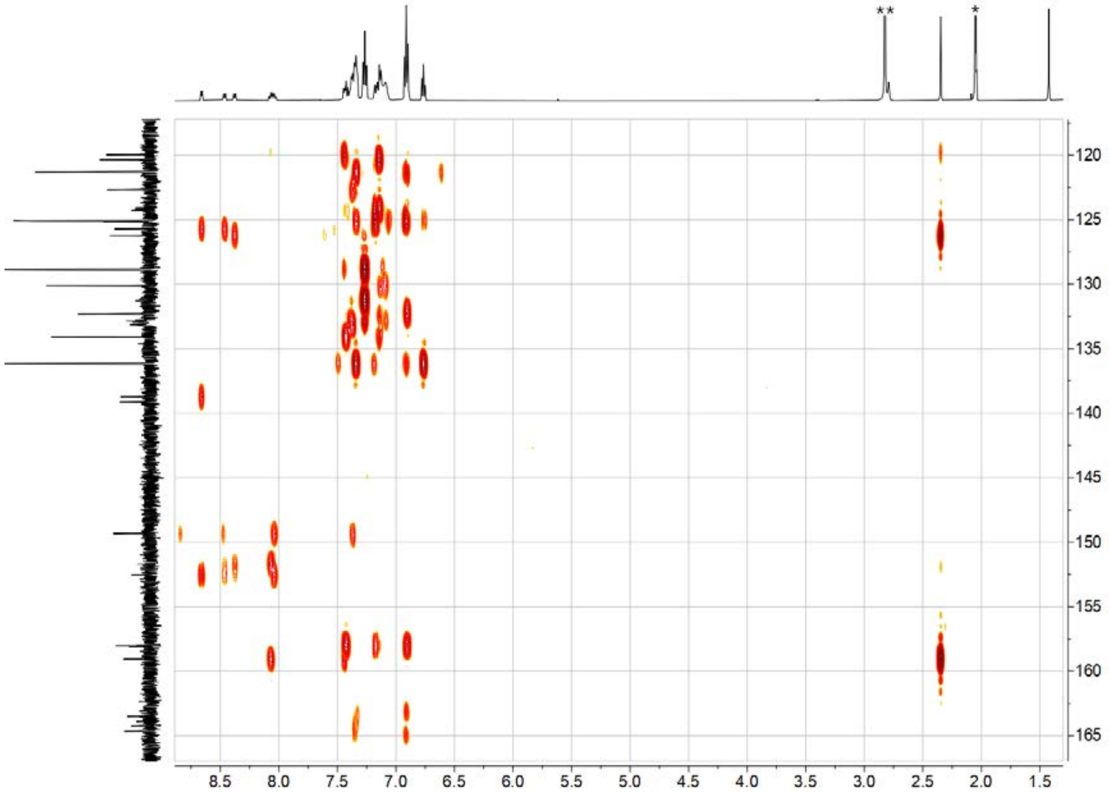

Fig. S36 Part of the HMBC spectrum (500 MHz 1H, 126 MHz $^{13}C\{^1H\}$, acetone-d_6, 298 K) of [Cu(POP)(Mebpy)][BPh_4]. Scale: δ / ppm. ** = H_2O and HDO.

 This journal is © The Royal Society of Chemistry 20xx

Please do not adjust margins

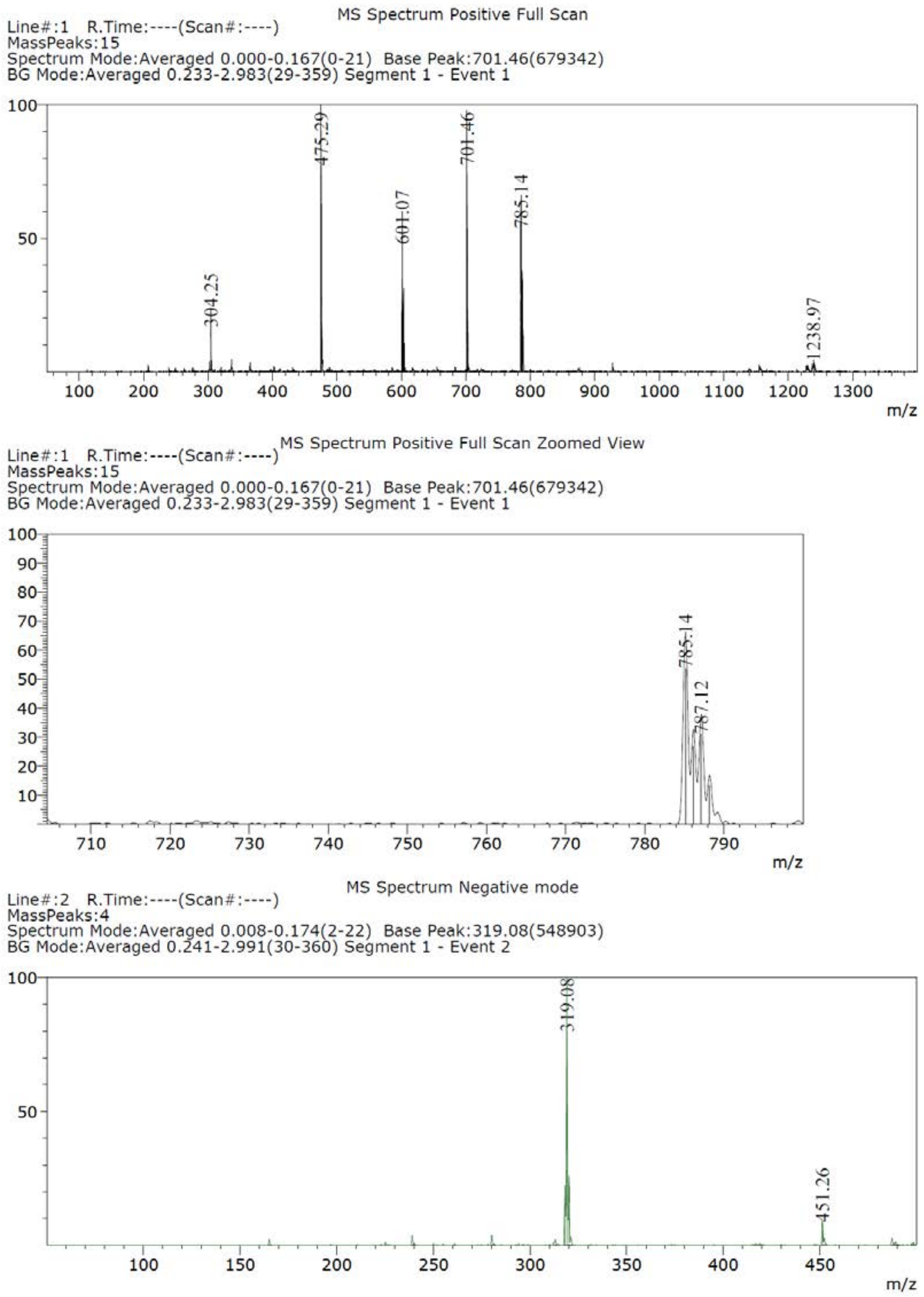

Fig. S37 The ESI mass spectrum (positive and negative mode) of [Cu(POP)(Me_2bpy)][BPh_4].

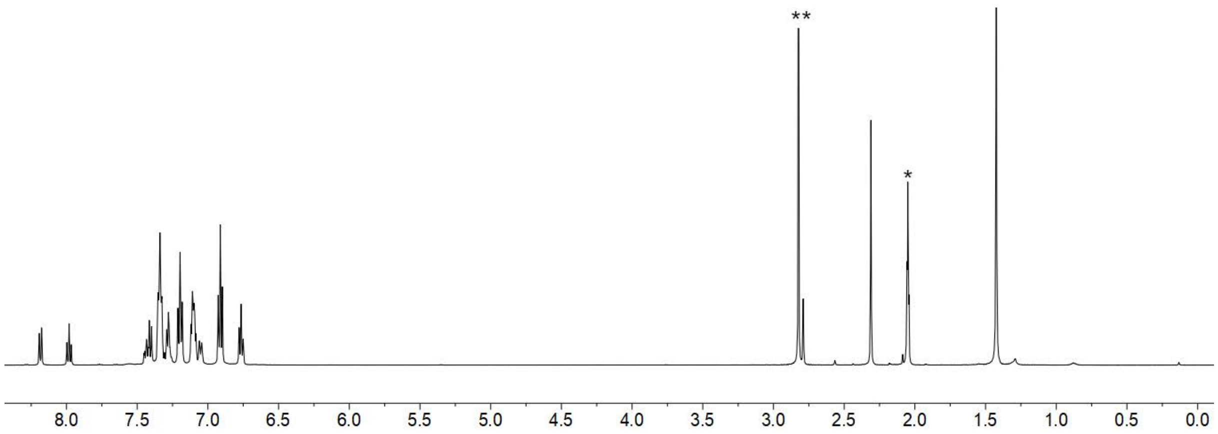

Fig. S38 ^{1}H NMR spectrum of [Cu(POP)(Me_2bpy)][BPh_4] (500 MHz, 298 K, acetone-d6). * = residual acetone-*d*$_5$; ** = H_2O and HDO; § = CH_2Cl_2.

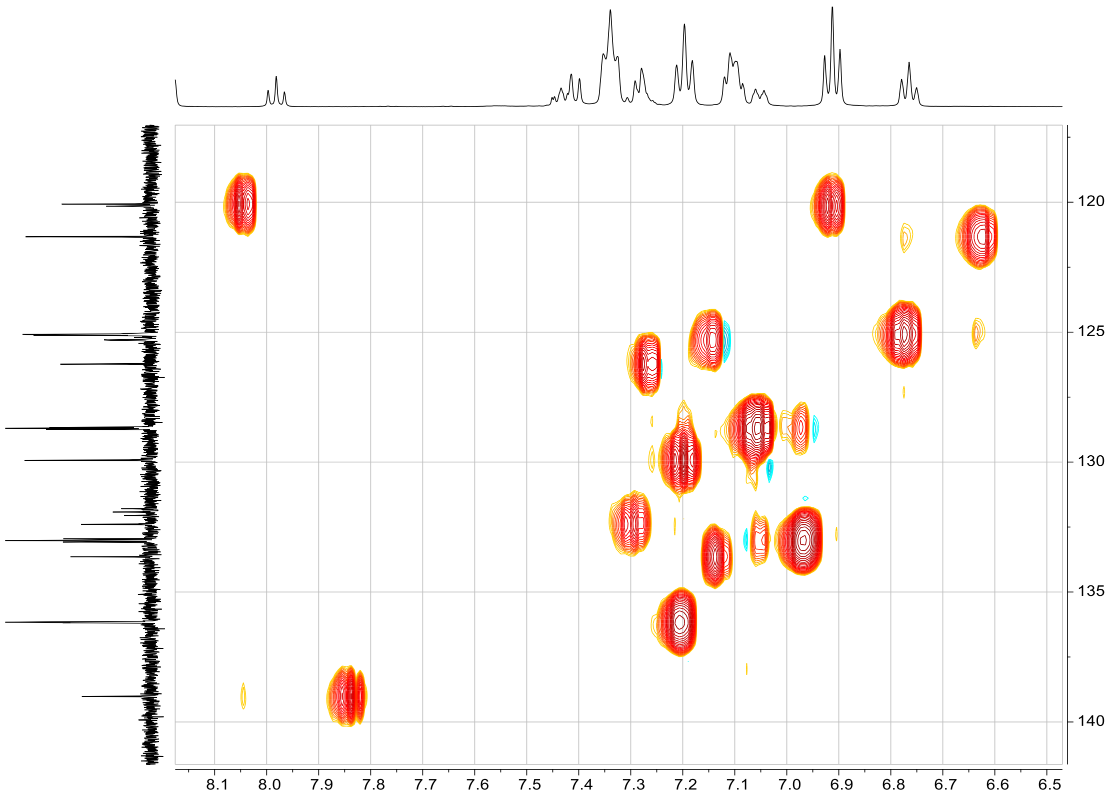

Fig. S39 The aromatic region of the HMQC spectrum (500 MHz ^{1}H, 126 MHz ^{13}C{^{1}H}, acetone-*d*$_6$, 298 K) of [Cu(POP)(Me_2bpy)][BPh_4]. Scale: δ / ppm.

This journal is © The Royal Society of Chemistry 20xx

Please do not adjust margins

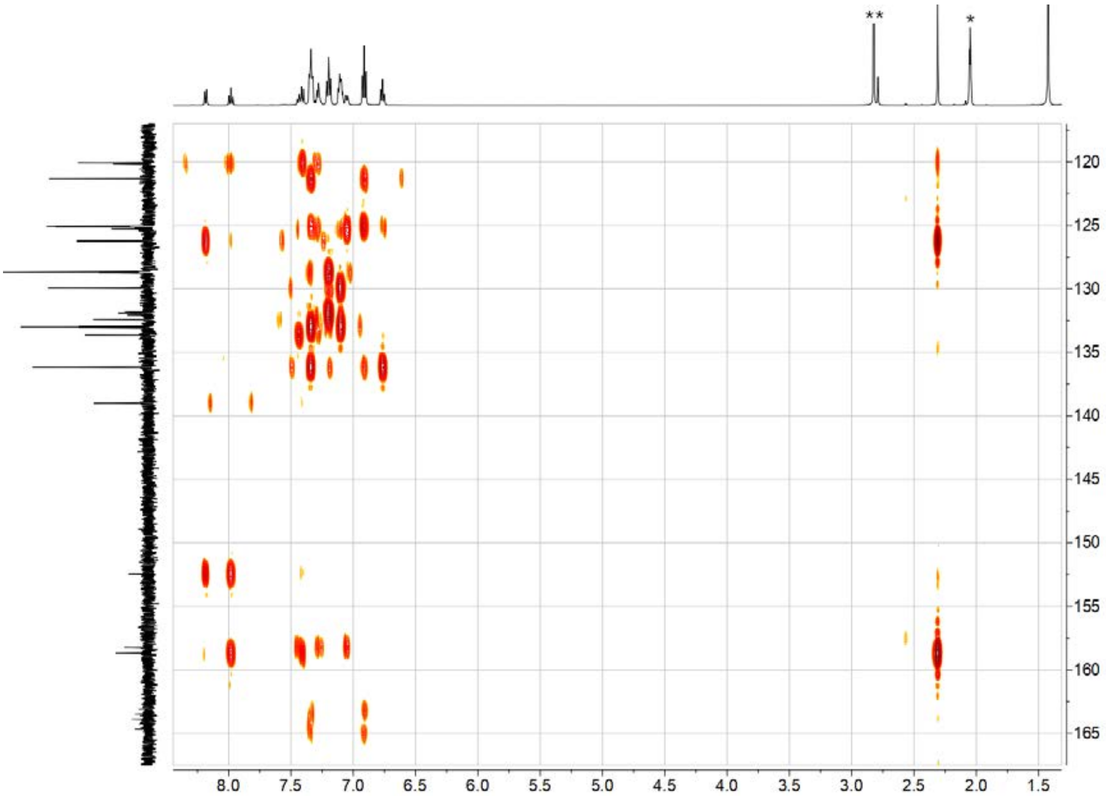

Fig. S40 Part of the HMBC spectrum (500 MHz 1H, 126 MHz $^{13}C\{^{1}H\}$, acetone-d_6, 298 K) of [Cu(POP)(Me_2bpy)][BPh_4]. Scale: δ / ppm. ** = H_2O and HDO.

Please do not adjust margins

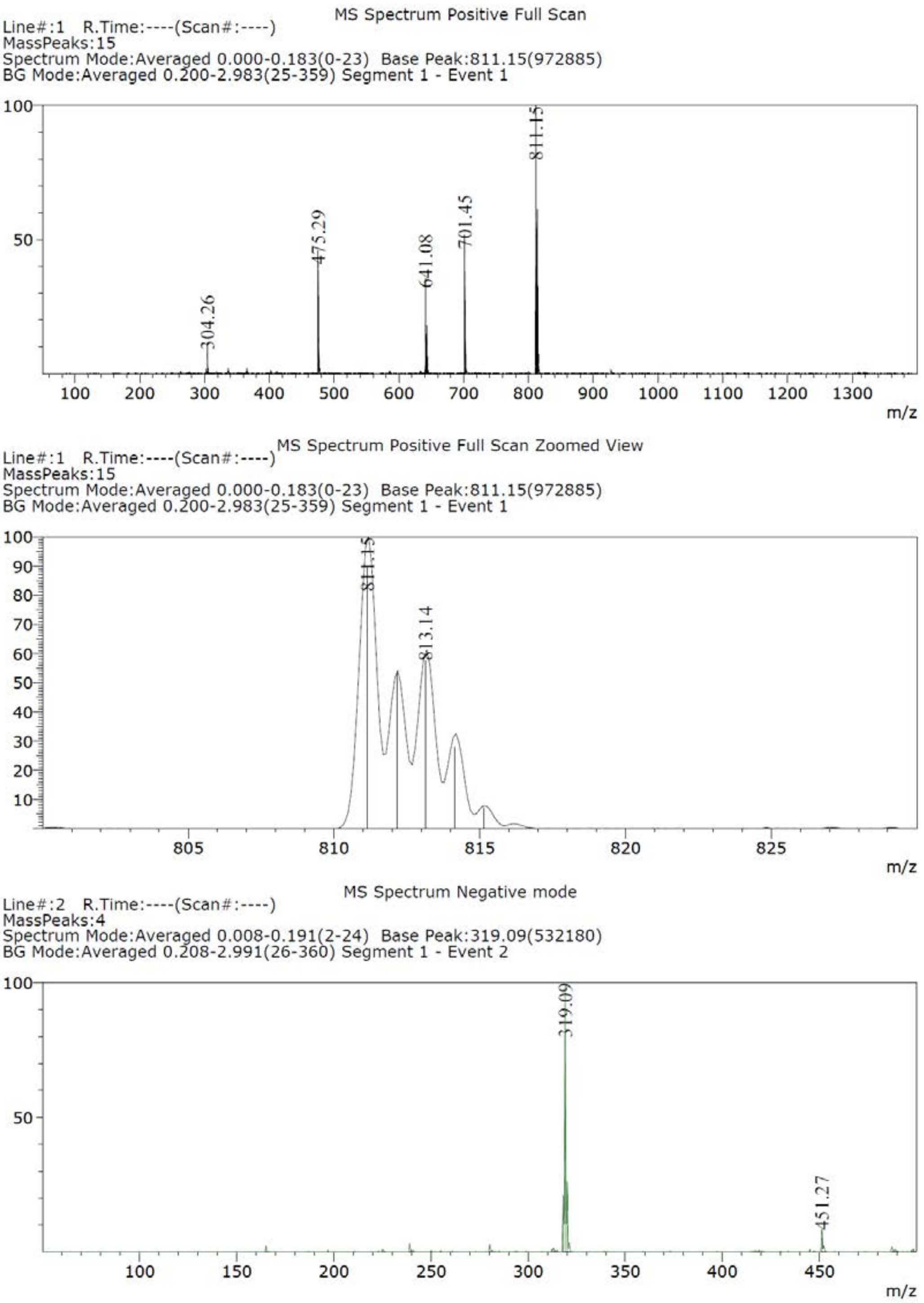

Fig. S41 The ESI mass spectrum (positive and negative mode) of [Cu(xantphos)(Mebpy)][BPh_4].

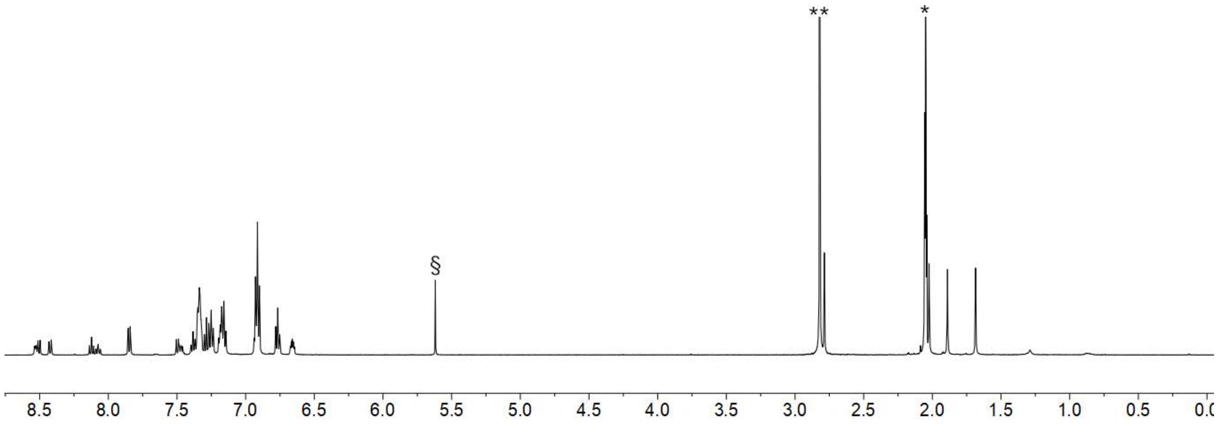

Fig. S42 ^{1}H NMR spectrum of [Cu(xantphos)(Mebpy)][BPh_4] (500 MHz, 298 K, acetone-d6). * = residual acetone-d_5; ** = H_2O and HDO; § = CH_2Cl_2.

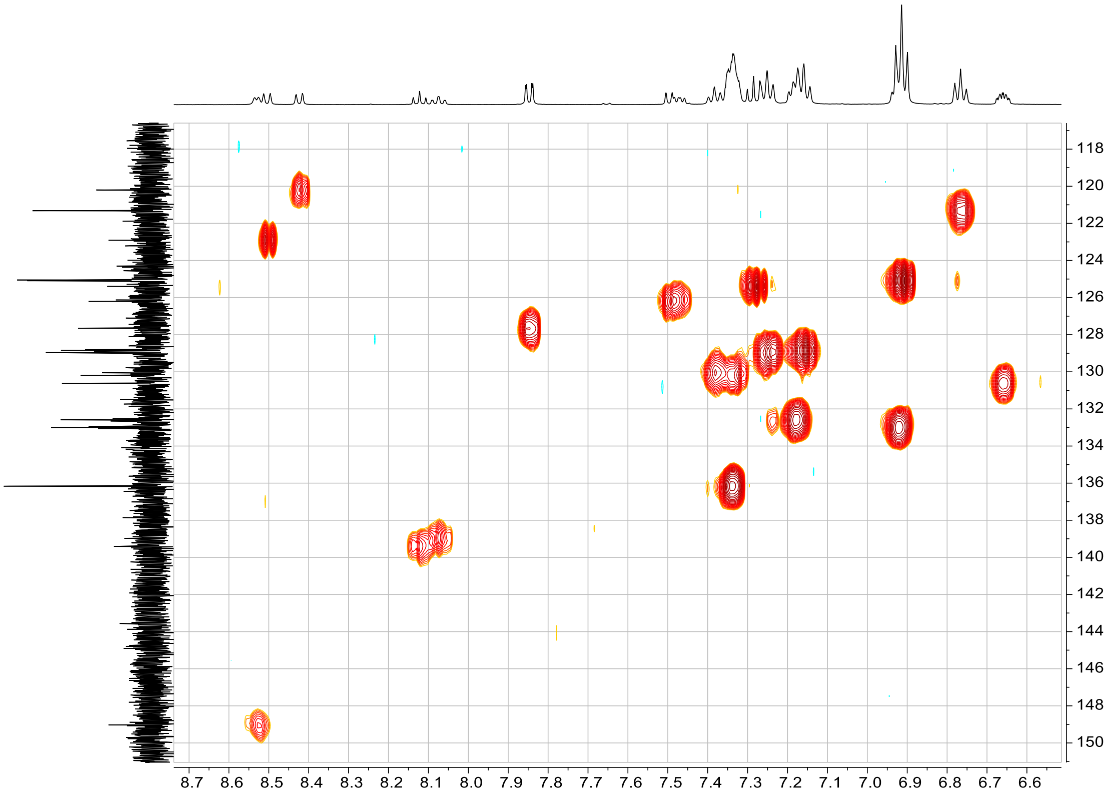

Fig. S43 The aromatic region of the HMQC spectrum (500 MHz ^{1}H, 126 MHz $^{13}C\{^{1}H\}$, acetone-d_6, 298 K) of [Cu(xantphos)(Mebpy)][BPh_4]. Scale: δ / ppm.

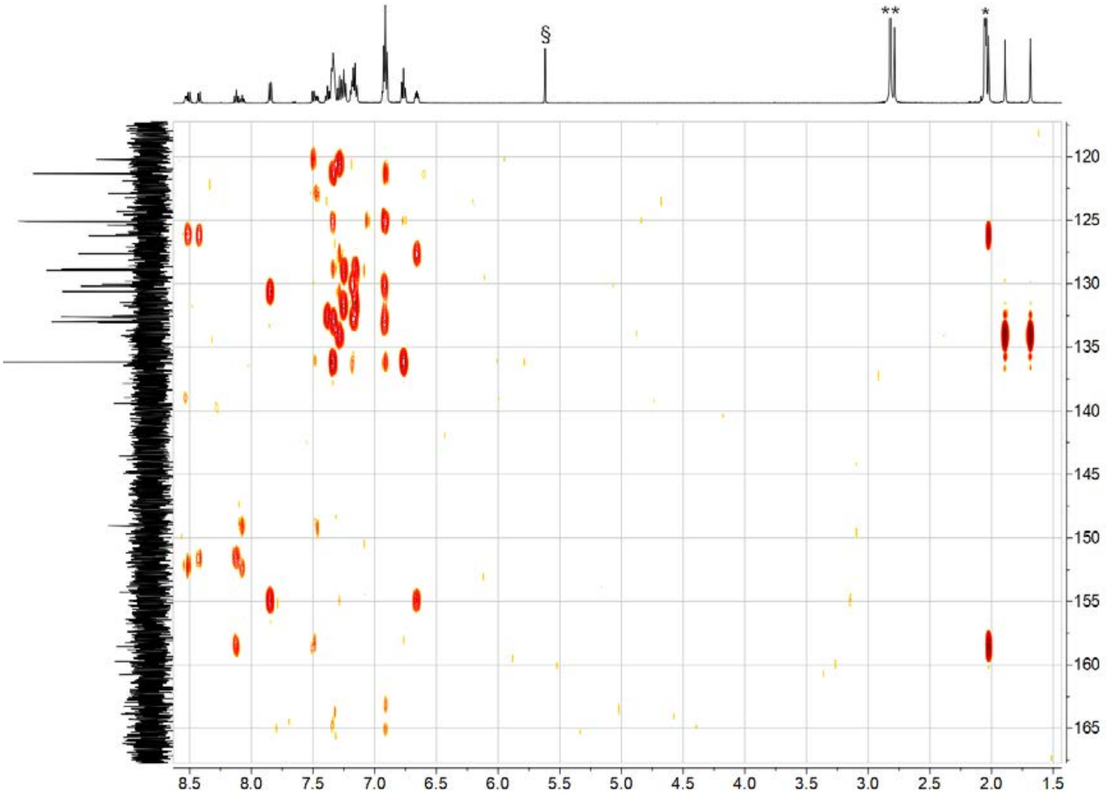

Fig. S44 Part of the HMBC spectrum (500 MHz 1H, 126 MHz $^{13}C\{^1H\}$, acetone-d_6, 298 K) of [Cu(xantphos)(Mebpy)][BPh_4]. Scale: δ / ppm. ** = H_2O and HDO; § = CH_2Cl_2.

This journal is © The Royal Society of Chemistry 20xx

Please do not adjust margins

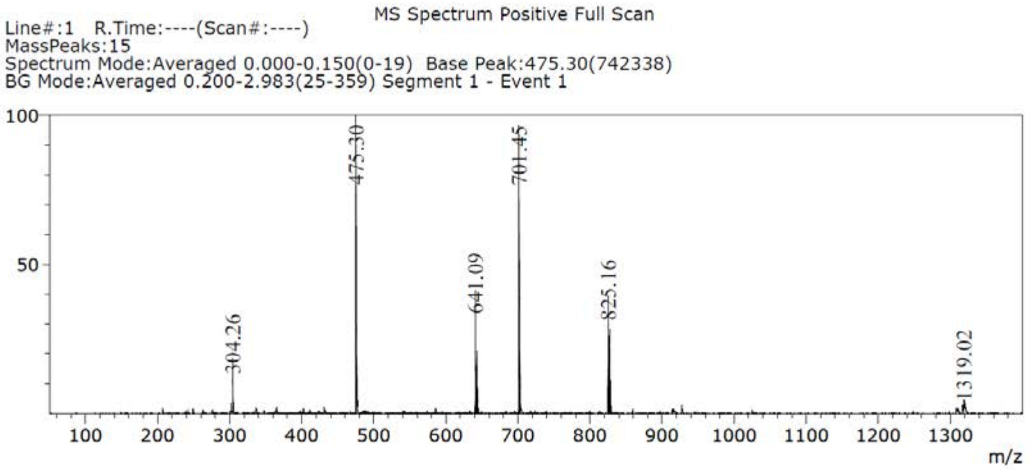

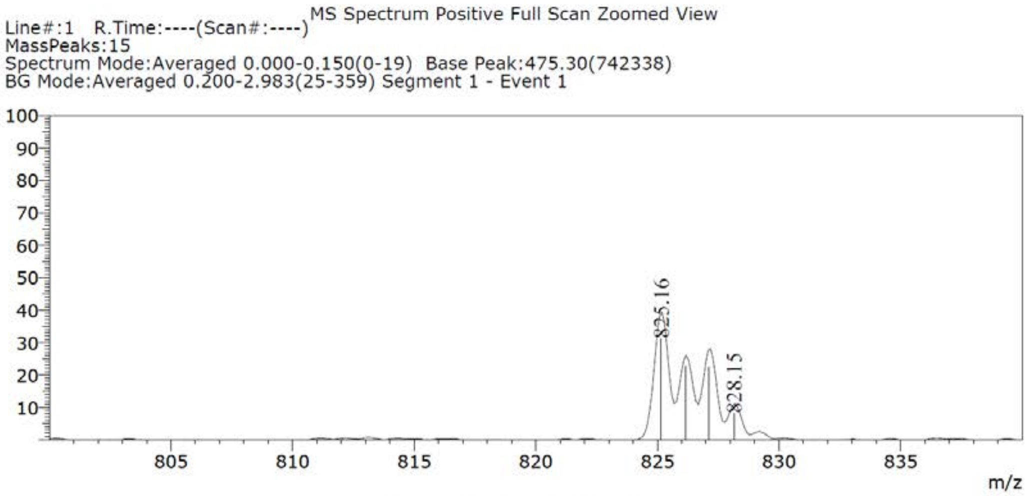

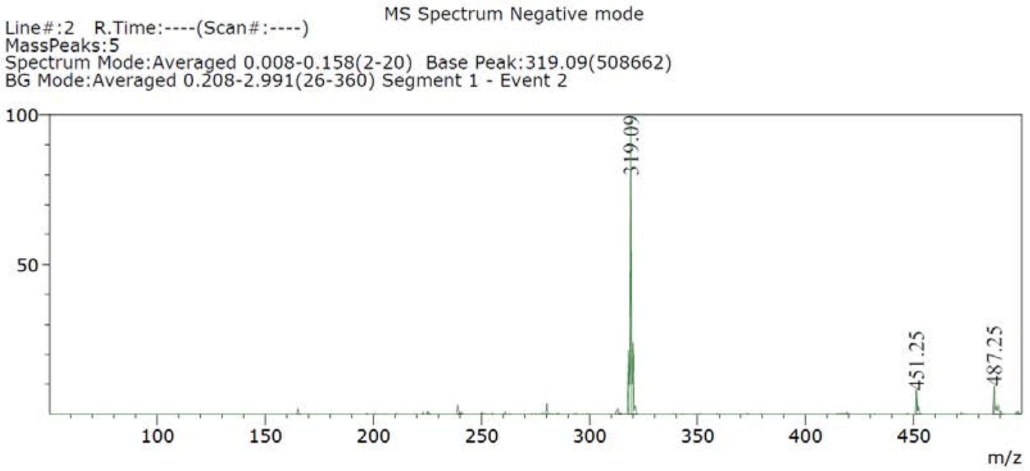

Fig. S45 The ESI mass spectrum (positive and negative mode) of [Cu(xantphos)(Me_2bpy)][BPh_4].

Please do not adjust margins

Please do not adjust margins

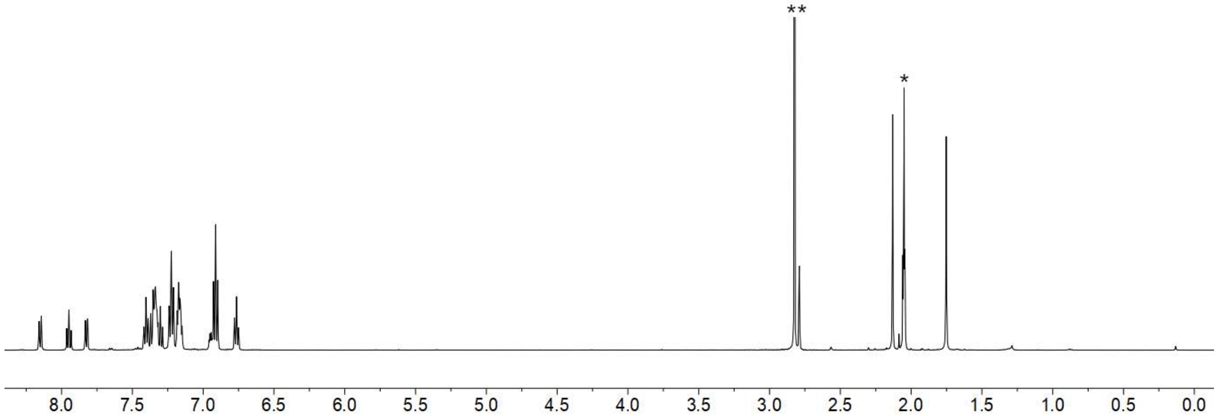

Fig. S46 ^{1}H NMR spectrum of [Cu(xantphos)(Me_2bpy)][BPh_4] (500 MHz, 298 K, acetone-d6). * = residual acetone-d_5; ** = H_2O and HDO; § = CH_2Cl_2.

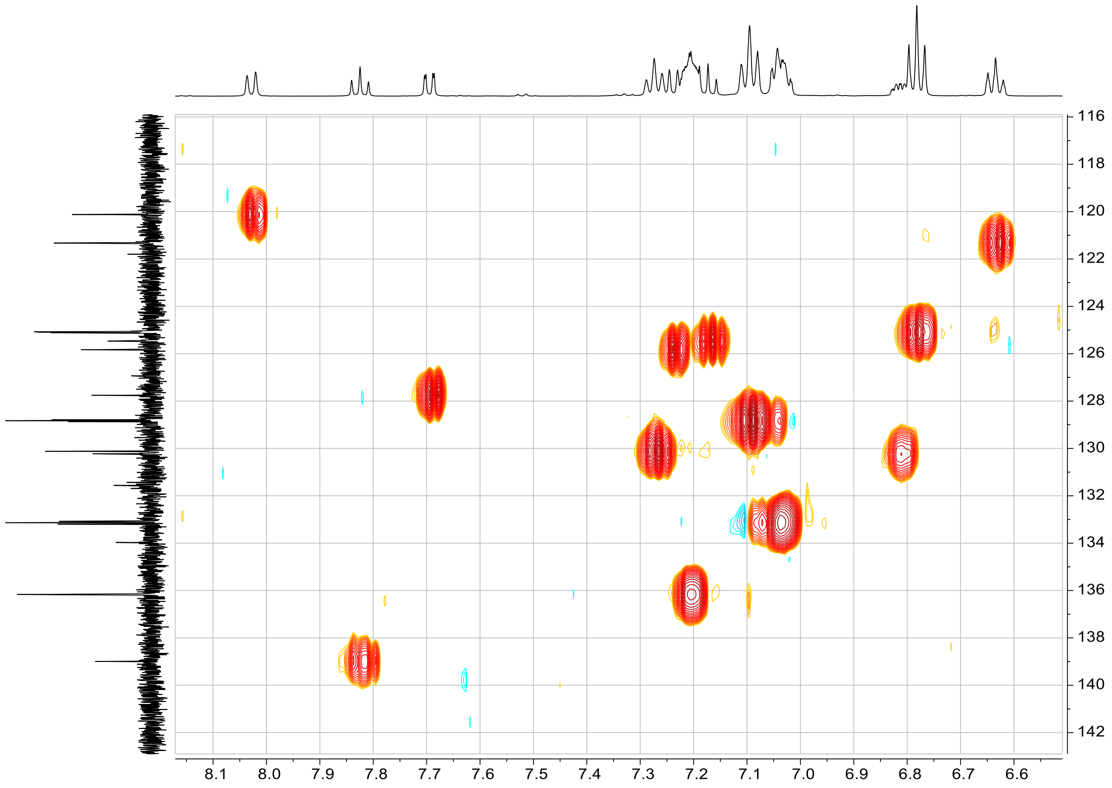

Fig. S47 The aromatic region of the HMQC spectrum (500 MHz ^{1}H, 126 MHz $^{13}C\{^{1}H\}$, acetone-d_6, 298 K) of [Cu(xantphos)(Me_2bpy)][BPh_4]. Scale: δ / ppm.

This journal is © The Royal Society of Chemistry 20xx

Please do not adjust margins

Please do not adjust margins

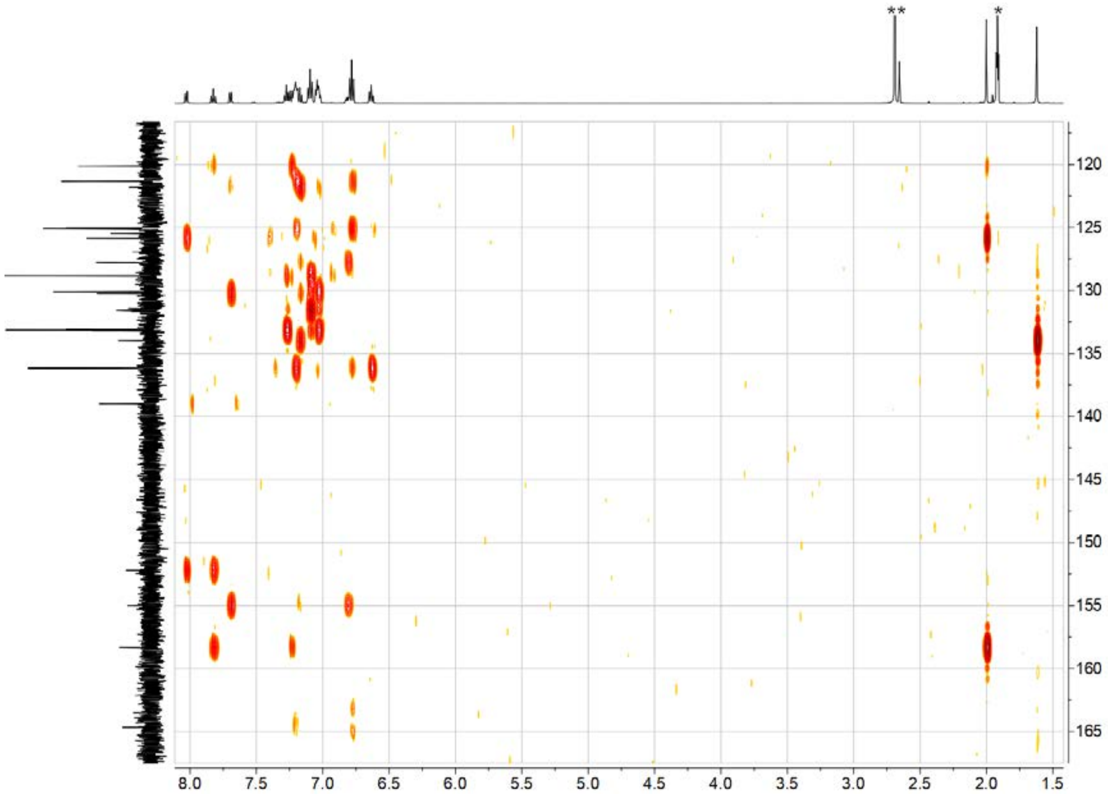

Fig. S48 Part of the HMBC spectrum (500 MHz 1H, 126 MHz $^{13}C\{^{1}H\}$, acetone-d_6, 298 K) of [Cu(xantphos)(Me_2bpy)][BPh_4]. Scale: δ / ppm. ** = H_2O and HDO.

Please do not adjust margins

Please do not adjust margins

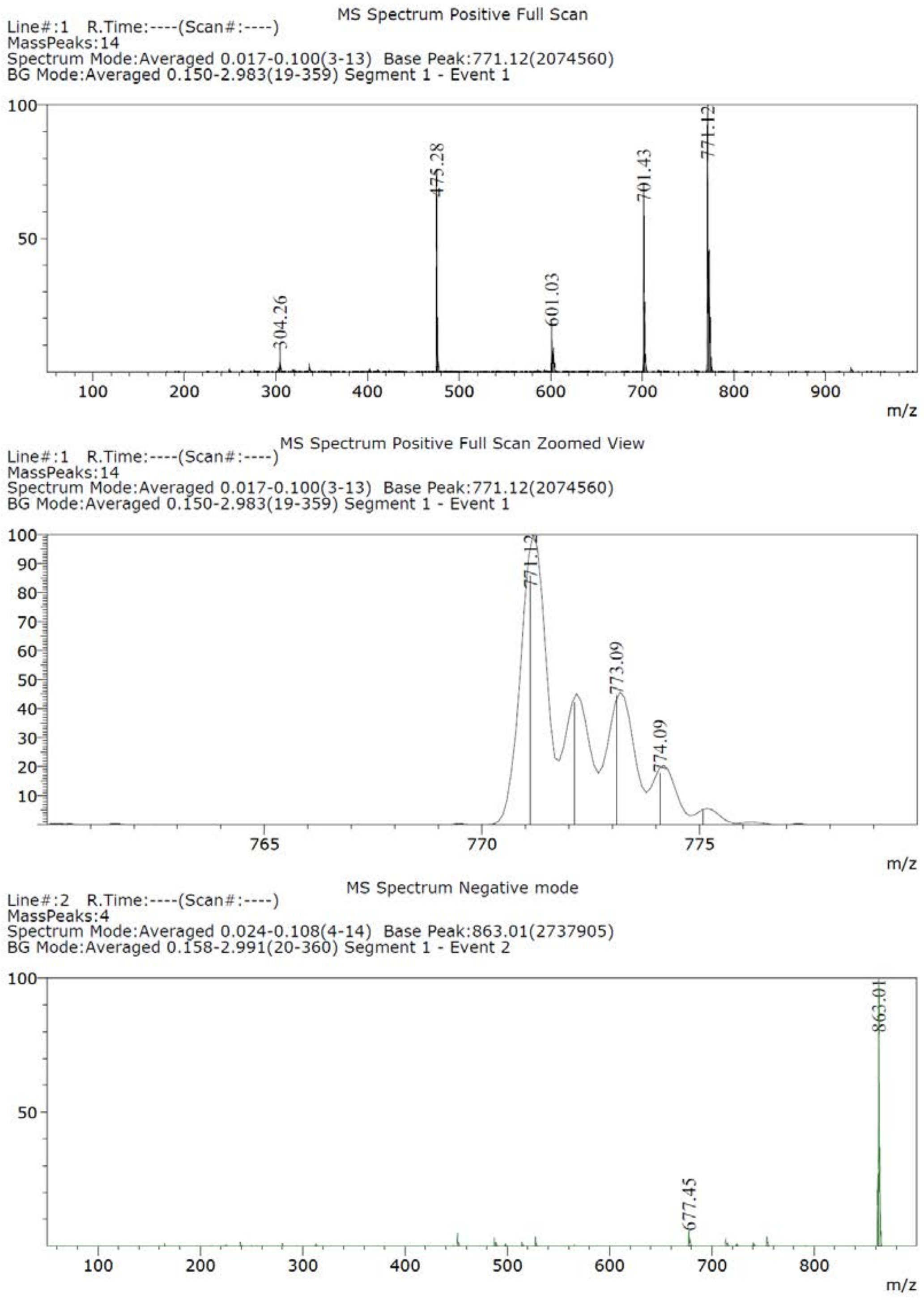

Fig. S49 The ESI mass spectrum (positive and negative mode) of [Cu(POP)(Mebpy)][BAr$^F{}_4$].

 This journal is © The Royal Society of Chemistry 20xx

Please do not adjust margins

Please do not adjust margins

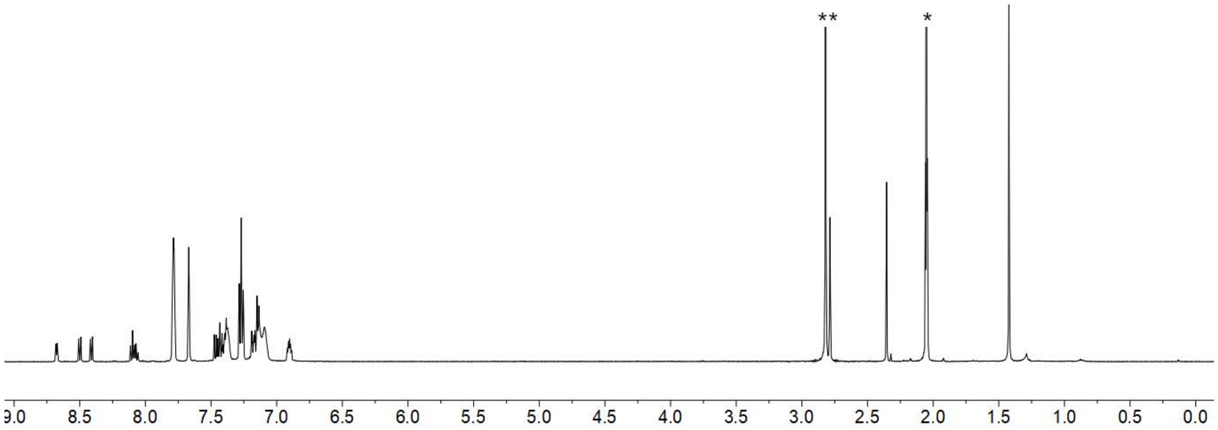

Fig. S50 ^{1}H NMR spectrum of [Cu(POP)(Mebpy)][BAr$^{F}_{4}$] (500 MHz, 298 K, acetone-d6). * = residual acetone-d_5; ** = H_2O and HDO.

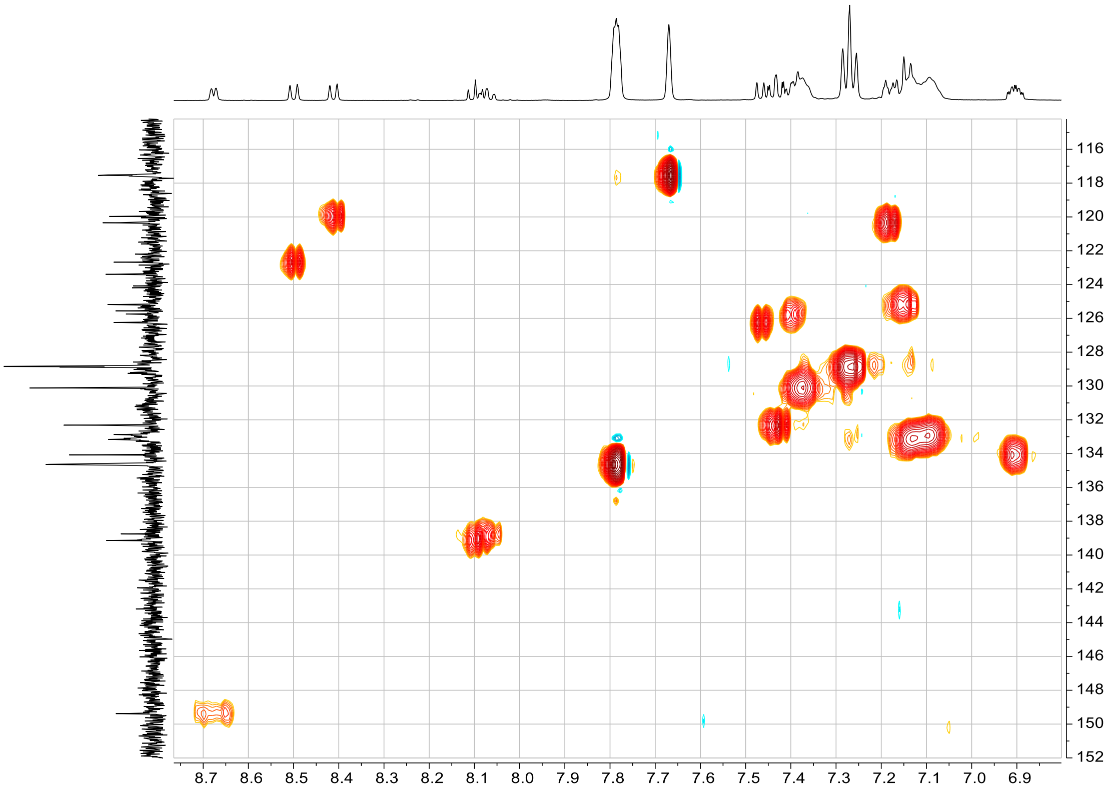

Fig. S51 The aromatic region of the HMQC spectrum (500 MHz ^{1}H, 126 MHz $^{13}C\{^{1}H\}$, acetone-d_6, 298 K) of [Cu(POP)(Mebpy)][BAr$^{F}_{4}$]. Scale: δ / ppm.

Please do not adjust margins

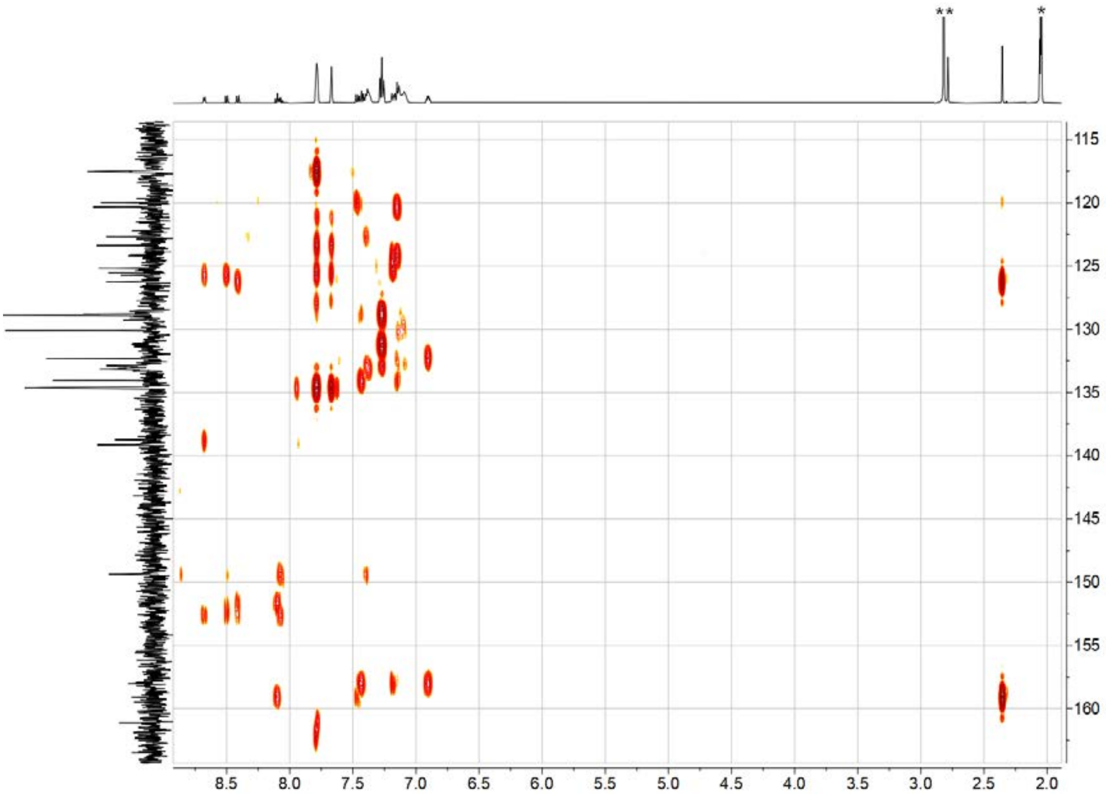

Fig. S52 Part of the HMBC spectrum (500 MHz 1H, 126 MHz $^{13}C\{^{1}H\}$, acetone-d_6, 298 K) of [Cu(POP)(Mebpy)][BAr$^{F}_{4}$]. Scale: δ / ppm. ** = H_2O and HDO.

 This journal is © The Royal Society of Chemistry 20xx

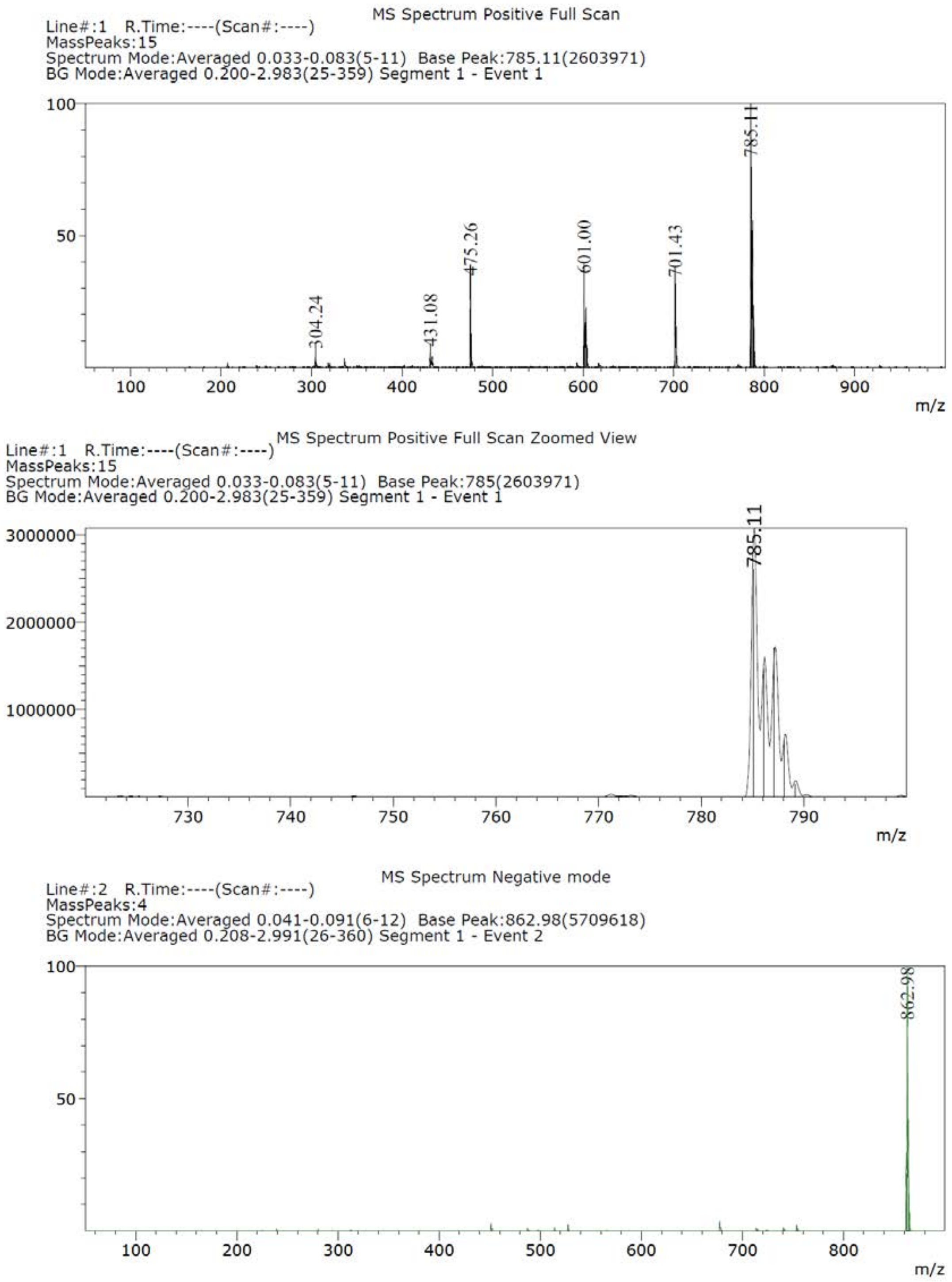

Fig. S53 The ESI mass spectrum (positive and negative mode) of [Cu(POP)(Me_2bpy)][$BAr^F{}_4$].

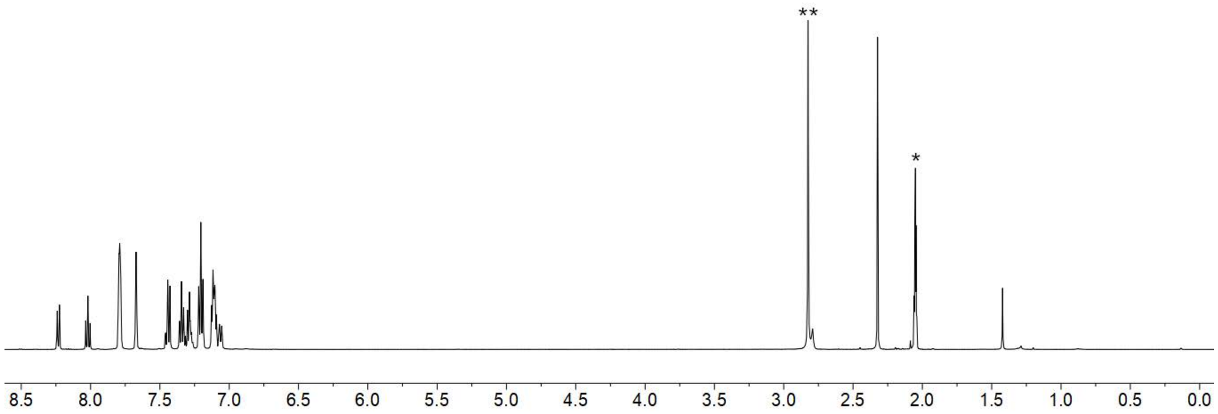

Fig. S54 ^{1}H NMR spectrum of [Cu(POP)(Me_2bpy)][BAr$^F_4$] (500 MHz, 298 K, acetone-d6). * = residual acetone-d_5; ** = H_2O and HDO.

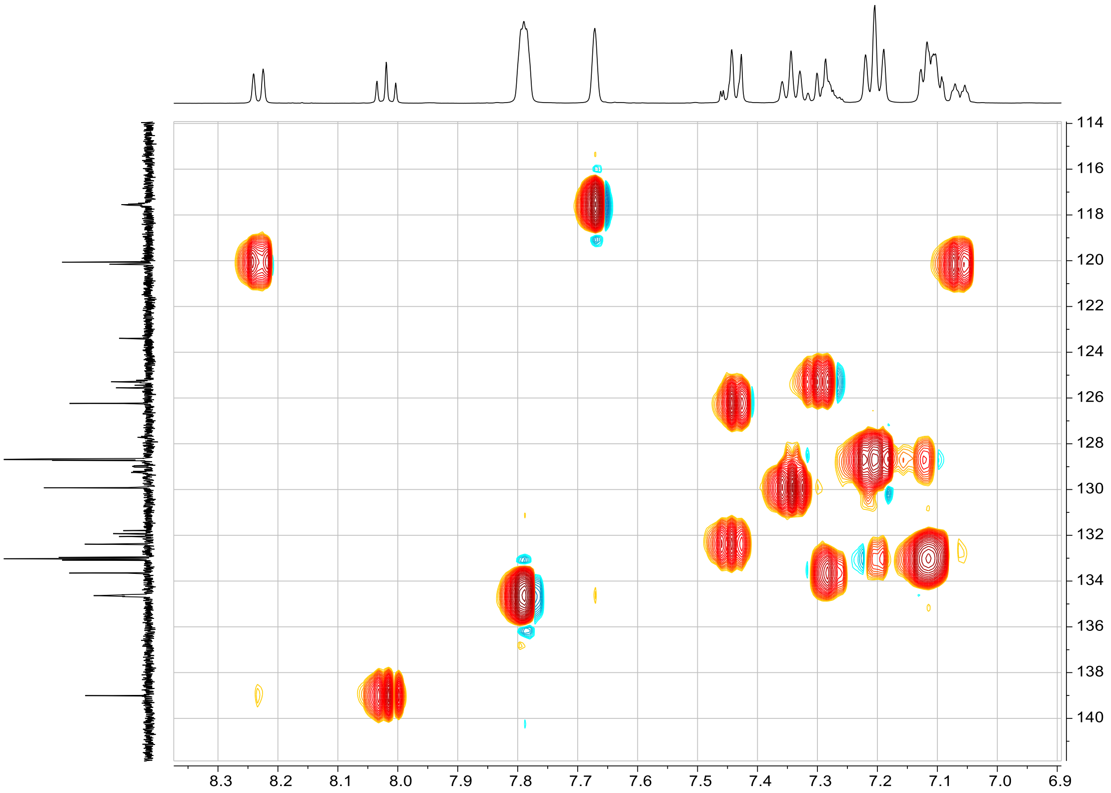

Fig. S55 The aromatic region of the HMQC spectrum (500 MHz ^{1}H, 126 MHz ^{13}C{^{1}H}, acetone-d_6, 298 K) of [Cu(POP)(Me_2bpy)][BAr$^F_4$]. Scale: δ / ppm.

This journal is © The Royal Society of Chemistry 20xx

Please do not adjust margins

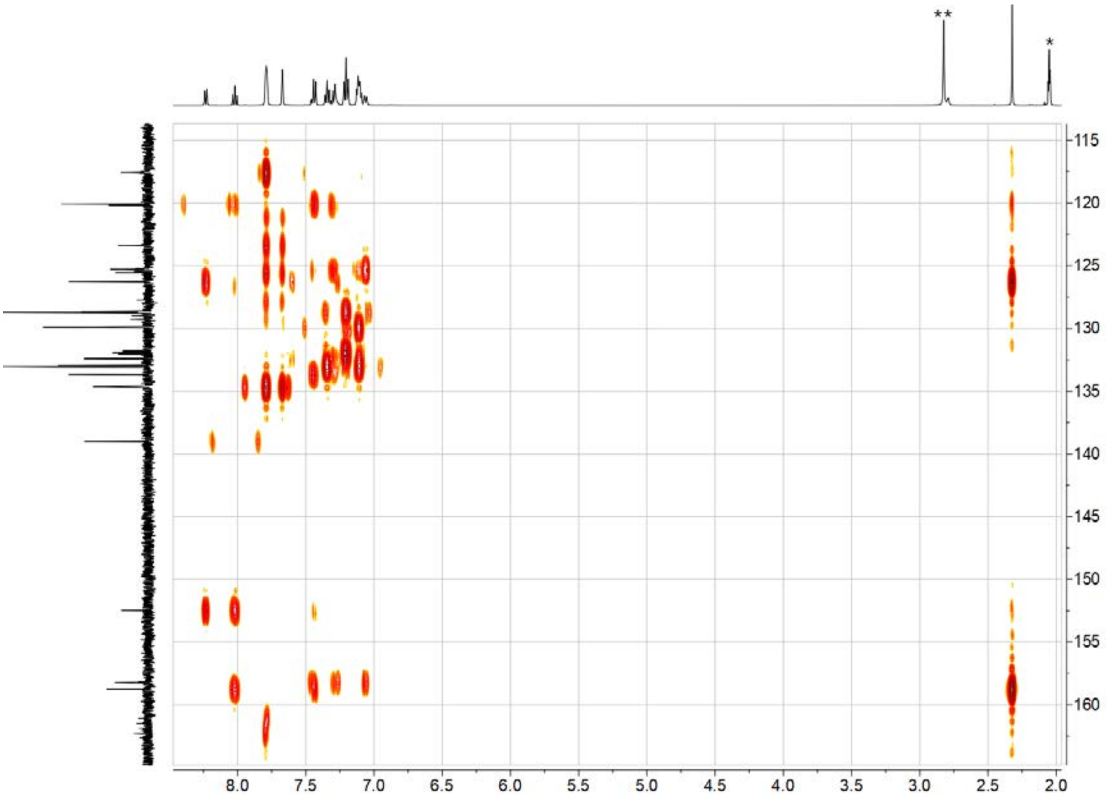

Fig. S56 Part of the HMBC spectrum (500 MHz 1H, 126 MHz $^{13}C\{^{1}H\}$, acetone-d_6, 298 K) of [Cu(POP)(Me_2bpy)][$BAr^F{}_4$]. Scale: δ / ppm. ** = H_2O and HDO.

Please do not adjust margins

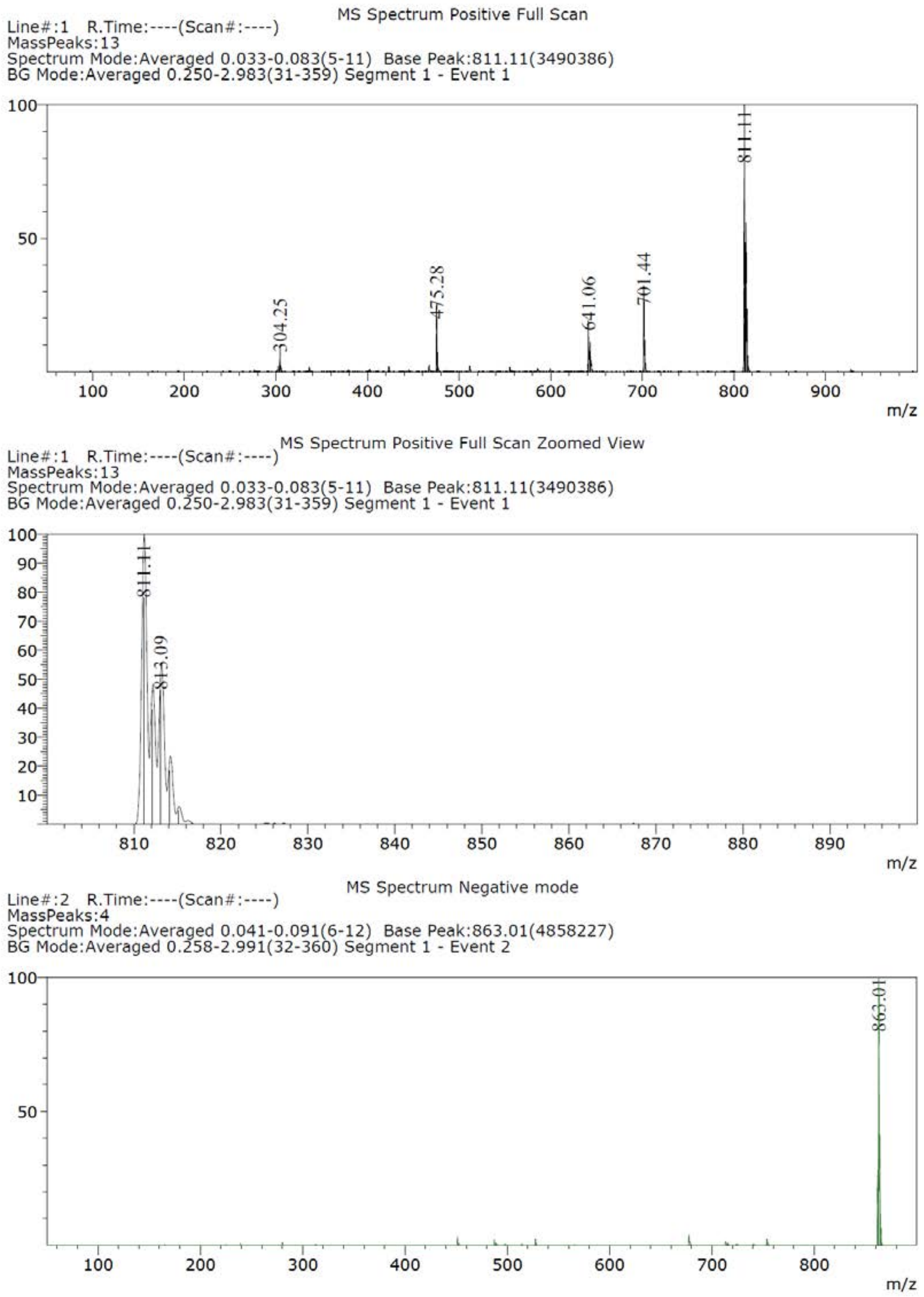

Fig. S57 The ESI mass spectrum (positive and negative mode) of [Cu(xantphos)(Mebpy)][BAr$^F{}_4$].

Please do not adjust margins

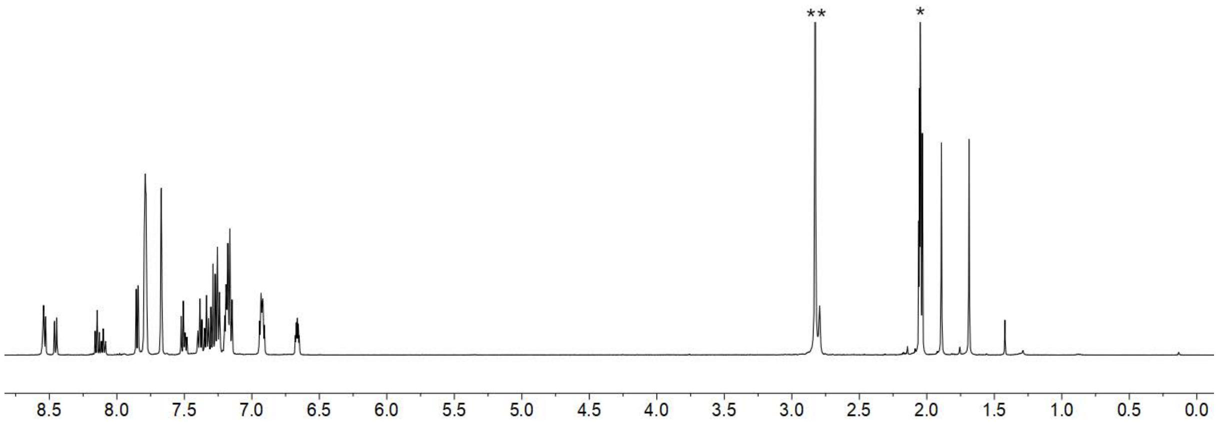

Fig. S58 ^1H NMR spectrum of [Cu(xantphos)(Mebpy)][BAr$^F{}_4$] (500 MHz, 298 K, acetone-d6). * = residual acetone-d_5; ** = H_2O and HDO.

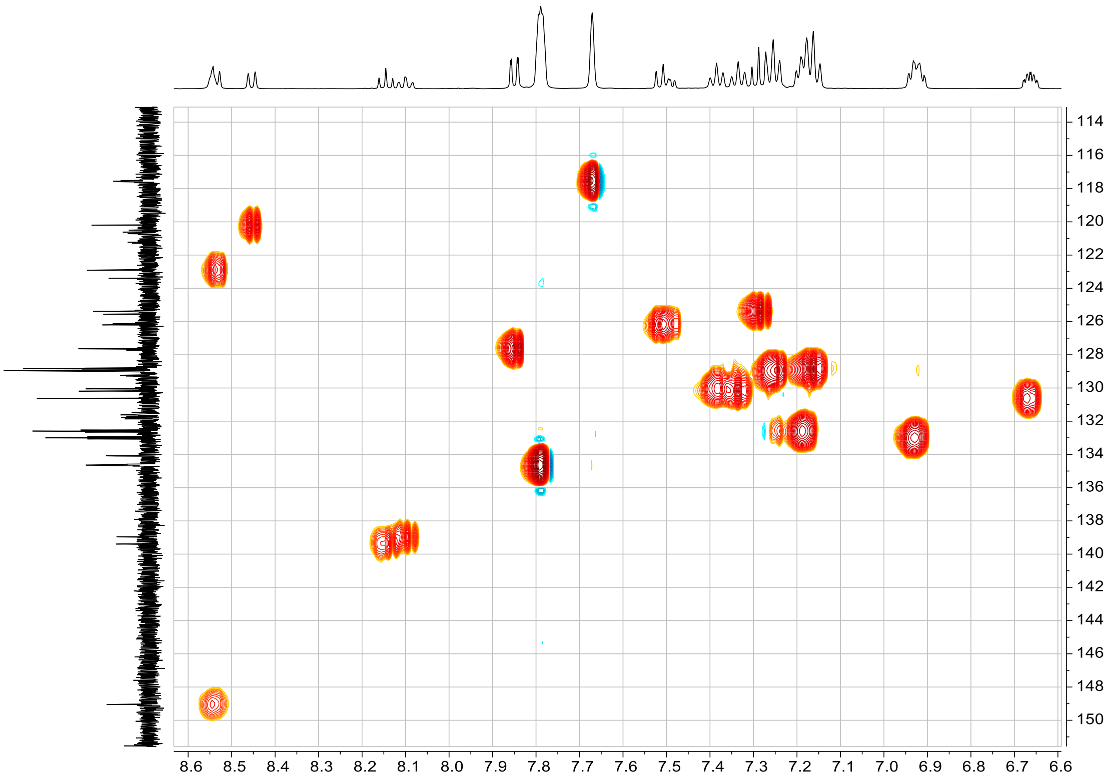

Fig. S59 The aromatic region of the HMQC spectrum (500 MHz ^1H, 126 MHz $^{13}C\{^1H\}$, acetone-d_6, 298 K) of [Cu(xantphos)(Mebpy)][BAr$^F{}_4$]. Scale: δ / ppm.

Please do not adjust margins

Please do not adjust margins

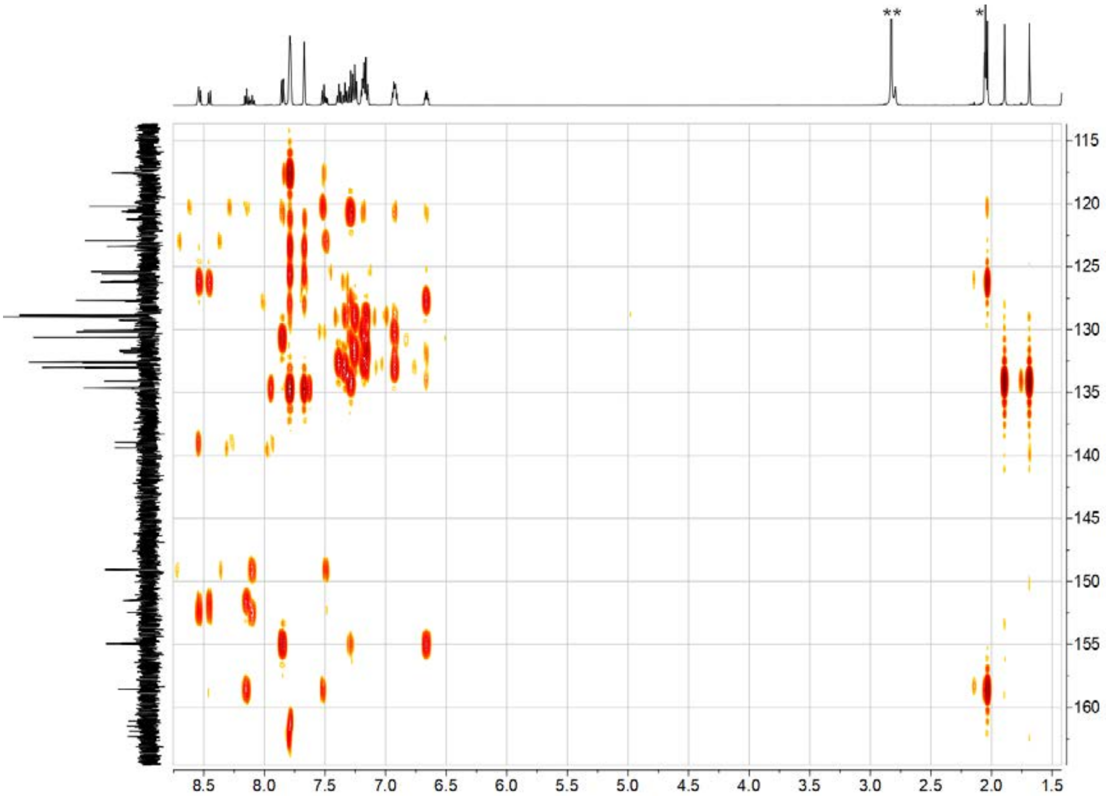

Fig. S60 Part of the HMBC spectrum (500 MHz 1H, 126 MHz $^{13}C\{^{1}H\}$, acetone-d_6, 298 K) of [Cu(xantphos)(Mebpy)][BAr$^F{}_4$]. Scale: δ / ppm. ** = H_2O and HDO.

 This journal is © The Royal Society of Chemistry 20xx

Please do not adjust margins

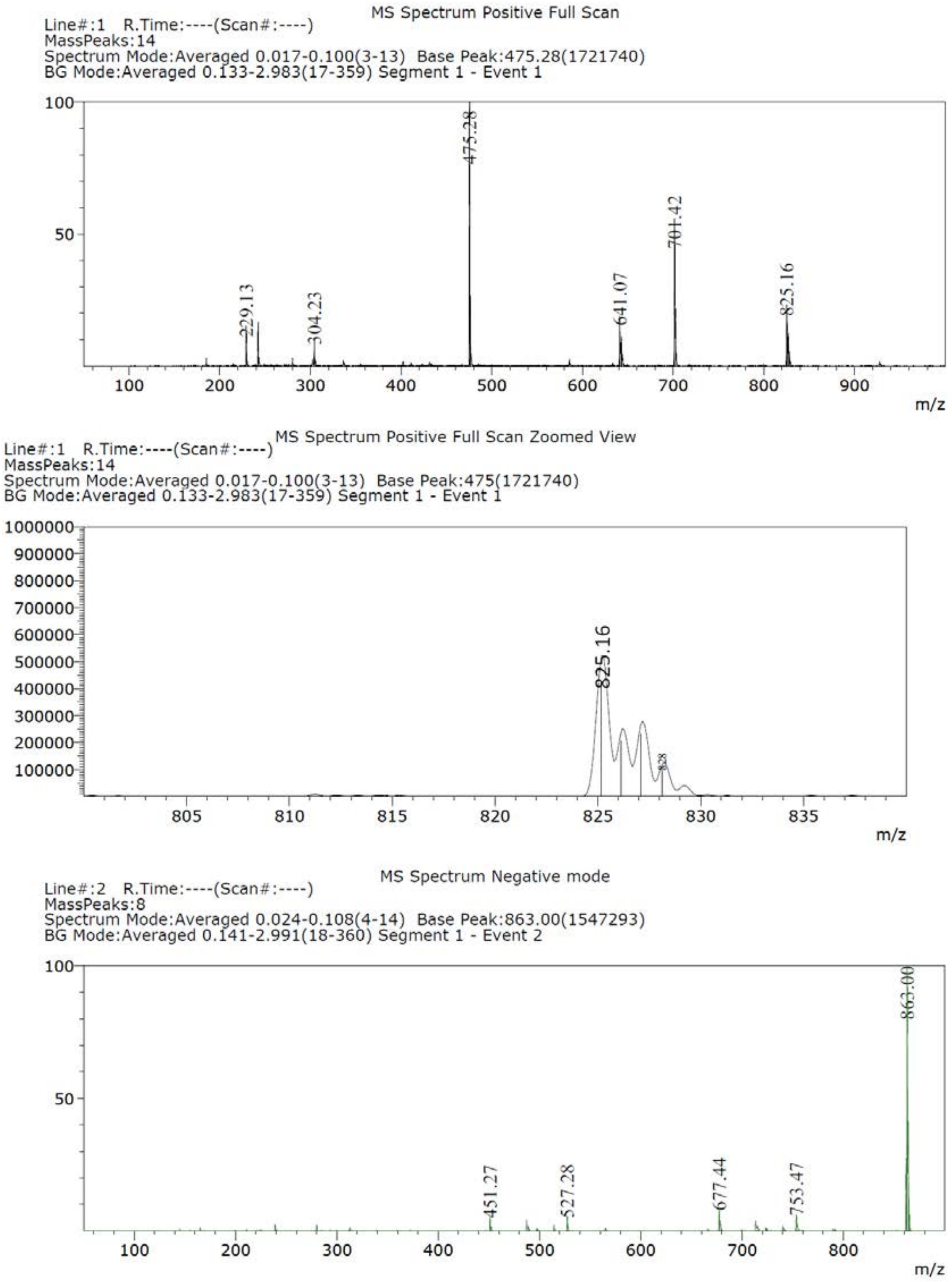

Fig. S61 The ESI mass spectrum (positive and negative mode) of [Cu(xantphos)(Me_2bpy)][BAr$^F{}_4$].

Please do not adjust margins

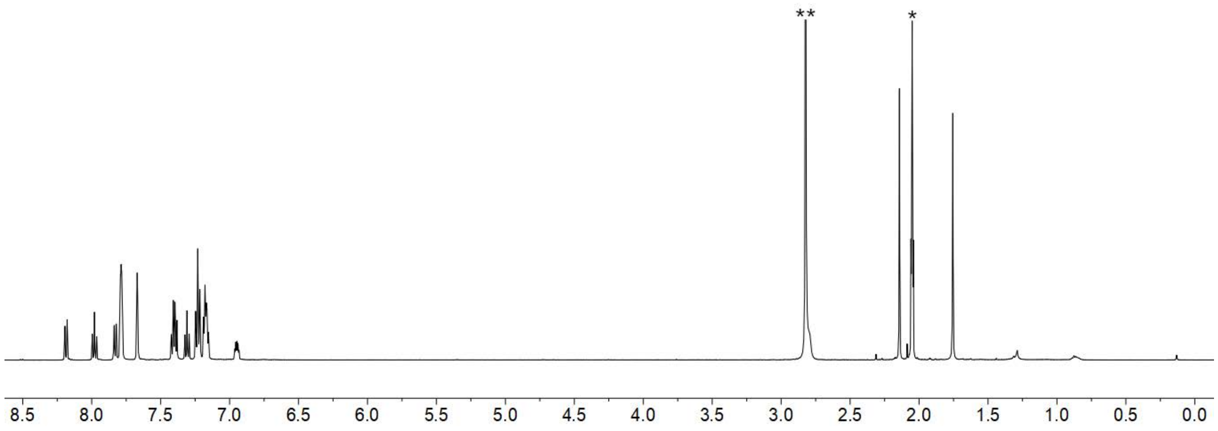

Fig. S62 ^{1}H NMR spectrum of [Cu(xantphos)(Me$_2$bpy)][BAr$^F{}_4$] (500 MHz, 298 K, acetone-d6). * = residual acetone-d_5; ** = H_2O and HDO.

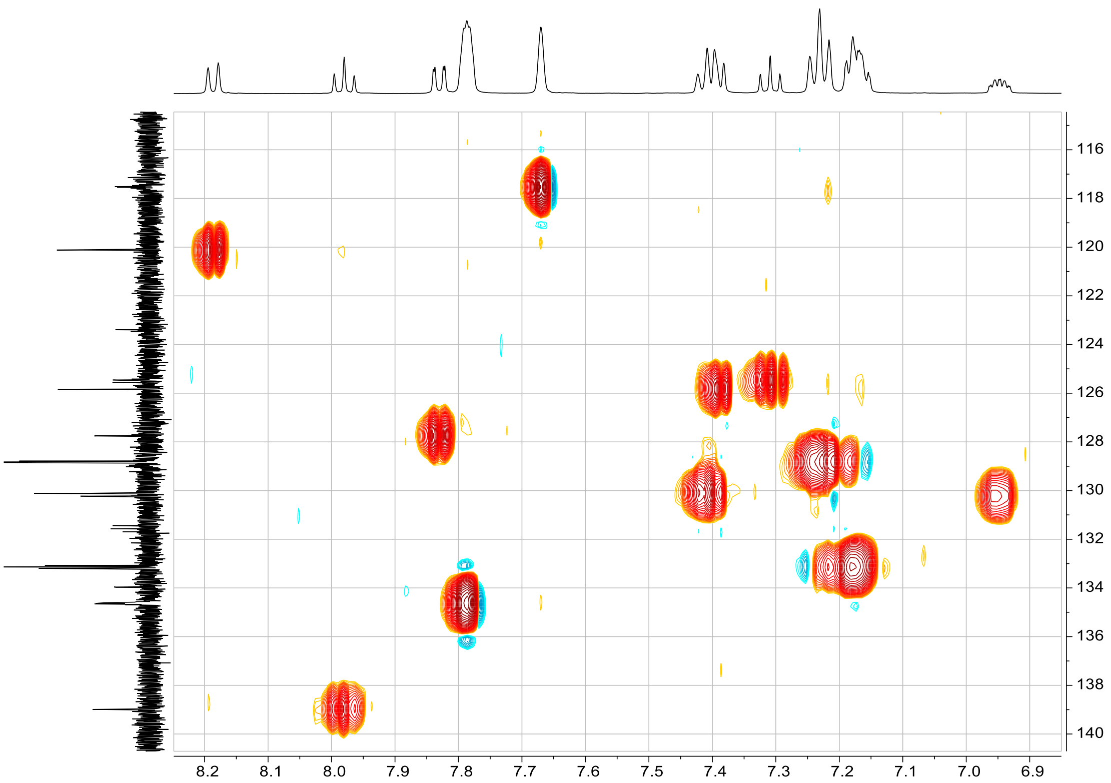

Fig. S63 The aromatic region of the HMQC spectrum (500 MHz ^{1}H, 126 MHz $^{13}C\{^{1}H\}$, acetone-d_6, 298 K) of [Cu(xantphos)(Me$_2$bpy)][BAr$^F{}_4$]. Scale: δ / ppm.

This journal is © The Royal Society of Chemistry 20xx

Please do not adjust margins

Please do not adjust margins

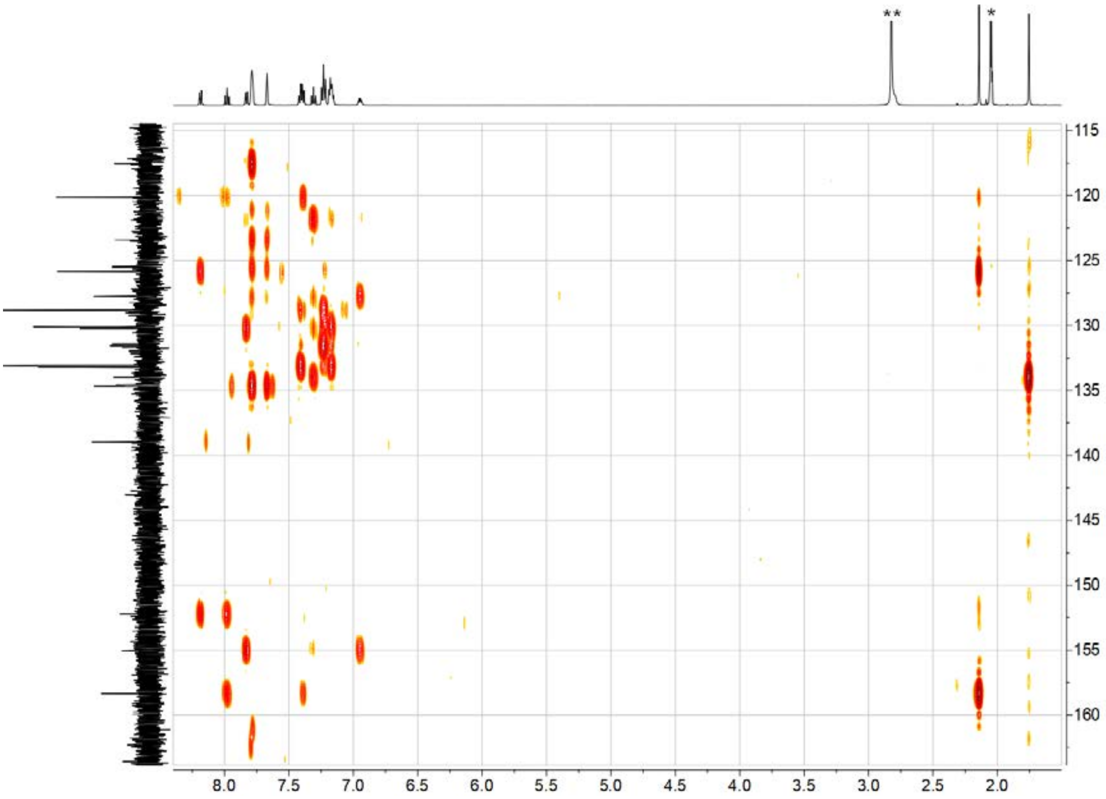

Fig. S64. Part of the HMBC spectrum (500 MHz 1H, 126 MHz $^{13}C\{^{1}H\}$, acetone-d_6, 298 K) of [Cu(xantphos)(Me$_2$bpy)][BAr$^F{}_4$]. Scale: δ / ppm. ** = H_2O and HDO.

Please do not adjust margins

Please do not adjust margins

Table S1 Crystallographic data for the Cu(I) complexes

Compound	[Cu(POP)(Mebpy)][PF_6]·0.5CH_2Cl_2·0.3Et_2O	[Cu(xantphos)(Mebpy)][PF_6]·0.5CH_2Cl_2·Et_2O	[Cu(xantphos)(Mebpy)][BF_4]·CH_2Cl_2· Et_2O
Formula	$C_{48.70}H_{41.70}ClCuF_6N_2O_{1.30}P_3$	$C_{54.50}H_{53}ClCuF_6N_2O_2P_3$	$C_{55}H_{54}BCl_2CuF_4N_2O_2P_2$
Formula weight	981.64	1073.89	1058.19
Crystal colour and habit	Yellow block	Yellow block	Yellow block
Crystal system	Monoclinic	Triclinic	Triclinic
Space group	$P2_1/n$	$P\bar{1}$	$P\bar{1}$
a, b, c / Å	9.7279(9), 16.4029(15), 29.365(3)	11.0667(8), 15.0405(11), 18.1887(14)	10.8320(5), 14.9163(6), 18.2441(8)
α, β, γ / °	90, 96.379(2), 90	109.125(2), 96.617(2), 109.348(2)	109.7369(15), 93.2992(17), 110.6852(16)
U / $Å^3$	4656.6(7)	2612.4(3)	2542.84(19)
Dc / Mg m^{-3}	1.400	1.365	1.382
Z	4	2	2
Radiation type	Cu-Kα	Cu-Kα	Cu-Kα
μ / mm^{-1}	2.704	2.467	2.649
T / K	150	150	150
Refln. collected (R_{int})	27579 (0.0258)	33044(0.0307)	33057 (0.0267)
Unique refln.	8442	9401	9409
Refln. for refinement	8258	9314	8707
Parameters	615	645	547
Threshold	$I > 2\sigma(I)$	$I > 2\sigma(I)$	$I > 2\sigma(I)$
*R*1 (*R*1 all data)	0.0534 (0.0541)	0.0569 (0.0573)	0.0610 (0.0645)
*wR*2 (*wR*2 all data)	0.1426 (0.1431)	0.1806 (0.1813)	0.1758 (0.1805)
Goodness of fit	1.152	1.078	1.042
CCDC deposition number	2081386	2081394	2081392
Compound	[Cu(xantphos)(Me_2bpy)][PF_6]	[Cu(xantphos)(Me_2bpy)][BF_4]·0.5C_6H_{12}·0.8Me_2CO	[Cu(xantphos)(Me_2bpy)][BPh_4]·0.7Me_2CO
Formula	$C_{51}H_{44}CuF_6N_2OP_3$	$C_{56.4}H_{54.8}BCuF_4N_2O_{1.8}P_2$	$C_{77.10}H_{68.20}BCuN_2O_{1.70}P_2$
Formula weight	971.33	1001.71	1186.22
Crystal colour and habit	Yellow block	Yellow block	Yellow plate
Crystal system	Orthorhombic	Triclinic	Orthorhombic
Space group	$P2_12_12_1$	$P\bar{1}$	$Pna2_1$
a, b, c / Å	19.7640(3), 19.7573(3), 22.6092(4)	11.0536(7), 14.3637(9), 18.0991(12)	28.7566(8), 14.3706(4), 30.3845(10)
α, β, γ / °	90, 90, 90	67.326(3), 77.613(3), 85.130(3)	90, 90, 90
U / $Å^3$	8828.5(2)	2589.8(3)	12556.4(6)
Dc / Mg m^{-3}	1.462	1.285	1.255
Z	8	2	8
Radiation type	Ga-Kα	Cu-Kα	Cu-Kα
μ / mm^{-1}	3.715	1.642	1.345
T / K	150	150	150
Refln. collected (R_{int})	55800 (0.0882)	27941 (0.0277)	48359 (0.0895)
Unique refln.	17467	9439	18870
Refln. for refinement	13131	8700	15392
Parameters	1162	587	1507
Threshold	$I > 2\sigma(I)$	$I > 2\sigma(I)$	$I > 2\sigma(I)$
*R*1 (*R*1 all data)	0.0670 (0.1002)	0.0583 (0.0613)	0.0592 (0.0764)
*wR*2 (*wR*2 all data)	0.1495 (0.1668)	0.1805 (0.1842)	0.1389 (0.1509)
Goodness of fit	1.022	1.047	1.009
Flack parameter	0.370(6)		0.10(3)
CCDC deposition number	2081393	2081391	2081387

This journal is © The Royal Society of Chemistry 20xx

Please do not adjust margins

Compound	[Cu(xantphos)(Me_2bpy)][BAr^F_4]	[Cu(POP)(Me_2bpy)][BAr^F_4]	[Cu(POP)(Mebpy)][BAr^F_4]·C_6H_{12}
Formula	$C_{83}H_{56}BCuF_{24}N_2OP_2$	$C_{80}H_{52}BCuF_{24}N_2OP_2$	C85 H62 B Cu F24 N2 O P2
Formula weight	1689.58	1649.52	1719.65
Crystal colour and habit	Yellow block	Yellow block	Yellow plate
Crystal system	Triclinic	Triclinic	Monoclinic
Space group	$P\bar{1}$	$P\bar{1}$	$P2_1/c$
a, b, c / Å	10.7126(2), 18.8554(4), 19.4247(4)	13.4775(10), 13.7430(11), 20.8898(16)	14.2327(4), 30.8018(9), 18.4314(5)
α, β, γ / °	79.4640(10), 89.642(2), 74.7590(10)	89.276(3), 76.079(3), 85.590(3)	90, 94.048(2), 90
U / Å^3	3718.16(13)	3744.4(5)	8060.0(4)
Dc / Mg m^{-3}	1.509	1.463	1.417
Z	2	2	4
Radiation type	Ga-Kα	Cu-Kα	Cu-Kα
μ / mm^{-1}	2.475	1.768	1.665
T / K	150	150	150
Refln. collected (R_{int})	52347 (0.0686)	34371 (0.0266)	58689 (0.0377)
Unique refln.	14623	13675	12748
Refln. for refinement	13290	12292	14521
Parameters	1037	1002	962
Threshold	$I > 2\sigma(I)$	$I > 2\sigma(I)$	$I > 2\sigma(I)$
$R1$ ($R1$ all data)	0.0644 (0.0693)	0.0633 (0.0689)	0.0865 (0.0953)
$wR2$ ($wR2$ all data)	0.1716 (0.1760)	0.1633 (0.1692)	0.2089 (0.2156)
Goodness of fit	1.039	1.051	1.057
CCDC deposition number	2081388	2081389	2081390

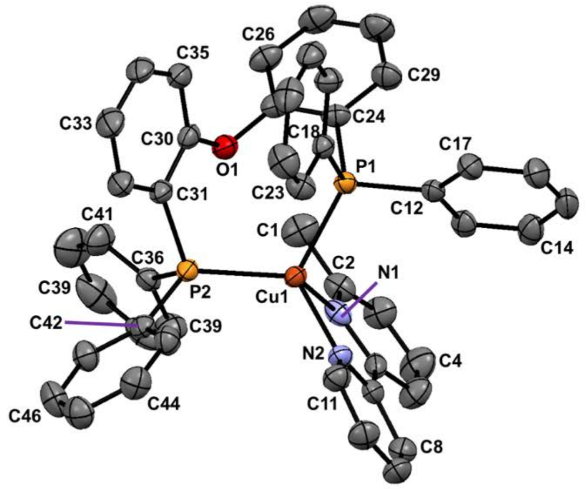

Fig. S65 Structure of the [Cu(POP)(Mebpy)]$^+$ cation in [Cu(POP)(Mebpy)][PF_6]·0.5CH_2Cl_2·0.5Et_2O. H atoms and solvent molecules are omitted and ellipsoids are plotted at 50% probability level. Selected bond parameters:

Cu1–P2 = 2.2528(8),
Cu1–P1 = 2.2736(8),
Cu1–N2 = 2.051(2),
Cu1–N1 = 2.118(2),
C25–O1 = 1.397(4),
C30–O1 = 1.380(4) Å;
P2–Cu1–P1 = 112.93(3),
P2–Cu1–N2 = 118.39(7),
P1–Cu1–N2 = 113.20(7),
P2–Cu1–N1 = 119.44(7),
P1–Cu1–N1 = 108.74(7),
N2–Cu1–N1 = 80.11(9),
C25–O1–C30 = 119.0(2) °.

Please do not adjust margins

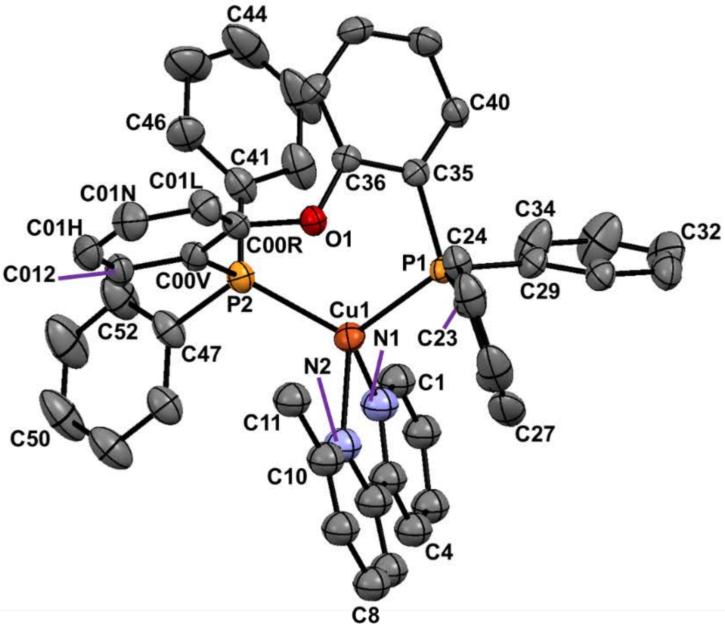

Fig. S66 Structure of the [Cu(POP)(Mebpy)]+ cation in [Cu(POP)(Mebpy)][BAr$^F{}_4$]·C_6H_{12}. H atoms and solvent molecules are omitted and ellipsoids are plotted at 50% probability level. Selected bond parameters:

Molecule 1
Cu1–P2 = 2.254(1),
Cu1–P1 = 2.272(1),
Cu1–N2 = 2.163(6),
Cu1–N1 = 2.028(6),
C36–O1 = 1.385(5);
C00R–O1 = 1.397(5) Å;
P2–Cu1–P1 = 115.43(4),
P2–Cu1–N2 = 115.2(2),
P1–Cu1–N2 = 111.2(2),
P2–Cu1–N1 = 115.9(2),
P1–Cu1–N1 = 114.8(2),
N2–Cu1–N1 = 79.1(2),
C36–O1–C00R = 121.5(3) °.
Molecule 2
Cu1–P2 = 2.254(1),
Cu1–P1 = 2.272(1),
Cu1–N4 = 1.983(6),
Cu1–N3 = 2.049(6),
C36–O2 = 1.385(5),
C00R–O2 = 1.397(5) Å;
P2–Cu1–P1 = 115.43(4),
P2–Cu1–N4 = 109.7(2),
P1–Cu1–N4 = 106.6(2),
P2–Cu1–N3 = 120.1(2),
P1–Cu1–N3 = 116.7(2),
N4–Cu1–N3 = 81.4(3),
C36–O2–C00R = 121.5(3) °.

 This journal is © The Royal Society of Chemistry 20xx

Please do not adjust margins

Please do not adjust margins

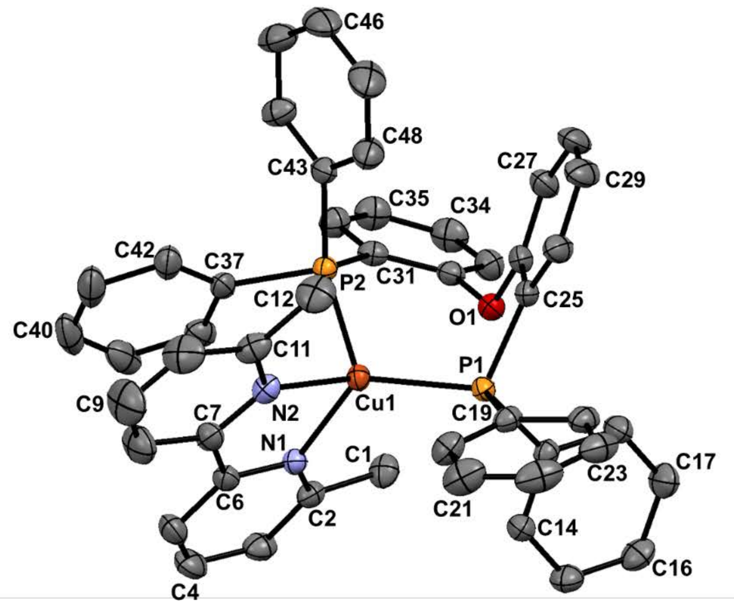

Fig. S67 Structure of the $[Cu(POP)(Me_2bpy)]^+$ cation in $[Cu(POP)(Me_2bpy)][BAr^F_4]$. H atoms and solvent molecules are omitted and ellipsoids are plotted at 50% probability level. Selected bond parameters:

Cu1–P2 = 2.3185(7),
Cu1–P1 = 2.2296(8),
Cu1–N2 = 2.089(3),
Cu1–N1 = 2.068(3),
C26–O1 = 1.387(4),
C32–O1 = 1.395(3) Å;
P2–Cu1–P1 = 115.92(3),
P2–Cu1–N2 = 103.13(7),
P1–Cu1–N2 = 125.13(7),
P2–Cu1–N1 = 100.75(7),
P1–Cu1–N1 = 124.41(7),
N2–Cu1–N1 = 80.5(1),
C26–O1–C32 = 118.5(2) °.

This journal is © The Royal Society of Chemistry 20xx

Please do not adjust margins

Please do not adjust margins

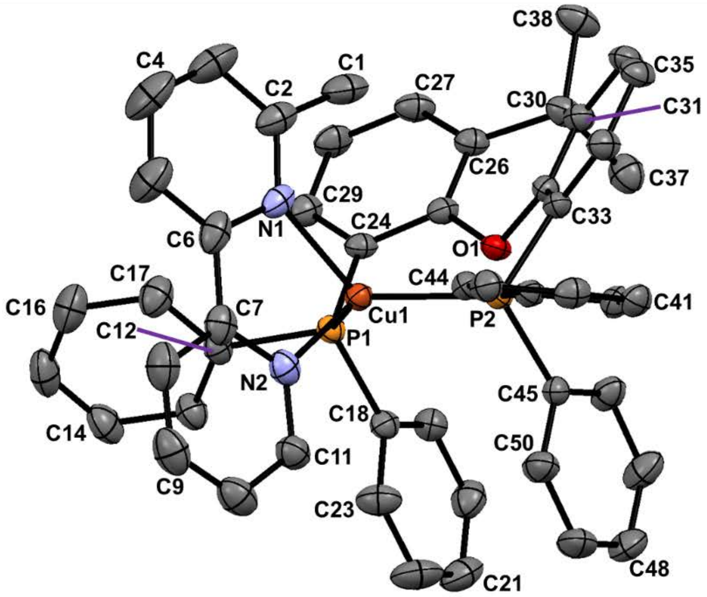

Fig. S68 Structure of the [Cu(xantphos)(Mebpy)]$^+$ cation in [Cu(xantphos)(Mebpy)][PF_6]·CH_2Cl_2·Et_2O. H atoms and solvent molecules are omitted and ellipsoids are plotted at 50% probability level. Selected bond parameters:
Cu1–P2 = 2.2509(9),
Cu1–P1 = 2.2677(8),
Cu1–N2 = 2.040(3),
Cu1–N1 = 2.074(4),
C25–O1 = 1.388(4),
C32–O1 = 1.397(4) Å;
P2–Cu1–P1 = 113.44(3),
P2–Cu1–N2 = 114.95(9),
P1–Cu1–N2 = 111.21(9),
P2–Cu1–N1 = 121.03(9),
P1–Cu1–N1 = 111.30(9),
N2–Cu1–N1 = 80.8(1),
C25–O1–C32 = 114.2(2) °.

This journal is © The Royal Society of Chemistry 20xx

Please do not adjust margins

Please do not adjust margins

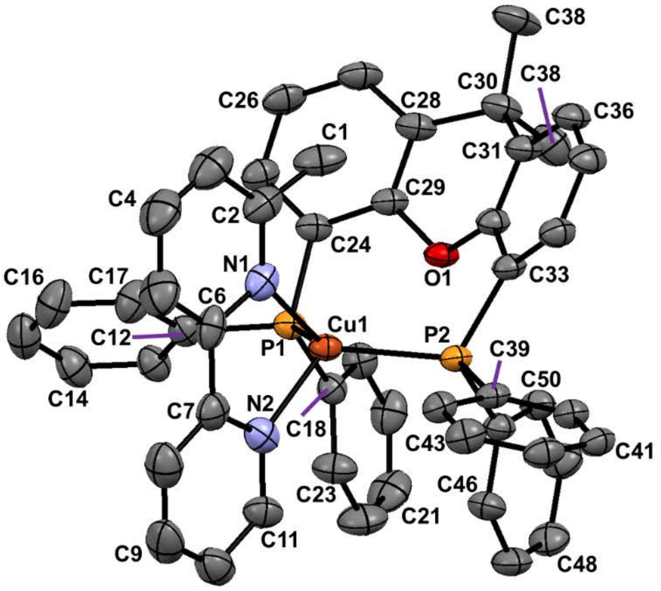

Fig. S69 Structure of the $[Cu(xantphos)(Mebpy)]^+$ cation in $[Cu(xantphos)(Mebpy)][BF_4]$. H atoms and solvent molecules are omitted and ellipsoids are plotted at 50% probability level. Selected bond parameters:
Cu1–P2 = 2.2559(9),
Cu1–P1 = 2.2654(8),
Cu1–N2 = 2.045(2),
Cu1–N1 = 2.072(4),
C29–O1 = 1.389(3),
C32–O1 = 1.399(4) Å;
P2–Cu1–P1 = 113.34(3),
P2–Cu1–N2 = 114.83(9),
P1–Cu1–N2 = 111.77(9),
P2–Cu1–N1 = 119.20(9),
P1–Cu1–N1 = 112.66(9),
N2–Cu1–N1 = 81.1(1),
C29–O1–C32 = 113.9(2) °.

This journal is © The Royal Society of Chemistry 20xx

Please do not adjust margins

Please do not adjust margins

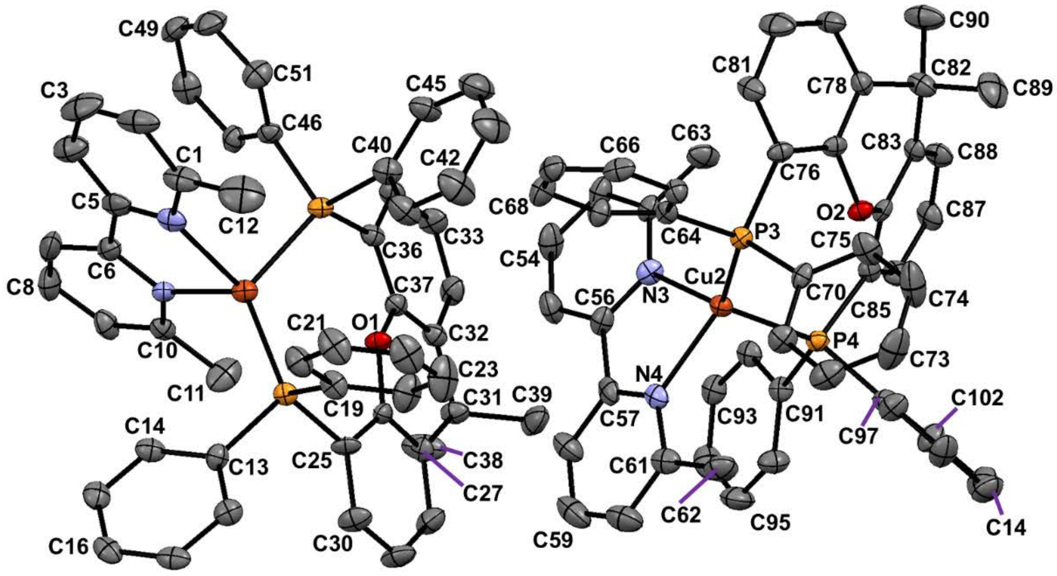

Fig. S70 Structure of the $[Cu(xantphos)(Me_2bpy)]^+$ cations in $[Cu(xantphos)(Me_2bpy)][PF_6]$. H atoms and solvent molecules are omitted and ellipsoids are plotted at 50% probability level. Selected bond parameters:
Molecule 1
Cu1–P2 = 2.323(2),
Cu1–P1 = 2.280(2),
Cu1–N2 = 2.087(6),
Cu1–N1 = 2.142(7),
C26–O1 = 1.369(8);
C37–O1 = 1.392(9) Å;
P2–Cu1–P1 = 121.53(8),
P2–Cu1–N2 = 101.7(2),
P1–Cu1–N2 = 120.5(2),
P2–Cu1–N1 = 97.5(2),
P1–Cu1–N1 = 126.9(2),
N2–Cu1–N1 = 79.1(2),
C26–O1–C37 = 118.7(6) °.
Molecule 2
Cu2–P4 = 2.320(2),
Cu2–P3 = 2.266(2),
Cu2–N4 = 2.127(7),
Cu2–N3 = 2.096(6),
C77–O2 = 1.401(8),
C84–O2 = 1.40(1) Å;
P4–Cu2–P3 = 117.77(8),
P4–Cu2–N4 = 99.9(2),
P3–Cu2–N4 = 129.3(2),
P4–Cu2–N3 = 102.9(2),
P3–Cu2–N3 = 119.8(2),
N4–Cu2–N3 = 79.0(2),
C77–O2–C84 = 115.0(6) °.

 This journal is © The Royal Society of Chemistry 20xx

Please do not adjust margins

Please do not adjust margins

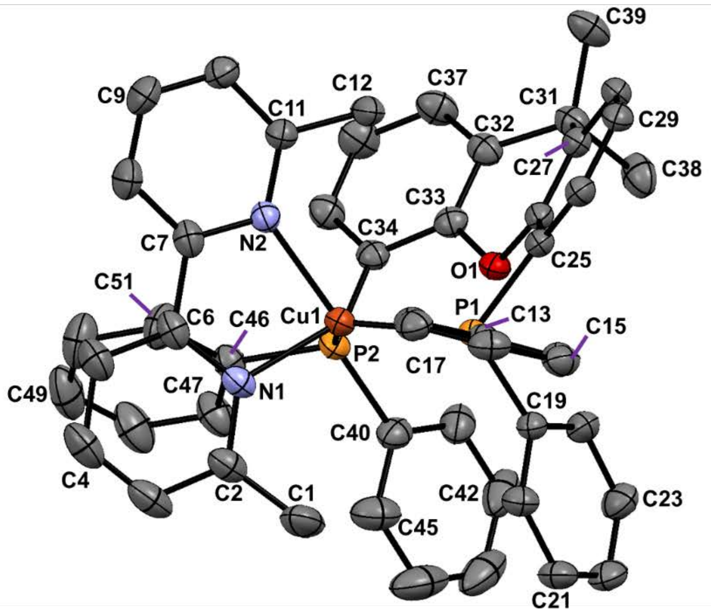

Fig. S71 Structure of the $[Cu(xantphos)(Me_2bpy)]^+$ cation in $[Cu(xantphos)(Me_2bpy)][BF_4]\cdot C_6H_{12}$. H atoms and solvent molecules are omitted and ellipsoids are plotted at 50% probability level. Selected bond parameters:
Cu1–P2 = 2.2988(7),
Cu1–P1 = 2.2695(9),
Cu1–N2 = 2.110(3),
Cu1–N1 = 2.091(2),
C26–O1 = 1.397(3),
C33–O1 = 1.387(4) Å;
P2–Cu1–P1 = 111.54(3),
P2–Cu1–N2 = 110.18(7),
P1–Cu1–N2 = 120.03(7),
P2–Cu1–N1 = 109.75(7),
P1–Cu1–N1 = 121.94(7),
N2–Cu1–N1 = 79.60(9),
C26–O1–C33 = 114.8(2) °.

This journal is © The Royal Society of Chemistry 20xx

Please do not adjust margins

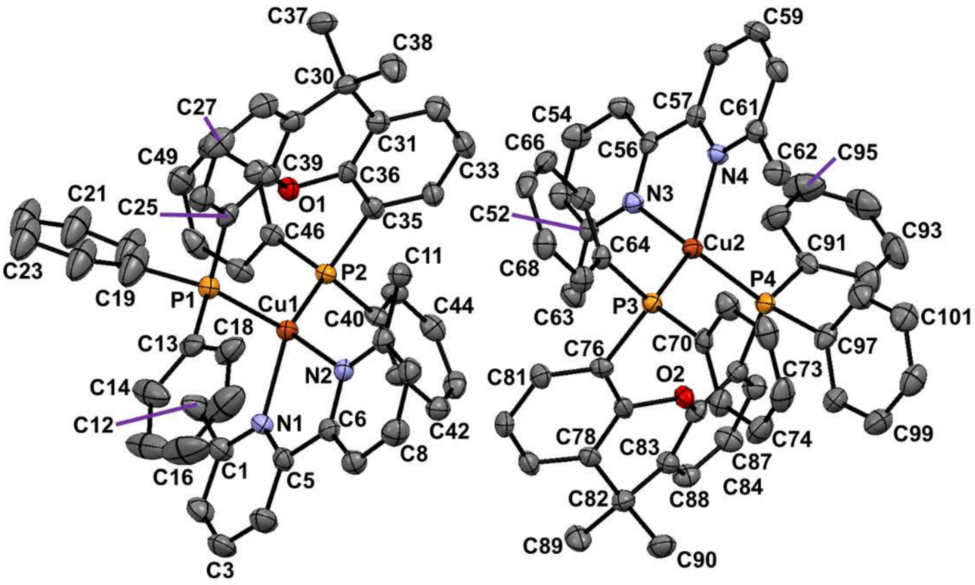

Fig. S72 Structure of the $[Cu(xantphos)(Me_2bpy)]^+$ cations in $[Cu(xantphos)(Me_2bpy)][BPh_4]\cdot 0.7C_3H_6O$. H atoms and solvent molecules are omitted and ellipsoids are plotted at 50% probability level. Selected bond parameters:

Molecule 1
Cu1–P2 = 2.255(2),
Cu1–P1 = 2.310(2),
Cu1–N2 = 2.129(5),
Cu1–N1 = 2.093(5),
C36–O1 = 1.377(7);
C39–O1 = 1.376(7) Å;
P2–Cu1–P1 = 117.99(7),
P2–Cu1–N2 = 123.1(2),
P1–Cu1–N2 = 100.7(2),
P2–Cu1–N1 = 124.1(1),
P1–Cu1–N1 = 103.8(1),
N2–Cu1–N1 = 79.3(2),
C36–O1–C39 = 118.5(5) °.

Molecule 2
Cu2–P4 = 2.306(2),
Cu2–P3 = 2.287(2),
Cu2–N4 = 2.122(5),
Cu2–N3 = 2.102(5),
C77–O2 = 1.397(7),
C84–O2 = 1.387(7) Å;
P4–Cu2–P3 = 113.48(7),
P4–Cu2–N4 = 113.3(1),
P3–Cu2–N4 = 118.1(1),
P4–Cu2–N3 = 108.5(2),
P3–Cu2–N3 = 120.1(2),
N4–Cu2–N3 = 78.9(2),
C77–O2–C84 = 115.9(4) °.

This journal is © The Royal Society of Chemistry 20xx

Please do not adjust margins

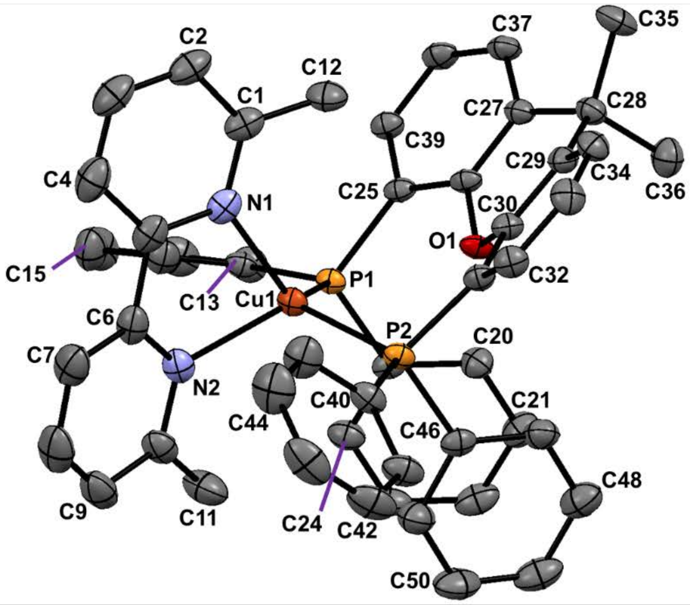

Fig. S73 Structure of the [Cu(xantphos)(Me_2bpy)]$^+$ cation in [Cu(xantphos)(Me_2bpy)][BAr^F_4]·C_6H_{12}. H atoms and solvent molecules are omitted and ellipsoids are plotted at 50% probability level. Selected bond parameters:
Cu1–P2 = 2.2914(7),
Cu1–P1 = 2.2871(8),
Cu1–N2 = 2.116(2),
Cu1–N1 = 2.122(2),
C26–O1 = 1.387(3),
C30–O1 = 1.386(3) Å;
P2–Cu1–P1 = 113.12(3),
P2–Cu1–N2 = 109.78(7),
P1–Cu1–N2 = 119.10(7),
P2–Cu1–N1 = 112.14(7),
P1–Cu1–N1 = 119.31(7),
N2–Cu1–N1 = 79.02(9),
C26–O1–C30 =116.6(2) °.

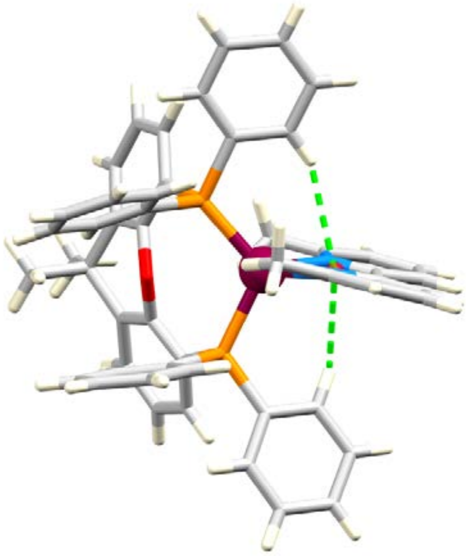

Fig. S74 One of the independent cations in [Cu(xantphos)(Me_2bpy)][BPh_4] features C–H...π contacts between one phenyl ring of each PPh_2 group and the bpy domain; the centroid of the chelate ring is shown in red.

Please do not adjust margins

Please do not adjust margins

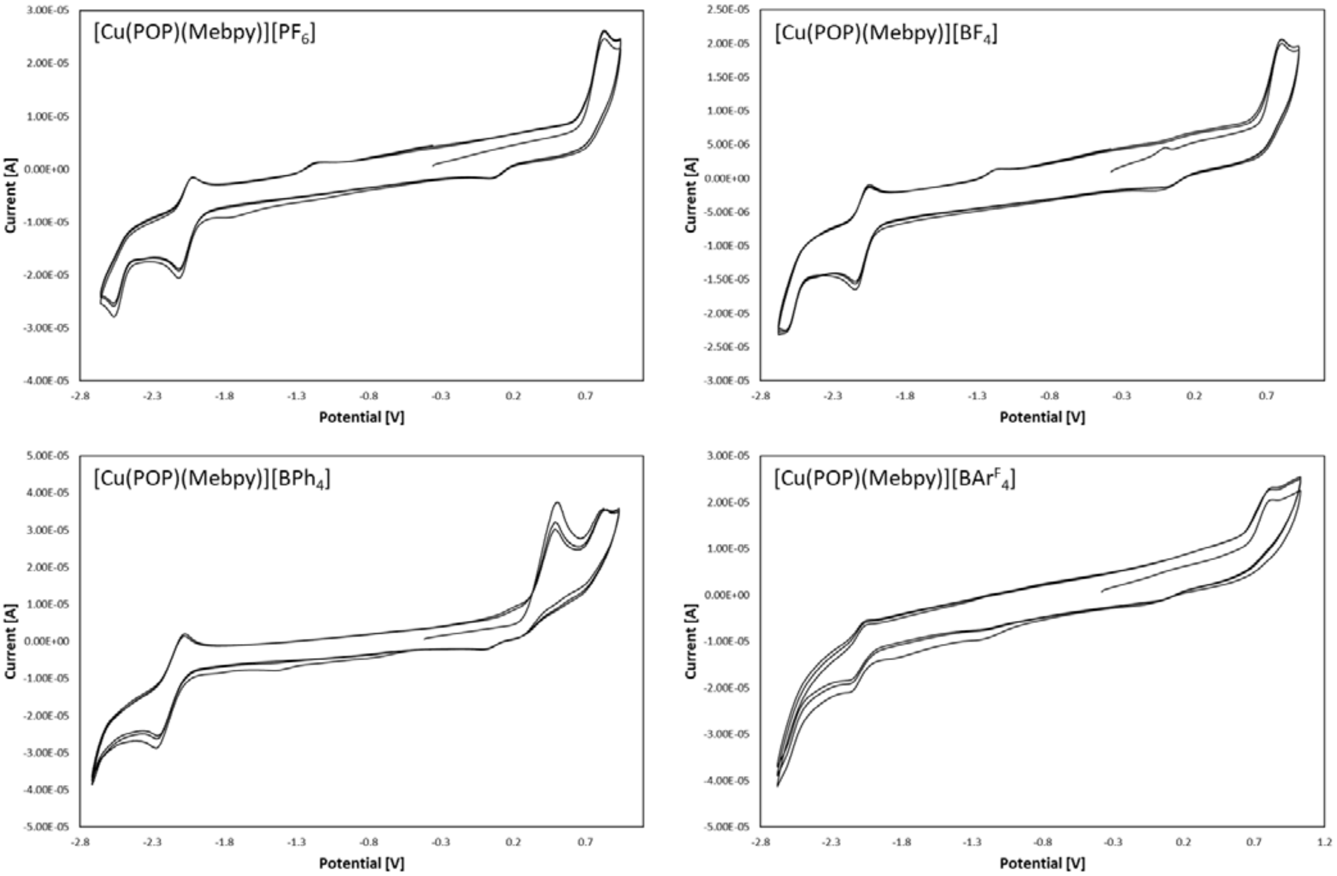

Fig. S75 Three consecutive scans in the cyclic voltammograms of [Cu(POP)(Mebpy)][A] in CH_2Cl_2 solution (ca. 10^{-4} mol dm^{-3}) with [$^nBu_4N$][PF_6] as supporting electrolyte and a scan rate of 0.1 V s^{-1} (referenced to internal Fc/Fc$^+$ = 0.0 V).

 This journal is © The Royal Society of Chemistry 20xx

Please do not adjust margins

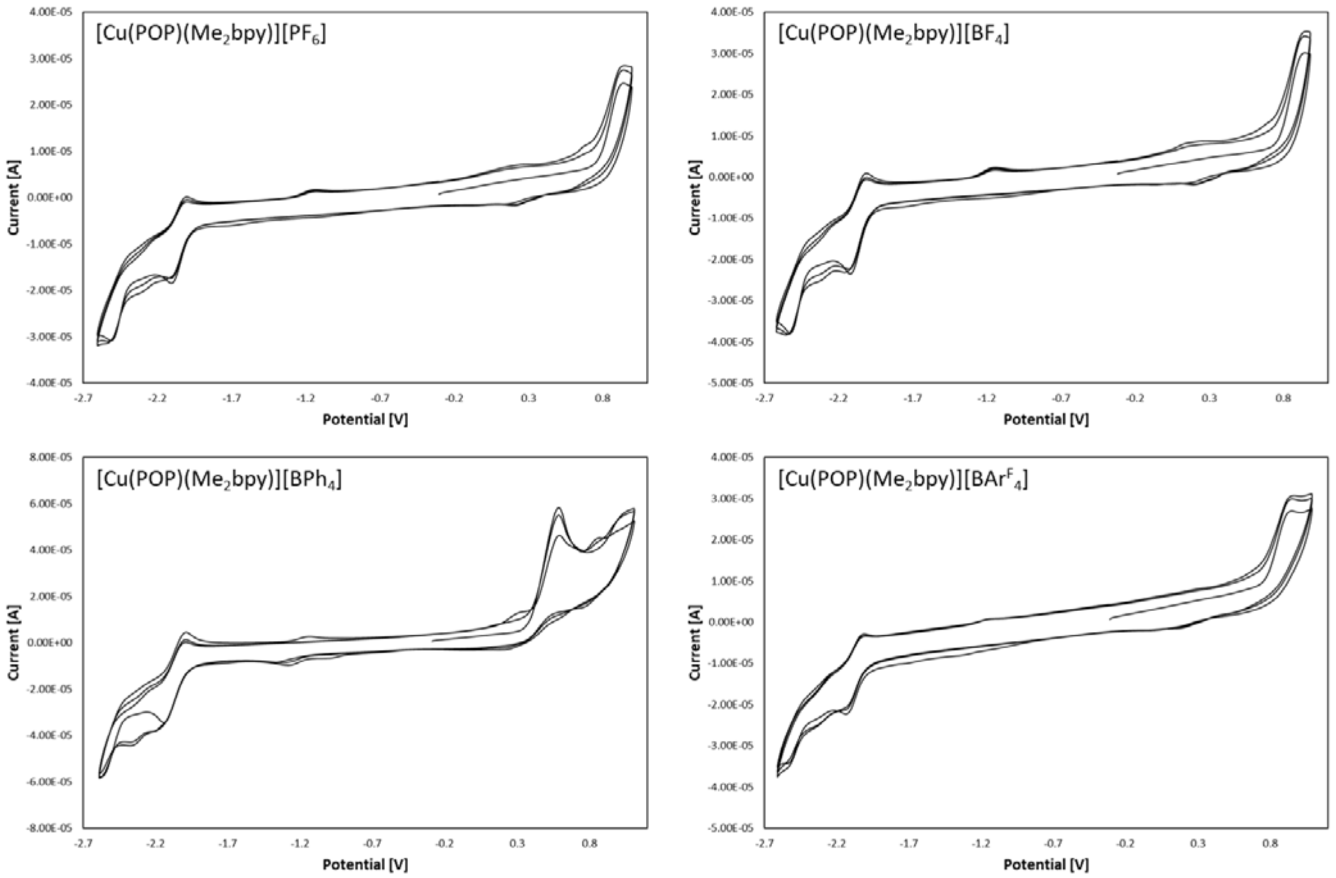

Fig. S76 Three consecutive scans in the cyclic voltammograms of [Cu(POP)(Me_2bpy)][A] in CH_2Cl_2 solution (ca. 10^{-4} mol dm^{-3}) with [$^{n}Bu_4N$][PF_6] as supporting electrolyte and a scan rate of 0.1 V s^{-1} (referenced to internal Fc/Fc^+ = 0.0 V).

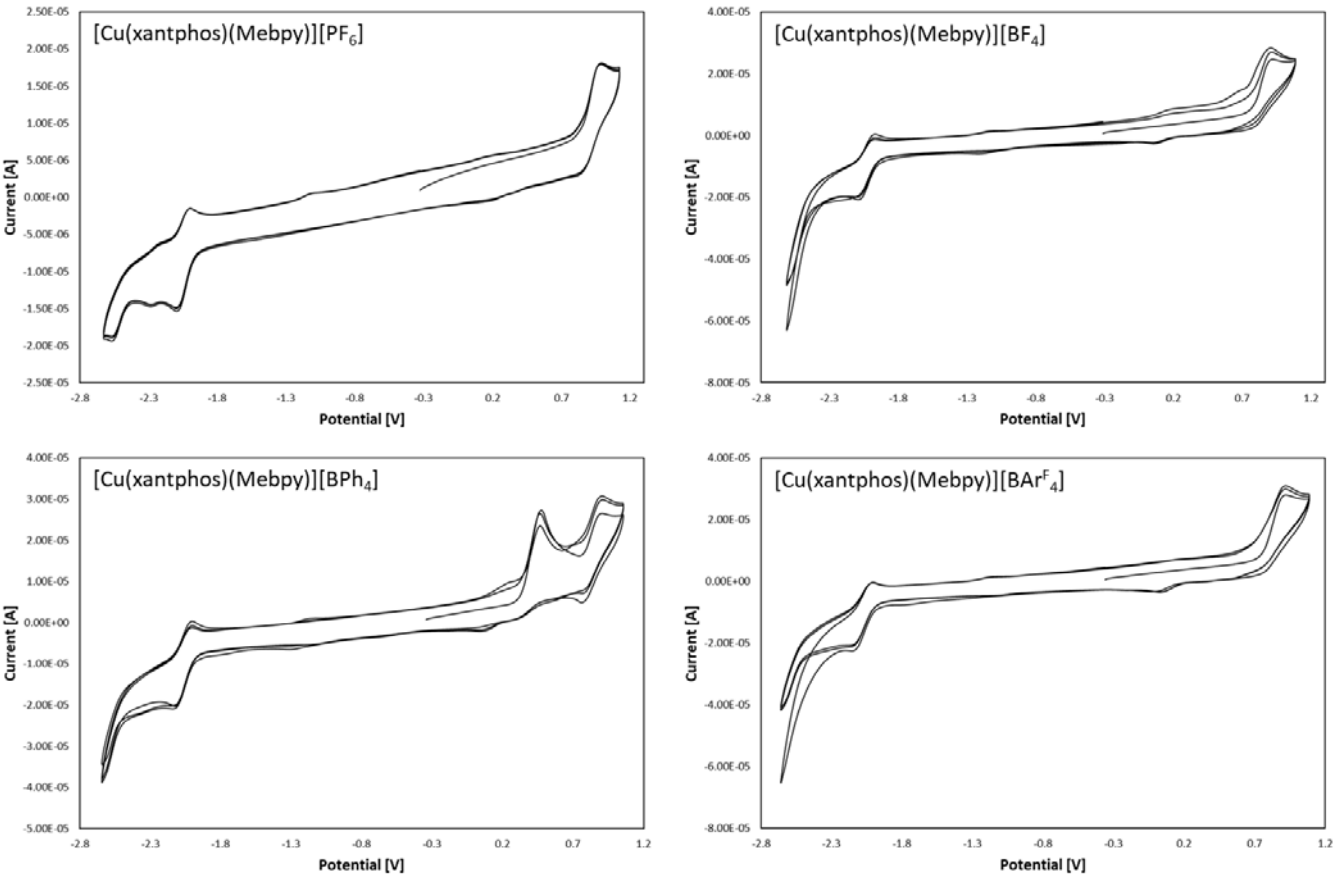

Fig. S77 Three consecutive scans in the cyclic voltammograms of [Cu(xantphos)(Mebpy)][A] in CH_2Cl_2 solution (ca. 10^{-4} mol dm^{-3}) with [$^nBu_4N$][PF_6] as supporting electrolyte and a scan rate of 0.1 V s^{-1} (referenced to internal Fc/Fc^+ = 0.0 V).

This journal is © The Royal Society of Chemistry 20xx

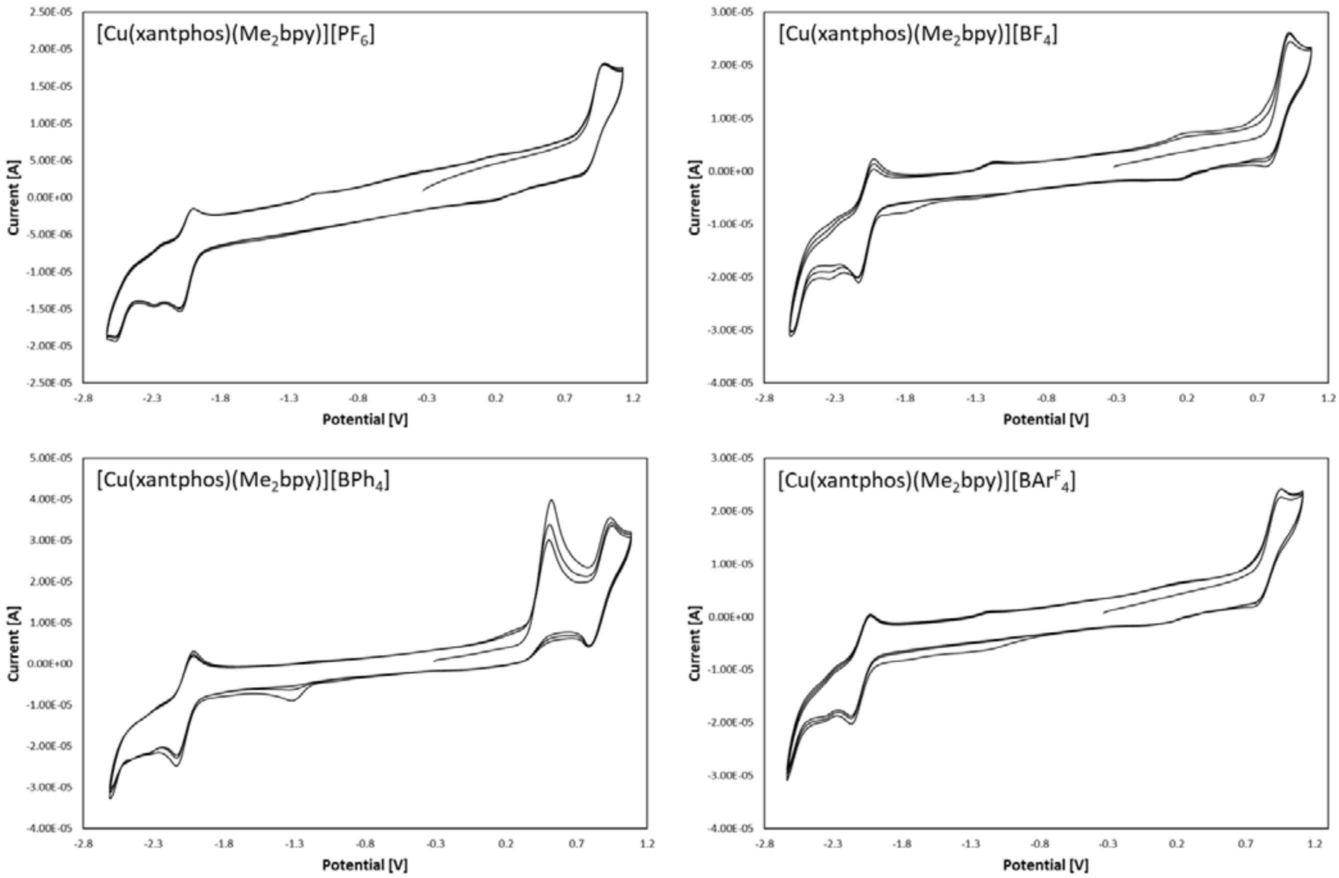

Fig. S78 Three consecutive scans in the cyclic voltammograms of [Cu(xantphos)(Me_2bpy)][A] in CH_2Cl_2 solution (ca. 10^{-4} mol dm^{-3}) with [^{n}Bu$_4$N][PF_6] as supporting electrolyte and a scan rate of 0.1 V s^{-1} (referenced to internal Fc/Fc$^+$ = 0.0 V).

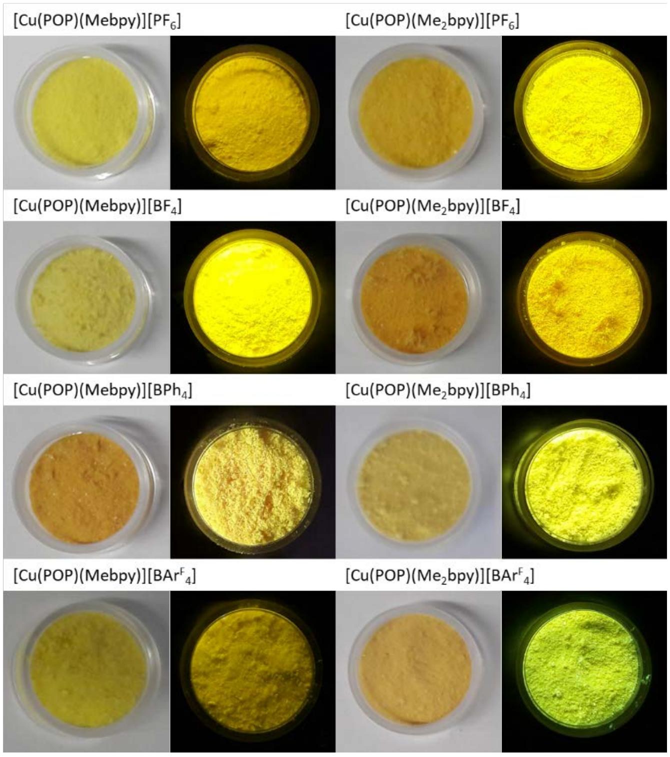

Fig. S79. Powder samples of [Cu(xantphos)(N^N)][A] complexes under ambient light (left) and under UV light (λ_{exc} = 366 nm, right).

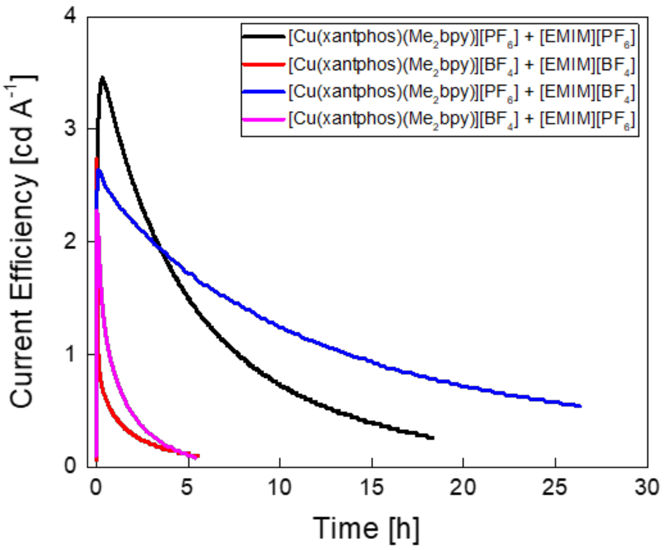

Fig. S80. Current efficiency of the best working LECs.

References

1. C. S. Smith, C. W. Branham, B. J. Marquardt and K. R. Mann, *J. Am. Chem. Soc.*, 2010, **132**, 14079-14085.

 This journal is © The Royal Society of Chemistry 20xx
